# Development of Novel and Efficient Processes for the Synthesis of 5-Amino and 5-Iminoimidazo[1,2-*a*]imidazoles via Three-Component Reaction Catalyzed by Zirconium(IV) Chloride

**DOI:** 10.3389/fchem.2019.00457

**Published:** 2019-07-08

**Authors:** Mohsine Driowya, Régis Guillot, Pascal Bonnet, Gérald Guillaumet

**Affiliations:** ^1^Institut de Chimie Organique et Analytique, Université d'Orléans, UMR CNRS 7311, Orléans, France; ^2^Institut de Chimie Moléculaire et des Matériaux d'Orsay, Université Paris Sud, Université Paris Saclay, UMR CNRS 8182, Orsay, France

**Keywords:** multicomponent reactions, isocyanide Ugi reaction, zirconium(IV) chloride, catalysis, N-heterocycles, fused-ring systems, 2-aminoimidazole

## Abstract

General and efficient approaches for the synthesis of new 5-amino and 5-iminoimidazo[1,2-*a*]imidazoles were developed through a three-component reaction of 1-unsubstituted 2-aminoimidazoles with various aldehydes and isocyanides mediated by zirconium(IV) chloride. The protocols were established considering the reactivity of the starting substrate, which varies depending on the presence of a substituent on the 2-aminoimidazole moiety. A library of new N-fused ring systems with wide structural diversification, novel synthetic, and potential pharmacological interest was obtained in moderate to good yields.

## Introduction

The development of innovative synthetic approaches that allow rapid access to a wide variety of new heterocyclic derivatives is of crucial interest. The use of multicomponent reactions (MCRs) offers significant advantages in organic synthesis, such as the combination of chemical transformations of three or more different starting materials in a one-pot procedure without isolating the intermediates (Dömling and Ugi, 2000; Dömling, 2006; Abdelraheem et al., 2017; Bariwal et al., 2018; Murlykina et al., 2018). In 1998, the groups of Groebke, Blackburn, and Bienayme simultaneously developed a new subclass of MCRs to produce a series of azine- and azole-fused aminoimidazoles, using diverse 2-aminoazines or 2-aminoazoles, aldehydes and isocyanides in the presence of Lewis or Brønsted acid catalysts (Bienayme and Bouzid, 1998; Blackburn, 1998; Groebke et al., 1998).

This reaction recently attracted much attention in organic and medicinal chemistry because of its simplicity, efficiency and the ability to generate diverse compound libraries (Devi et al., 2015; Kaur et al., 2016; Shaaban and Abdel-Wahab, 2016; Shaabani and Hooshmand, 2016).

The fused bicyclic 5-5 systems containing three nitrogen atoms with one in the bridgehead position are an important class of fused heterocyclic compounds in organic and medicinal chemistry due to their relevant biological properties, such as anti-cancer (Baviskar et al., 2011; Grosse et al., 2014; Sidduri et al., 2014; Meta et al., 2017), anti-viral (Elleder et al., 2012), anti-inflammatory (Bruno et al., 2007; Brullo et al., 2012), and anti-diabetic effects (Mascitti et al.). Our group has been involved over the last few years in the development of powerful tools for the synthesis of such systems (El Akkaoui et al., 2012; Grosse et al., 2012, 2013, 2014; Arnould et al., 2013; Tber et al., 2015a,b; Driowya et al., 2018). For instance, we very recently reported an efficient one-pot three component procedure for the synthesis of new functionalized imidazo[1,2-*b*]pyrazole derivatives in addition to a library of hitherto undescribed 7,7′-(substituted methylene)bis-imidazo[1,2-*b*]pyrazoles starting from 3-aminopyrazoles, various aldehydes and isocyanides, using a catalytic amount of perchloric acid or zirconium(IV) chloride (Driowya et al., 2018).

The imidazo-imidazole scaffolds hold a special place in this category, since they are found in compounds showing a wide range of pharmaceutical activities. In particular, they have been described as antifungal (Lila et al., 2003), antithrombotic (Imaeda et al., 2008; Fujimoto et al., 2009), anxiolytic and anti-depressive agents (Han et al., 2005; Tellew and Luo, 2008; Zuev et al., 2010). In addition, they have been reported as androgen receptor agonists and antagonists that are useful in the treatment of a variety of disorders (Zhang et al., 2006). Several synthetic methods based on multistep synthesis have been employed by our group and others to prepare these medicinally important N-fused imidazoles (Langer et al., 2001; Poje and Poje, 2003; Adib et al., 2008; Saima et al., 2012; Chen et al., 2013; Grosse et al., 2015; Castanedo et al., 2016; Loubidi et al., 2016; Kheder and Farghaly, 2017).

On the other hand, compounds containing an imidazo[1,2-*a*]imidazole moiety have been understudied (Compernolle and Toppet, 1986; Kolar and Tisler, 1995; Mas et al., 2002). Only one paper was found in the literature for the preparation of 5-aminoimidazo[1,2-*a*]imidazole compounds by MCRs starting from 1,5-disubstituted 2-aminoimidazoles (Pereshivko et al., 2013). However, the reported protocol gave poor to moderate yields and showed some limitations with 1-unsubstituted 2-aminoimidazole substrates. Hence, there is a need to develop a new, more efficient and general method for the preparation of these derivatives.

In this context, and in continuation of our ongoing search for innovative small molecules, we report herein novel and straightforward approaches for the synthesis of new series of 5-amino and 5-iminoimidazo[1,2-*a*]imidazoles starting from 1-unsubstituted 2-aminoimidazoles and using zirconium(IV) chloride as catalyst. To the best of our knowledge, this is the first report using 1-unsubstituted 2-aminoimidazoles in MCRs.

## Results And Discussion

In order to find a MCR protocol that can afford an efficient formation of 5-aminoimidazo[1,2-*a*]imidazoles, we initially performed a model reaction using ethyl 2-aminoimidazole-4-carboxylate **1** with benzaldehyde and *t*-octyl isocyanide under different conditions (Table 1). Two possible regioisomers can be formed in this case **4a** or **4a′** according on which side of the 2-aminoimidazole **1** that reacts.

**Table 1:** Optimization of the reaction conditions.

**Entry^**a**^**	**Catalyst (mol%)**	**Solvent**	**Temp (^**°**^C)**	**Heating method**	**Time**	**Yield (%)^**b**^**
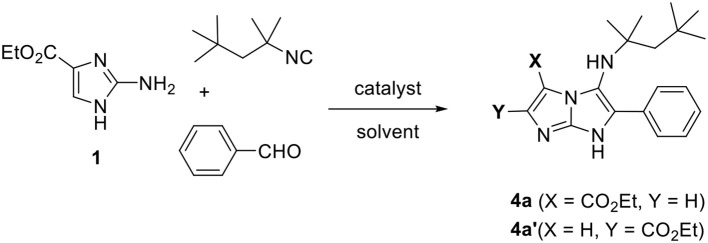						
1	ZrCl_4_ (5)	MeOH	r.t	–	14 h	0^c^
2	ZrCl_4_ (5)	EtOH	80	Conventional	14 h	12^c^
3	ZrCl_4_ (10)	EtOH	80	Conventional	14 h	18^c^
4	ZrCl_4_ (10)	EtOH	140	MW	10 min	38
5	ZrCl_4_ (10)	*n*-BuOH	140	MW	10 min	62
6	ZrCl_4_ (10)	PEG-400	140	MW	10 min	31
**7**	**ZrCl**_**4**_ **(10)**	**PEG-400**	**75**	**Conventional**	**4 h**	**77**
8	ZrCl_4_ (5)	PEG-400	75	Conventional	6 h	55
9	p-TsOH (10)	PEG-400	75	Conventional	4 h	58
10	ZnCl_2_ (10)	PEG-400	75	Conventional	7 h	23

a ***1** (1.0 mmol), benzaldehyde (1.1 mmol), t-octyl isocyanide (1.1 mmol), solvent (2 mL: entries 1–5; 1 mL: entries 6–10)*.

b *Yield of isolated product*.

c *Starting materials were recovered*.

*The bold values represent the optimized conditions*.

In our last study, we showed that the Lewis acid zirconium(IV) chloride delivered an efficient catalytic effect for the MCR (Driowya et al., 2018). This catalyst was therefore chosen for the present optimization study.

First, the reaction was carried out in methanol at room temperature in presence of 5 mol% of ZrCl_4_, but unfortunately, no product was observed (entry 1). Poor yields of the expected product **4a** or **4a′** were obtained when the reaction was performed under heating in ethanol with either 5 or 10 mol% of ZrCl_4_ (entries 2 and 3). The reaction time was significantly reduced to 10 min under MW irradiation at 140°C, and the yield was relatively improved to 38% (entry 4). The use of *n*-BuOH as solvent instead of EtOH under MW irradiation resulted in an improvement of the yield to 62% (entry 5), whereas the use of PEG-400 gave a moderate yield (entry 6). Interestingly, the reaction in PEG-400 under classical heating at 75°C during 4 h provided a very good yield (entry 7). Moreover, the optimal amount of catalyst (10 mol%) was confirmed, since the use of 5 mol% resulted in a lower yield (entry 8). Finally, replacing ZrCl_4_ by other catalysts such as *p*-TsOH or ZnCl_2_ was associated with a significant decrease in the yield of the product 5-aminoimidazo[1,2-*a*]imidazole **4a** or **4a′** (entries 9 and 10).

Hence, the optimized reaction conditions were found to be ZrCl_4_ (10 mol%) as catalyst and PEG-400 as solvent with heating at 75°C during 4 h. This system was used before for the preparation of imidazo[1,2-*a*]pyridines (Guchhait and Madaan, 2009).

**Figure 1:**
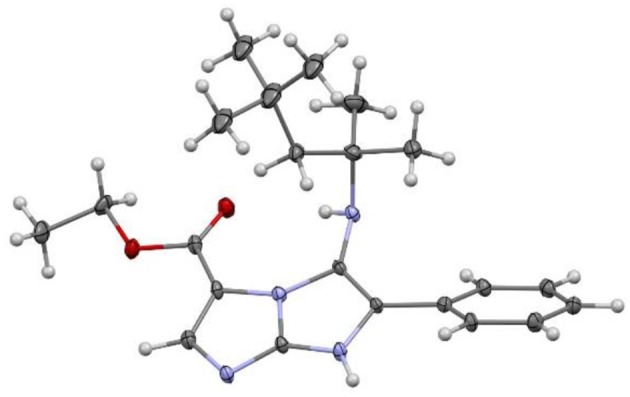
ORTEP representation of compound **4a**.

In order to disclose the structure of the formed regioisomer of this reaction, we carried out a single-crystal X-ray analysis of the product (Figure 1). The results reveal the formation of the regioisomer **4a**. The regioselectivity of this reaction can be explained by the presence of intermolecular hydrogen bonds on the ethyl 2-aminoimidazole-4-carboxylate **1**, orienting the synthesis toward the formation of only the regioisomer **4a**. Moreover, the structure of compound **4a** is stabilized by intramolecular N-H…O and intermolecular N-H…N interactions as observed in the crystalline structure (see the crystallographic section on the Supplementary Material).

With these reaction conditions in hand, we next explored the scope and limitation of our methodology with diverse 2-aminoimidazoles and isocyanides in the presence of a range of aldehydes as shown in Table 2. A large chemical library of 5-aminoimidazo[1,2-*a*]imidazole derivatives **4a–f** and **5a–i** was designed in generally good yields. The reactions proceeded well with both ethyl 2-aminoimidazole-4-carboxylate **1** and 4,5-dicyano-2-aminoimidazole **2**. Unfortunately, no reaction was observed when employing the unsubstituted 2-aminoimidazole **3** as substrate, which was recovered after purification.

**Table 2:** Synthesis of 5-aminoimidazo[1,2-*a*]imidazole derivatives **4a–f** and **5a–i**.

**Entry^**a**^**	**R^**1**^, R^**2**^**	**R^**3**^**	**R^**4**^**	**Product**	**Yield (%)^**b**^**
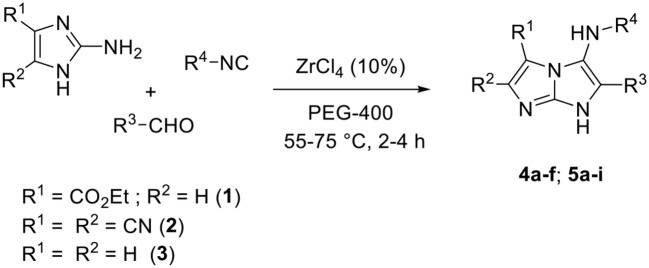					
1	CO_2_Et, H	C_6_H_5_	*t*-octyl	**4a**	77 (62)^c^
2	CO_2_Et, H	4-MeOC_6_H_4_	*t*-octyl	**4b**	68
3	CO_2_Et, H	4-ClC_6_H_4_	*t*-octyl	**4c**	56
4	CO_2_Et, H	4-CF_3_C_6_H_4_	*t*-octyl	**4d**	71
5	CO_2_Et, H	C_6_H_5_	*t*-butyl	**4e**	74
6	CO_2_Et, H	C_6_H_5_	cyclohexyl	**4f**	59 (50)^c^
7	CN, CN	C_6_H_5_	*t*-octyl	**5a**	67 (42)^c^
8	CN, CN	4-MeOC_6_H_4_	*t*-octyl	**5b**	65
9	CN, CN	2,4,6-MeOC_6_H_2_	*t*-octyl	**5c**	61
10	CN, CN	4-ClC_6_H_4_	*t*-octyl	**5d**	58
11	CN, CN	4-CF_3_C_6_H_4_	*t*-octyl	**5e**	76
12	CN, CN	C_2_H_5_	*t*-octyl	**5f**	12 (5)^c^
13	CN, CN	3-Pyridyl	*t*-octyl	**5g**	79
14	CN, CN	4-ClC_6_H_4_	*t*-butyl	**5h**	60 (54)^c^
15	CN, CN	4-ClC_6_H_4_	cyclohexyl	**5i**	47
16	H, H	4-MeOC_6_H_4_	*t*-octyl	**-**	0 (0)^c^

a ***1**, **2**, or **3** (1.0 mmol), aldehyde (1.1 mmol), isocyanide (1.1 mmol), ZrCl_4_ (0.1 mmol), PEG-400 (1 mL), 75°C, 4 h (entries 1–6, 16) or 55°C, 2 h (entries 7–15)*.

b *Yield of isolated product*.

c *Isolated yield using the conditions: ZrCl_4_ (10 mol%), n-BuOH (2 mL), MW (140°C), 10 min*.

Moreover, the reaction occurred with electron-withdrawing and electron-donating substituents of the benzaldehydes, in addition to sterically hindered aldehydes (entry 9), aliphatic and heteroaromatic aldehydes (entries 12 and 13, respectively). However, a poor yield was obtained when using propionaldehyde (entry 12).

The impact of isocyanide on our reaction was also investigated; the *tert*-octyl isocyanide and *tert*-butyl isocyanide gave similar good results, whereas the cyclohexyl isocyanide showed slightly lower yields.

The MCR involving the unsubstituted 2-aminoimidazole **3** using our conditions did not yield any product. This can be explained by the poor reactivity of the starting substrate due to the absence of an electron-withdrawing group.

In order to find another strategy allowing us to synthetize 5-aminoimidazo[1,2-*a*]imidazoles starting from the unsubstituted 2-aminoimidazole **3**, we first carried out the condensation of the latter with *p*-anisaldehyde as a first step model reaction under different conditions (Table 3). The free amine **3** was prepared from the commercially available 2-aminoimidazole sulfate (see Supplementary Material). Initially, conventional or MW heating of the reaction in different solvents with ZrCl_4_ (10 mol%) as catalyst provided the desired imine **6a** in poor yields (entries 1–4). The use of ZnCl_2_ as catalyst furnished a very low yield (entry 5), while *p*-TsOH and InCl_3_ gave slightly higher yields (entries 6 and 7). Very interestingly, using a reduced catalytic amount of InCl_3_ (2 mol% instead of 10 mol%) under conventional or MW heating in ethanol produced a real improvement in terms of yield and reaction time (entries 9 and 11). This may explain the non-reactivity observed in the MCR (Table 2, entry 16), because of the instability of the formed imine under such acidic conditions. However, the prolonged reaction time noted with ZrCl_4_ (2 mol%) or when no catalyst was used, revealed the influence of InCl_3_ on this condensation reaction (entries 8 and 10, respectively).

**Table 3:** Screening conditions for the synthesis of imine **6a**.

**Entry^**a**^**	**Catalyst (mol%)**	**Solvent**	**Temp (^**°**^C)**	**Heating method**	**Time (h)**	**Yield (%)^**b**^**
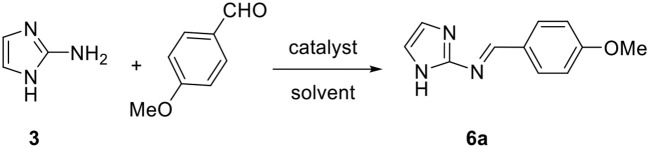						
1	ZrCl_4_ (10)	PEG-400	75	Conventional	16	31
2	ZrCl_4_ (10)	*n*-BuOH	140	MW	2	22
3	ZrCl_4_ (10)	Toluene	110	Conventional	16	15
4	ZrCl_4_ (10)	EtOH	80	Conventional	16	24
5	ZnCl_2_ (10)	EtOH	80	Conventional	16	5
6	p-TsOH (10)	EtOH	80	Conventional	16	53
7	InCl_3_ (10)	EtOH	80	Conventional	16	40
8	ZrCl_4_ (2)	EtOH	80	Conventional	16	61
9	**InCl**_**3**_ **(2)**	**EtOH**	**80**	Conventional	**2**	**78**
10	–	EtOH	80	Conventional	30	60
**11**	**InCl**_**3**_ **(2)**	**EtOH**	**100**	**MW**	**1**	**75**
12	InCl_3_ (2)	*n*-BuOH	100	MW	2	59

a ***3** (0.5 mmol), p-anizaldehyde (0.55 mmol)*.

b *Yield of isolated product*.

*The bold values represent the optimized conditions*.

After developing these optimized conditions for the first reaction step, we next focused on finding the best conditions for the second step, which is based on the [4+1] cycloaddition reaction of the formed imine with an isocyanide.

The resulting imine **6a** was isolated and reacted with *tert*-octyl isocyanide under several catalytic conditions (Table 4). No reaction occurred when using the same conditions as for the condensation step (entry 1). Increasing the catalytic amount of InCl_3_ to 10 mol% produced the desired product in poor yields with either conventional or MW heating methods (entries 2 and 3). It is interesting to note that the 5-aminoimidazo[1,2-*a*]imidazole product formed was unstable and underwent a dehydrogenation reaction *in situ* to generate the corresponding stable oxidized compound 5-iminoimidazo[1,2-*a*]imidazole **7a**. We already observed this type of oxidation in our previous work on the synthesis of imidazo[1,2-*b*]pyrazoles by MCRs (Driowya et al., 2018).

**Table 4:** Screening conditions for the synthesis of 5-iminoimidazo[1,2-*a*]imidazole **7a**.

**Entry^**a**^**	**Catalyst (mol%)**	**Solvent**	**Temp (^**°**^C)**	**Heating method**	**Time**	**Yield (%)^**b**^**
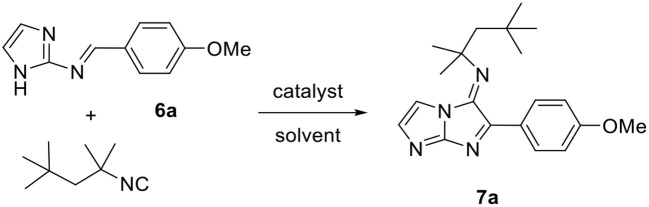						
1	InCl_3_ (2)	EtOH	80	Conventional	14 h	0
2	InCl_3_ (10)	EtOH	80	Conventional	6 h	<5
3	InCl_3_ (10)	EtOH	140	MW	10 min	14
4	p-TsOH (10)	EtOH	140	MW	10 min	19
5	ZnCl_2_ (10)	EtOH	140	MW	10 min	13
6	ZrCl_4_ (10)	EtOH	140	MW	10 min	22
7	ZrCl_4_ (10)	PEG-400	140	MW	10 min	26
**8**	**ZrCl**_**4**_ **(10)**	*n*-BuOH	**140**	MW	**10 min**	**40**
9	ZrCl_4_ (10)	*n*-BuOH	140	Conventional	3 h	<5
10	ZrCl_4_ (5)	*n*-BuOH	140	MW	10 min	31
11	InCl_3_ (10)	*n*-BuOH	140	MW	20 min	25

a ***6a** (0.2 mmol), tert-octyl isocyanide (0.22 mmol)*.

b *Yield of isolated product*.

*The bold values represent the optimized conditions*.

The use of other catalysts such as *p*-TsOH, ZnCl_2_, and ZrCl_4_ under microwave irradiation did not produce any significant improvement in the reaction yield (entries 4–6). However, using ZrCl_4_ as catalyst and replacing EtOH by *n*-BuOH as solvent for the reaction showed a slight increase in the yield to 40% (entry 8), which was the optimum result obtained for this reaction. The same conditions used under conventional heating resulted in a significant decrease in the yield. The low yield and the difficulty of this reaction can be explained by the instability of the imine in the acid medium.

With these optimized conditions in hand, we succeeded in achieving the one-pot two-step procedure without isolating the imine by removing EtOH at the end of the first reaction step. The isolated product **7a** was obtained with a global yield of 32%. This protocol was next extended to the synthesis of series of 5-iminoimidazo[1,2-*a*]imidazoles **7a–i** starting from the unsubstituted 2-aminoimidazole and exploring a wide range of aldehydes and isocyanides (Table 5). As mentioned previously, the 5-aminoimidazo[1,2-*a*]imidazole products formed were unstable and led directly to the corresponding imine forms **7a–i**. Despite the low yields obtained, it was nevertheless possible to produce the targeted compounds, which proved unsuccessful with the methods developed previously or with those cited in the literature.

**Table 5:** One-pot two-step synthesis of 5-iminoimidazo[1,2-*a*]imidazole derivatives **7a–i**.

**Entry^**a**^**	**R^**1**^**	**R^**2**^**	**Product**	**Yield (%)^**b**^**
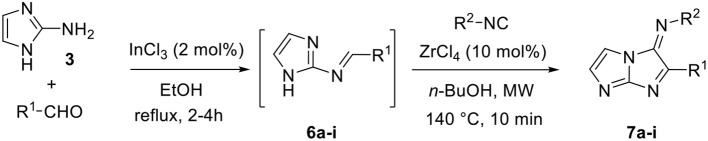				
1	4-MeOC_6_H_4_	*t*-octyl	**7a**	32
2	C_6_H_5_	*t*-octyl	**7b**	24
3	2,4,6-MeOC_6_H_2_	*t*-octyl	**7c**	35
4	4-ClC_6_H_4_	*t*-octyl	**7d**	19
5	4-CF_3_C_6_H_4_	*t*-octyl	**7e**	13
6	C_2_H_5_	*t*-octyl	**7f**	16
7	3-Pyridyl	*t*-octyl	**7g**	12
8	4-MeOC_6_H_4_	*t*-butyl	**7h**	17
9	4-MeOC_6_H_4_	cyclohexyl	**7i**	10

a ***3** (1.0 mmol), aldehyde (1.1 mmol), InCl_3_ (0.02 mmol), EtOH (10 mL), 2–4 h (90°C); isocyanide (1.1 mmol), ZrCl_4_ (0.1 mmol), n-BuOH (2 mL), 10 min (140°C)*.

b *Yield of isolated product*.

The chemical space of our synthetized compounds was then enlarged, by removing the *tert*-octyl groups of 5-aminoimidazo[1,2-*a*]imidazoles **4a–d** and **5a–g** using TFA as cleavage agent in DCM and giving access to the primary amine compounds **8a–d** and **9a–g**, respectively, with yields ranging from 26 to 79% (Table 6). In a similar way, the primary imine imidazo[1,2-*a*]imidazoles **10a**, **10b**, **10d**, **10e**, and **10g** were prepared in good yields from their corresponding Schiff base derivatives **7a, 7b, 7d, 7e**, and **7g** by deprotection of *tert*-octyl groups using the same conditions (Table 7). Unfortunately, the reaction was unsuccessful when the substituent R^1^ was 2,4,6-trimethoxyphenyl (entry 3) or ethyl (entry 6).

**Table 6:** Cleavage of the *t*-octyl group of the products **4a–d** and **5a–g**.

**Entry^**a**^**	**R^**1**^**	**R^**2**^**	**R^**3**^**	**Product**	**Yield (%)^**b**^**
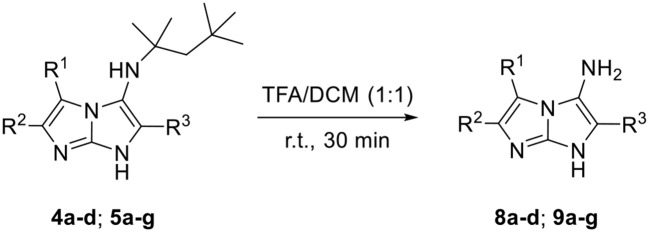					
1	CO_2_Et	H	C_6_H_5_	**8a**	70
2	CO_2_Et	H	4-MeOC_6_H_4_	**8b**	74
3	CO_2_Et	H	4-ClC_6_H_4_	**8c**	68
4	CO_2_Et	H	4-CF_3_C_6_H_4_	**8d**	65
5	CN	CN	C_6_H_5_	**9a**	59
6	CN	CN	4-MeOC_6_H_4_	**9b**	61
7	CN	CN	2,4,6-MeOC_6_H_2_	**9c**	32
8	CN	CN	4-ClC_6_H_4_	**9d**	57
9	CN	CN	4-CF_3_C_6_H_4_	**9e**	79
10	CN	CN	C_2_H_5_	**9f**	41
11	CN	CN	3-Pyridyl	**9g**	26

a ***4a–d**, **5a–g** (0.2 mmol), DCM/TFA 1:1 (5 mL), r.t*.

b *Isolated yield*.

**Table 7:** Dealkylation of the *t*-octyl group of the products **7a–g**.

**Entry^**a**^**	**R^**1**^**	**Product**	**Yield (%)^**b**^**
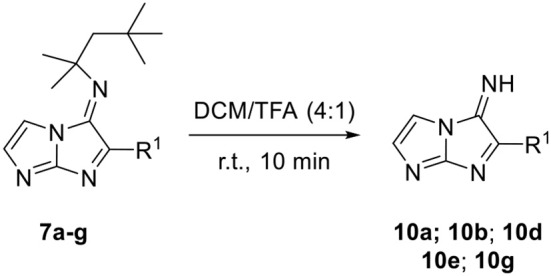			
1	4-MeOC_6_H_4_	**10a**	74
2	C_6_H_5_	**10b**	85
3	2,4,6-MeOC_6_H_2_	–	–
4	4-ClC_6_H_4_	**10d**	82
5	4-CF_3_C_6_H_4_	**10e**	63
6	C_2_H_5_	–	–
7	3-Pyridyl	**10g**	64

a ***7a–g** (0.15 mmol), DCM/TFA 4:1 (5 mL), r.t*.

b *Isolated yield*.

## Conclusion

In summary, we have designed highly efficient protocols of multicomponent isocyanide-based reactions catalyzed by zirconium(IV) chloride which offer the synthesis of a library of new functionalized 5-amino and 5-iminoimidazo[1,2-*a*]imidazoles in moderate to good yields. The optimized processes were successively applied to a large number of substituted (or unsubstituted) 2-aminoimidazoles, aldehydes and isocyanides. In addition, the use of inexpensive zirconium(IV) chloride as catalyst delivered an efficient catalytic effect for the reactions with a greater purity of isolated products compared to other catalysts.

## Data Availability

All datasets generated for this study are included in the manuscript and/or the Supplementary Files.

## Author Contributions

MD designed and performed the experiments, then was responsible for writing the manuscript. RG realised the X-ray analysis for the compound **4a**. PB and GG directed the project and revised the manuscript.

### Conflict of Interest Statement

The authors declare that the research was conducted in the absence of any commercial or financial relationships that could be construed as a potential conflict of interest.

## References

[B1] Abdelraheem E. M. M. Madhavachary R. Rossetti A. Kurpiewska K. Kalinowska-Tłuścik J. Shaabani S.. (2017). Ugi multicomponent reaction based synthesis of medium-sized rings. Org. Lett. 19, 6176–6179. 10.1021/acs.orglett.7b03094 29083197 PMC5698880

[B2] Adib M. Sheibani E. Zhu L.-G. Bijanzadeh H. R. (2008). Efficient synthesis of imidazo[2,1-b][1,3]benzothiazoles and 9H-imidazo-[1,2-a][1,3]benzimidazoles under solvent-free Conditions. Synlett 2008, 2941–2944. 10.1055/s-0028-1083623

[B3] Arnould M. Hiebel M.-A. Massip S. Léger J.-M. Jarry C. Berteina-Raboin S. (2013). Efficient metal-free synthesis of various pyrido[2′,1′:2,3]imidazo-[4,5-b]quinolones. Chem. Eur. J. 19, 12249–12253. 10.1002/chem.201300961 23955568

[B4] Bariwal J. Kaur R. Voskressensky L. G. Van der Eycken E. V. (2018). Post-Ugi cyclization for the construction of diverse heterocyclic compounds: recent updates. Front. Chem. 6:527. 10.3389/fchem.2018.00557 30525022 PMC6256255

[B5] Baviskar A. T. Madaan C. Preet R. Mohapatra P. Jain V. Agarwal A.. (2011). N-Fused imidazoles as novel anticancer agents that inhibit catalytic activity of topoisomerase IIα and induce apoptosis in G1/S phase. J. Med. Chem. 54, 5013–5030. 10.1021/jm200235u 21644529

[B6] Bienayme H. Bouzid K. (1998). A new heterocyclic multicomponent reaction for the combinatorial synthesis of fused 3-aminoimidazoles. Angew. Chem. Int. Ed. 37, 2234–2237. 29711433 10.1002/(SICI)1521-3773(19980904)37:16<2234::AID-ANIE2234>3.0.CO;2-R

[B7] Blackburn C. (1998). A three-component solid-phase synthesis of 3-aminoimidazo[1,2-a] azines. Tetrahedron Lett. 39, 5469–5472. 10.1016/S0040-4039(98)01113-7

[B8] Brullo C. Spisani S. Selvatici R. Bruno O. (2012). N-Aryl-2-phenyl-2,3-dihydro-imidazo[1,2-b]pyrazole-1-carboxamides 7-substituted strongly inhibiting both fMLP-OMe- and IL-8-induced human neutrophil chemotaxis. Eur. J. Med. Chem. 47, 573–579. 10.1016/j.ejmech.2011.11.031 22152986

[B9] Bruno O. Brullo C. Bondavalli F. Ranise A. Schenone S. Falzarano M. S.. (2007). 2-Phenyl-2,3-dihydro-1H-imidazo[1,2-b]pyrazole derivatives: new potent inhibitors of fMLP-induced neutrophil chemotaxis. Bioorg. Med. Chem. Lett. 17, 3696–3701. 10.1016/j.bmcl.2007.04.036 17475488

[B10] Castanedo G. Liu Y. Crawford J. J. Braun M.-G. (2016). Synthesis of fused imidazole-containing ring systems via dual oxidative amination of C(sp^3^)–H bonds. J. Org. Chem. 81, 8617–8624. 10.1021/acs.joc.6b01517 27529722

[B11] Chen F. Lei M. Hu L. (2013). Thiamine hydrochloride (VB1)-catalyzed one-pot synthesis of (E)-N-benzylidene-2-phenyl-1H-benzo[d]imidazo[1,2-a]imidazol-3-amine derivatives. Tetrahedron 69, 2954–2960. 10.1016/j.tet.2013.02.022

[B12] Compernolle F. Toppet S. J. (1986). Synthesis of 1H-imidazo[1,2-a]imidazole. J. Heterocycl. Chem. 23, 541–544. 10.1002/jhet.5570230246

[B13] Devi N. Rawal R. K. Singh V. (2015). Diversity-oriented synthesis of fused-imidazole derivatives via Groebke–Blackburn–Bienayme reaction: a review. Tetrahedron 71, 183–232. 10.1016/j.tet.2014.10.032

[B14] Dömling A. (2006). Recent developments in isocyanide based multicomponent reactions in applied chemistry. Chem. Rev. 106, 17–89. 10.1021/cr0505728 16402771

[B15] Dömling A. Ugi I. (2000). Multicomponent reactions with isocyanides. Angew. Chem. Int. Ed. 39, 3168–3210. 10.1002/1521-3773(20000915)39:18<3168::AID-ANIE3168>3.0.CO;2-U 11028061

[B16] Driowya M. Allouchi H. Gally J. M. Bonnet P. Guillaumet G. (2018). Synthesis of novel series of 7,7′-(substituted methylene)bis-imidazo[1,2-b]pyrazoles via an acid catalyzed one-pot three-component reaction. New J. Chem. 42, 5728–5741. 10.1039/C7NJ05088G

[B17] El Akkaoui A. Hiebel M.-A. Mouaddib A. Berteina-Raboin S. Guillaumet G. (2012). Straightforward Bienaymé and copper catalyzed N-arylation sequence to access diverse 5H-pyrido[2′,1′:2,3]imidazo[4,5-b]indoles and analogues. Tetrahedron 68, 9131–9138. 10.1016/j.tet.2012.07.081

[B18] Elleder D. Baiga T. J. Russell R. L. Naughton J. A. Hughes S. H. Noel J. P.. (2012). Identification of a 3-aminoimidazo[1,2-a]pyridine inhibitor of HIV-1 reverse transcriptase. Virol. J. 9, 305–311. 10.1186/1743-422X-9-305 23231773 PMC3560175

[B19] Fujimoto T. Tobisu M. Konishi N. Kawamura M. Tada N. Takagi T.. (2009). Synthesis and biological evaluation of the metabolites of 2-(1-{3-[(6-chloronaphthalen-2-yl)sulfonyl]propanoyl} piperidin-4-yl)-5-methyl-1,2-dihydro-3H-imidazo[1,5-c]imidazol-3-one. Bioorg. Med. Chem. 17, 7993–8002. 10.1016/j.bmc.2009.10.009 19861238

[B20] Groebke K. Weber L. Mehlin F. (1998). Synthesis of imidazo[1,2-a] annulated pyridines, pyrazines and pyrimidines by a novel three-component condensation. Synlett 1998, 661–664. 10.1055/s-1998-1721

[B21] Grosse E. S. Pillard C. Bernard P. Guillaumet G. (2013). Efficient C-7 or C-3/C-7 direct arylation of tri- or disubstituted imidazo[1,2-b]pyrazoles. Synlett. 24, 2095–2101. 10.1055/s-0033-1339657

[B22] Grosse S. Mathieu V. Pillard C. Massip S. Marchivie M. Jarry C.. (2014). New imidazo[1,2-b]pyrazoles as anticancer agents: synthesis, biological evaluation and structure activity relationship analysis. Eur. J. Med. Chem. 84, 718–730. 10.1016/j.ejmech.2014.07.057 25064349

[B23] Grosse S. Pillard C. Massip S. Léger J. M. Jarry C. Bourg S.. (2012). Efficient synthesis and first regioselective C-3 direct arylation of imidazo[1,2-b]pyrazoles. Chemistry 18, 14943–14947. 10.1002/chem.201202593 23086664

[B24] Grosse S. Pillard C. Massip S. Marchivie M. Jarry C. Bernard P.. (2015). Ligandless palladium-catalyzed regioselective direct C–H arylation of imidazo[1,2-a]imidazole derivatives. J. Org. Chem. 80, 8539–8551. 10.1021/acs.joc.5b00534 26243437

[B25] Guchhait S. K. Madaan C. (2009). An efficient, regioselective, versatile synthesis of N-fused 2- and 3-aminoimidazoles via Ugi-type multicomponent reaction mediated by zirconium(IV) chloride in polyethylene glycol-400. Synlett 4, 628–632. 10.1055/s-0028-1087915

[B26] Han X. Michne J. A. Pin S. S. Burris K. D. Balanda L. A. Fung L. K.. (2005). Synthesis, structure–activity relationships, and anxiolytic activity of 7-aryl-6,7-dihydroimidazoimidazole corticotropin-releasing factor 1 receptor antagonists. Bioorg. Med. Chem. Lett. 15, 3870–3873. 10.1016/j.bmcl.2005.05.117 15990298

[B27] Imaeda Y. Kuroita T. Sakamoto H. Kawamoto T. Tobisu M. Konishi N.. (2008). Discovery of imidazo[1,5-c]imidazol-3-ones: weakly basic, orally active factor Xa inhibitors. J. Med. Chem. 51, 3422–3436. 10.1021/jm701548u 18507371

[B28] Kaur T. Wadhwa P. Bagchi S. Sharma A. (2016). Isocyanide based [4+1] cycloaddition reactions: an indispensable tool in multi-component reactions (MCRs). Chem. Commun. 52, 6958–6976. 10.1039/C6CC01562J 27063921

[B29] Kheder N. A. Farghaly T. A. R. (2017). Bis-Hydrazonoyl chloride as precursors for synthesis of novel polysubstituted bis-azoles. Arab. J. Chem. 10, S3007–S3014. 10.1016/j.arabjc.2013.11.040

[B30] Kolar P. Tisler M. (1995). A new synthetic approach for imidazo[1,2-a]imidazoles and pyrrolo[1,2-a]imidazoles. J. Heterocycl. Chem. 32, 141–144. 10.1002/jhet.5570320123

[B31] Langer P. Wuckelt J. Döring M. Schreiner P. R. Görls H. (2001). Regioselective anionic [3+2] cyclizations of imidazole dinucleophiles with oxaldiimidoyl dichlorides – a combined experimental and theoretical study. Eur. J. Org. Chem. 2245–2255. 10.1002/1099-0690(200106)2001:12<2245::AID-EJOC2245>3.0.CO;2-C

[B32] Lila T. Renau T. E. Wilson L. Philips J. Natsoulis G. Cope M. J.. (2003). Molecular basis for fungal selectivity of novel antimitotic compounds. Antimicrob. Agents Chemother. 47, 2273–2282. 10.1128/AAC.47.7.2273-2282.2003 12821479 PMC161869

[B33] Loubidi M. Pillard C. El Hakmaoui A. Bernard P. Akssira M. Guillaumet G. (2016). A new synthetic approach to the imidazo[1,5-a]imidazole-2-one scaffold and effective functionalization through Suzuki–Miyaura cross coupling reactions. RSC Adv. 6, 7229–7238. 10.1039/C5RA25520A

[B34] Mas T. Claramunt R. M. Santa María M. D. Sanz D. Alarcón S. H. Pérez-Torralba M. (2002). Structure and spectroscopy of imidazo [1,2-a]imidazoles and imidazo[1,2-a]benzimidazoles. Arkivoc 2002, 48–61. 10.3998/ark.5550190.0003.507

[B35] Mascitti V. McClure K. F. Munchhof M. J. Robinson R. P. (2011). Imidazo-pyrazoles as GPR119 Inhibitors. WO Patent 2011/061679 A1.

[B36] Meta E. Brullo C. Sidibe A. Imhof B. A. Bruno O. (2017). Design, synthesis and biological evaluation of new pyrazolyl-ureas and imidazopyrazolecarboxamides able to interfere with MAPK and PI3K upstream signaling involved in the angiogenesis. Eur. J. Med. Chem. 133, 24–35. 10.1016/j.ejmech.2017.03.066 28371678

[B37] Murlykina M. V. Morozova A. D. Zviagin I. M. Sakhno Y. I. Desenko S. M. Chebanov V. A. (2018). Aminoazole-based diversity-oriented synthesis of heterocycles. Front. Chem. 6:527. 10.3389/fchem.2018.00527 30555815 PMC6282055

[B38] Pereshivko O. P. Peshkov V. A. Ermolat'ev D. S. Van der Eycken E. V. (2013). Fast assembly of 1H-imidazo[1,2-a]imidazol-5-amines via Groebke–Blackburn–Bienaymé reaction with 2-aminoimidazoles. Synlett 24, 351–354. 10.1055/s-0032-1317986

[B39] Poje N. Poje M. (2003). An unusual oxidative ring transformation of purine to imidazo[1,5-c]imidazole. Org. Lett. 5, 4265–4268. 10.1021/ol035429k 14601976

[B40] Saima Y. Khamarui S. Gayen K. S. Pandit P. Maiti D. K. (2012). Efficient catalytic cyclizations of three and two imine assemblies: direct access to tetrahydroimidazo[1,5-c]imidazol-7-ones and imidazoles. Chem. Commun. 48, 6601–6603. 10.1039/c2cc32760k 22627840

[B41] Shaaban S. Abdel-Wahab B. F. (2016). Groebke–Blackburn–Bienaymé multicomponent reaction: emerging chemistry for drug discovery. Mol. Divers. 20, 233–254. 10.1007/s11030-015-9602-6 26016721

[B42] Shaabani A. Hooshmand S. E. (2016). Choline chloride/urea as a deep eutectic solvent/organocatalyst promoted three-component synthesis of 3-aminoimidazo-fused heterocycles via Groebke–Blackburn–Bienayme process. Tetrahedron Lett. 57, 310–313. 10.1016/j.tetlet.2015.12.014

[B43] Sidduri A. Budd D. C. Fuentes M. E. Lambros T. Ren Y. Roongta V.. (2014). Discovery of novel non-carboxylic acid 5-amino-4-cyanopyrazole derivatives as potent and highly selective LPA1R antagonists. Bioorg. Med. Chem. Lett. 24, 4450–4454. 10.1016/j.bmcl.2014.08.001 25155385

[B44] Tber Z. Hiebel M.-A. Allouchi H. El Hakmaoui A. Akssira M. Guillaumet G. (2015a). Metal free direct formation of various substituted pyrido[2′,1′:2,3]imidazo[4,5-c]isoquinolin-5-amines and their further functionalization. RSC Adv. 5, 35201–35210. 10.1039/C5RA03703D

[B45] Tber Z. Hiebel M.-A. El Hakmaoui A. Akssira M. Guillaumet G. Berteina-Raboin S. (2015b). Metal free formation of various 3-iodo-1H-pyrrolo[3′,2′:4,5]imidazo-[1,2-a]pyridines and [1,2-b]pyridazines and their further functionalization J. Org. Chem. 80, 6564–6573. 10.1021/acs.joc.5b00555 26083102

[B46] Tellew J. E. Luo Z. (2008). Small molecule antagonists of the corticotropin releasing factor (CRF) receptor: recent medicinal chemistry developments. Curr. Top. Med. Chem. 8, 506–520. 10.2174/156802608783955665 18397172

[B47] Zhang X. Allan G. F. Sbriscia T. Linton O. Lundeen S. G. Sui Z. (2006). Synthesis and SAR of novel hydantoin derivatives as selective androgen receptor modulators. Bioorg. Med. Chem. Lett. 16, 5763–5766. 10.1016/j.bmcl.2006.08.084 16959487

[B48] Zuev D. Vrudhula V. M. Michne J. A. Dasgupta B. Pin S. S. Huang X. S.. (2010). Discovery of 6-chloro-2-trifluoromethyl-7-aryl-7H-imidazo[1,2-a]imidazol-3-ylmethylamines, a novel class of corticotropin-releasing factor receptor type 1 (CRF1R) antagonists. Bioorg. Med. Chem. Lett. 20, 3669–3674. 10.1016/j.bmcl.2010.04.094 20471832

