# Supplementary material for: Development of Novel and Efficient Processes for the Synthesis of 5-Amino and 5-Iminoimidazo[1,2-a]imidazoles via Three-Component Reaction Catalyzed by Zirconium(IV) Chloride
Source: Front Chem. 2019 Jul 8;7:457. doi: 10.3389/fchem.2019.00457 (PMC6628877; doi:10.3389/fchem.2019.00457)
Supplement: Supplementary file 1 [file Data_Sheet_1.pdf]

Supplementary Material

Development of Novel and Efficient Processes for the Synthesis of 5-Amino and 5-Iminoimidazo[1,2-*a*]imidazoles via Three-Component Reaction Catalyzed by Zirconium(IV) Chloride

Mohsine Driowya,[a] Régis Guillot,[b] Pascal Bonnet[a] and Gérald Guillaumet*[a]

[a] Institut de Chimie Organique et Analytique, Université d’Orléans, UMR CNRS 7311, BP 6759, 45067 Orléans Cedex 2, France.

[b] Institut de Chimie Moléculaire et des Matériaux d'Orsay, Université Paris Sud, Université Paris Saclay, UMR CNRS 8182, 15 rue Georges Clémenceau, 91405 Orsay cedex, France.

Table of Contents

General Information	S2
General procedure for the synthesis of compounds **4a-f** and **5a-i**	S3
General procedure for the synthesis of compounds **7a-i**	S11
General procedure for the synthesis of compounds **8a-d, 9a-g**	S16
General procedure for the synthesis of compounds **10a, 10b, 10d, 10e, 10g**	S21
^{1}H and ^{13}C spectrum of compounds **4a-f** and **5a-i**	S24
^{1}H and ^{13}C spectrum of compounds **7a-i**	S41
^{1}H and ^{13}C spectrum of compounds **8a-d, 9a-g**	S51
^{1}H and ^{13}C spectrum of compounds **10a, 10b, 10d, 10e, 10g**	S64
X-ray analysis of compound **4a**	S70

General information

All reagents were purchased from commercial suppliers and were used without further purification. The reactions were monitored by thin-layer chromatography (TLC) analysis using silica gel (60 F254) plates. Compounds were visualized under UV light. Flash column chromatography was performed on silica gel 60 (230–400 mesh, 0.040–0.063 mm). Melting points were measured with Electrothermal IA9100 apparatus and were uncorrected. The infrared spectra of compounds were recorded on a Thermo Scientific Nicolet iS10. Absorption bands are given in cm^{-1}. ^{1}H and ^{13}C NMR spectra were recorded on a Bruker Avance II 400 MHz (^{13}C, 100.6 MHz) or on a Bruker Avance DPX 250 MHz (^{13}C, 62.9 MHz). Chemical shifts are given in parts per million from tetramethylsilane (TMS) as internal standard. Coupling constants (J) are reported in Hertz (Hz). High-resolution mass spectra (HRMS) were performed on a Maxis Bruker 4G.

General procedure for the synthesis of compounds 4a-f and 5a-i

In a closed vessel containing a solution of substituted 2-aminoimidazole **1** or **2** (1.0 mmol) in PEG-400 (1 mL), aldehyde (1.1 mmol) and $ZrCl_4$ (0.10 mmol) were sequentially added at room temperature followed by the addition of isocyanide (1.1 mmol). The reaction was heated at 75 °C and 55 °C, respectively, for the ethyl 2-aminoimidazole-4-carboxylate **1** and 4,5-dicyano-2-aminoimidazole **2**. Once the reaction was completed (controlled by TLC), the mixture was poured into water (15 mL) then extracted with ethyl acetate (2 × 15 mL). The extract was washed with water and brine, dried over anhydrous Na_2SO_4, filtered and concentrated under reduced pressure. The crude material was purified by flash chromatography on silica gel using petroleum ether/ethyl acetate mixture (3:2 v/v) as eluent to provide the expected pure products **4a-f** and **5a-i**.

Ethyl 6-phenyl-5-((2,4,4-trimethylpentan-2-yl)amino)-1*H*-imidazo[1,2-*a*]imidazole-3-carboxylate (4a)

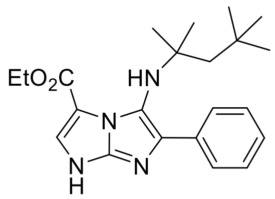

4a

Yellow solid (295 mg, 77%); m.p.: 176-178 °C; IR (neat, cm^{-1}): 3330 (NH), 2955, 2901, 1699 (CO), 1602, 1516, 1249, 1192, 1112; ^{1}H NMR (400 MHz, DMSO-d_6): δ 12.18 (br s, 1H, NHimidazole), 7.86 (d, *J* = 7.3 Hz, 2H, HAr), 7.71 (s, 1H, Himidazole), 7.42 (t, *J* = 7.7 Hz, 2H, HAr), 7.29 (t, *J* = 7.4 Hz, 1H, HAr), 4.62 (s, 1H, NH), 4.29 (q, *J* = 7.1 Hz, 2H, CH_2ester), 1.46 (s, 2H, CH_2), 1.31 (t, *J* = 7.1 Hz, 3H, CH_3ester), 0.97 (s, 9H, 3CH_3), 0.83 (s, 6H, 2CH_3); ^{13}C NMR (101 MHz, DMSO-d_6): δ 161.1, 147.4, 141.4, 131.7, 128.7, 127.8, 127.5, 123.3, 122.2, 114.1, 61.6, 60.4, 55.5, 32.1, 28.7, 14.9; HRMS (ESI): (m/z) calcd for $C_{22}H_{31}N_4O_2$ $[M + H]^+$: 383.2441, found: 383.2439.

Ethyl 6-(4-methoxyphenyl)-5-((2,4,4-trimethylpentan-2-yl)amino)-1*H*-imidazo[1,2-*a*]imidazole-3-carboxylate (4b)

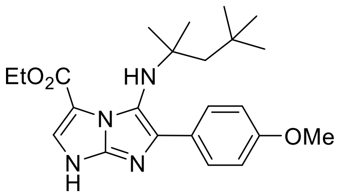

4b

Yellow solid (280 mg, 68%); m.p.: 182-183 °C; IR (neat, cm^{-1}): 3342 (NH), 2956, 2900, 1698 (CO), 1603, 1571, 1248, 1191, 1113; ^{1}H NMR (400 MHz, DMSO-d_6): δ 12.08 (br s, 1H, NHimidazole), 7.78 (d, *J* = 8.8 Hz, 2H, HAr), 7.68 (s, 1H, Himidazole), 6.99 (d, *J* = 8.8 Hz, 2H, HAr), 4.55 (s, 1H, NH), 4.29 (q, *J* = 7.1 Hz, 2H, CH_2ester), 3.79 (s, 3H, OCH_3), 1.46 (s, 2H, CH_2), 1.30 (t, *J* = 7.0 Hz, 3H, CH_3ester), 0.97 (s, 9H, 3CH_3), 0.83 (s, 6H, 2CH_3); ^{13}C NMR (101 MHz, DMSO-d_6): δ 161.1, 159.0, 147.2, 141.3, 128.9, 124.0, 123.3, 121.3, 114.2, 114.0, 61.4, 60.3, 55.6, 55.5, 32.1, 31.8, 28.7, 15.0; HRMS (ESI): (m/z) calcd for $C_{23}H_{33}N_4O_3$ $[M + H]^+$: 413.2547, found: 413.2546.

Ethyl 6-(4-chlorophenyl)-5-((2,4,4-trimethylpentan-2-yl)amino)-1*H*-imidazo[1,2-*a*]imidazole-3-carboxylate (4c)

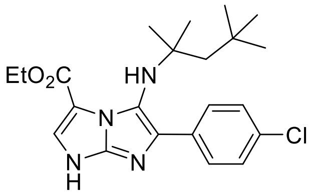

4c

White solid (235 mg, 56%); m.p.: 226-228 °C; IR (neat, cm^{-1}): 3336 (NH), 2954, 2902, 1699 (CO), 1602, 1579, 1512, 1375, 1243, 1188; ^{1}H NMR (400 MHz, Acetic acid-d_4): δ 8.08–7.87 (m, 3H, 2HAr and Himidazole), 7.48 (t, *J* = 9.6 Hz, 2H, HAr), 4.52–4.40 (m, 2H, CH_2ester), 1.59 (d, *J* = 14.8 Hz, 2H, CH_2), 1.43 (dt, *J* = 13.8, 7.2 Hz, 3H, CH_3ester), 1.05 (s, 9H, 3CH_3), 0.96 (s, 6H, 2CH_3); ^{13}C NMR (101 MHz, Acetic acid-d_4): δ 160.1, 139.8, 134.4, 133.8, 130.0, 129.2, 128.8, 128.7, 128.6, 127.9, 127.8, 126.5, 124.5, 123.8, 120.7, 115.6, 112.1, 61.8, 61.7, 61.6, 60.0, 55.9, 55.3, 31.2, 31.1, 31.0, 27.9, 13.5; HRMS (ESI): (m/z) calcd for $C_{22}H_{30}ClN_4O_2$ $[M + H]^+$: 417.2051, found: 417.2047.

Ethyl 6-(4-(trifluoromethyl)phenyl)-5-((2,4,4-trimethylpentan-2-yl)amino)-1*H*-imidazo[1,2-*a*]imidazole-3-carboxylate (4d)

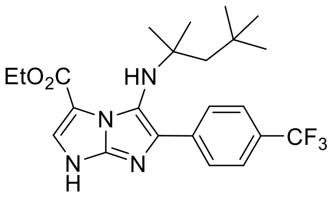

4d

White solid (320 mg, 71%); m.p.: 193-195 °C; IR (neat, cm^{-1}): 3291 (NH), 2962, 2909, 1668 (CO), 1616, 1486, 1320, 1280, 1190; ^1H NMR (400 MHz, Acetic acid-d_4): δ 8.17 (d, *J* = 8.2 Hz, 2H, HAr), 8.01 (s, 1H, Himidazole), 7.78 (d, *J* = 8.2 Hz, 2H, HAr), 4.48 (q, *J* = 7.1 Hz, 2H, CH_2ester), 1.58 (s, 2H, CH_2), 1.45 (t, *J* = 7.1 Hz, 3H, CH_3ester), 1.05 (s, 9H, 3CH_3), 0.98 (s, 6H, 2CH_3); ^{13}C NMR (101 MHz, Acetic acid-d_4): δ 160.2, 140.4, 134.3, 130.5, 129.4 (q, *J* = 32.2 Hz), 128.2, 128.1, 125.5, 125.3 (q, *J* = 3.8 Hz), 124.6, 124.3, 122.8 (q, *J* = 271.0 Hz), 115.6, 61.8, 61.7, 55.4, 31.1, 31.0, 27.8, 13.5; ^{19}F NMR (376 MHz, Acetic acid-d_4): δ -63.4 (s); HRMS (ESI): (m/z) calcd for $C_{23}H_{30}F_3N_4O_2$ [M + H]$^+$: 451.2315, found: 451.2315.

Ethyl 5-(*tert*-butylamino)-6-phenyl-1*H*-imidazo[1,2-*a*]imidazole-3-carboxylate (4e)

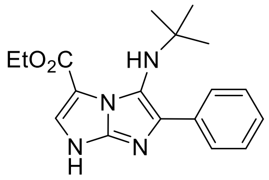

4e

White solid (240 mg, 74%); m.p.: 198-199 °C; IR (neat, cm^{-1}): 3333 (NH), 2959, 2900, 1698 (CO), 1600, 1515, 1249, 1199, 1106; ^1H NMR (400 MHz, DMSO-d_6): δ 12.19 (br s, 1H, NHimidazole), 7.92 (d, *J* = 7.5 Hz, 2H, HAr), 7.72 (s, 1H, Himidazole), 7.41 (t, *J* = 7.8 Hz, 2H, HAr), 7.27 (t, *J* = 7.6 Hz, 1H, HAr), 4.70 (s, 1H, NH), 4.29 (q, *J* = 7.0 Hz, 2H, CH_2ester), 1.30 (t, *J* = 7.0 Hz, 3H, CH_3ester), 0.91 (s, 9H, 3CH_3); ^{13}C NMR (101 MHz, DMSO-d_6): δ 161.0, 147.4, 141.4, 131.5, 128.7, 127.7, 127.2, 123.3, 122.6, 114.0, 60.3, 57.5, 29.5, 14.9; HRMS (ESI): (m/z) calcd for $C_{18}H_{23}N_4O_2$ [M + H]$^+$: 327.1816, found: 327.1817.

Ethyl 5-(cyclohexylamino)-6-phenyl-1*H*-imidazo[1,2-*a*]imidazole-3-carboxylate (4f)

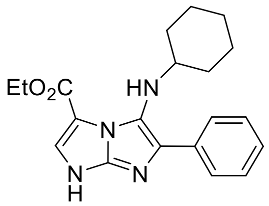

4f

White solid (206 mg, 59%); m.p.: 215-216 °C; IR (neat, cm^{-1}): 3314 (NH), 2931, 2858, 1682 (CO), 1610, 1548, 1418, 1285, 1227, 1109, 1021; ^1H NMR (400 MHz, Acetic acid-d_4): δ 7.99 (s, 1H, Himidazole), 7.90 (d, *J* = 7.3 Hz, 2H, HAr), 7.48 (t, *J* = 7.7 Hz, 2H, HAr), 7.36 (t, *J* = 7.4 Hz, 1H, HAr), 4.46 (q, *J* = 7.2 Hz, 2H, CH_2ester), 2.91 (ddd, *J* = 10.4, 6.6, 3.7 Hz, 1H, CH), 1.82–1.90 (m, 2H, CH_2), 1.73–1.64 (m, 2H, CH_2), 1.51–1.57 (m, 1H, ½CH_2), 1.44 (t, *J* = 7.2 Hz, 3H, CH_3ester), 1.27–1.10 (m, 5H, (2+½)CH_2); ^{13}C NMR (101 MHz, Acetic acid-d_4): δ 160.1, 138.9, 129.7, 129.0, 128.7, 128.6, 127.8, 126.5, 126.1, 126.0, 119.3, 115.2, 111.8, 61.8, 56.8, 33.4, 33.1, 25.4, 24.7, 13.4; HRMS (ESI): (m/z) calcd for $C_{20}H_{25}N_4O_2$ $[M + H]^+$: 353.1972, found: 353.1972.

6-Phenyl-5-((2,4,4-trimethylpentan-2-yl)amino)-1*H*-imidazo[1,2-*a*]imidazole-2,3-dicarbonitrile (5a)

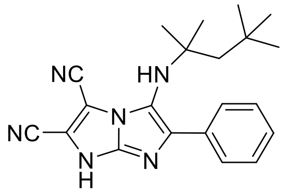

5a

White solid (240 mg, 67%); m.p.: 237-239 °C; IR (neat, cm^{-1}): 3383 (NH), 2956, 2904, 2208 (CN), 1614, 1566, 1382, 1180; ^1H NMR (400 MHz, DMSO-d_6): δ 12.94 (br s, 1H, NHimidazole), 7.84 (d, *J* = 7.5 Hz, 2H, HAr), 7.49 (t, *J* = 7.5 Hz, 2H, HAr), 7.46–7.40 (m, 1H, HAr), 4.60 (s, 1H, NH), 1.45 (s, 2H, CH_2), 0.96 (s, 6H, 2CH_3), 0.89 (s, 9H, 3CH_3); ^{13}C NMR (101 MHz, DMSO-d_6): δ 144.8, 130.5, 129.7, 129.2, 129.0, 128.6, 123.4, 121.0, 114.1, 111.3, 97.7, 59.5, 56.2, 31.9, 31.4, 28.7; HRMS (ESI): (m/z) calcd for $C_{21}H_{25}N_6$ $[M + H]^+$: 361.2135, found: 361.2137, (m/z) calcd for $C_{21}H_{24}N_6Na$ $[M + Na]^+$: 383.1955, found: 383.1954.

6-(4-Methoxyphenyl)-5-((2,4,4-trimethylpentan-2-yl)amino)-1*H*-imidazo[1,2-*a*]imidazole-2,3-dicarbonitrile (5b)

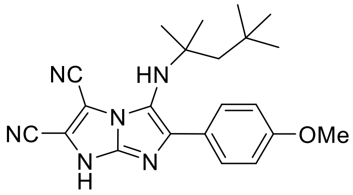

5b

White solid (255 mg, 65%); m.p.: 228-229 °C; IR (neat, cm^{-1}): 3362 (NH), 2951, 2902, 2213 (CN), 1618, 1560, 1370, 1179, 1022; ^1H NMR (400 MHz, DMSO-d_6): δ 12.83 (br s, 1H, NHimidazole), 7.84 (d, J = 8.3 Hz, 2H, HAr), 7.05 (d, J = 8.3 Hz, 2H, HAr), 4.54 (s, 1H, NH), 3.81 (s, 3H, OCH_3), 1.46 (s, 2H, CH_2), 0.96 (s, 6H, 2CH_3), 0.91 (s, 9H, 3CH_3); ^{13}C NMR (101 MHz, DMSO-d_6): δ 160.1, 144.6, 130.6, 130.0, 123.1, 121.9, 120.3, 114.4, 114.2, 111.4, 97.5, 59.4, 56.2, 55.7, 31.9, 31.4, 28.7; HRMS (ESI): (m/z) calcd for $C_{22}H_{27}N_6O$ $[M + H]^+$: 391.2241, found: 391.2241, (m/z) calcd for $C_{22}H_{26}N_6NaO$ $[M + Na]^+$: 413.2060, found: 413.2063.

6-(2,4,6-Trimethoxyphenyl)-5-((2,4,4-trimethylpentan-2-yl)amino)-1*H*-imidazo[1,2-*a*]imidazole-2,3-dicarbonitrile (5c)

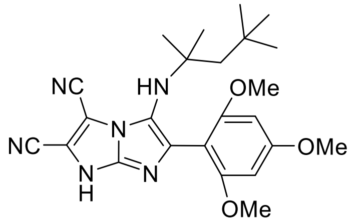

5c

Yellow solid (255 mg, 61%); m.p.: 224-225 °C; IR (neat, cm^{-1}): 3369 (NH), 2954, 2893, 2226 (CN), 1608, 1572, 1253, 1171, 1030; ^1H NMR (400 MHz, DMSO-d_6): δ 12.51 (br s, 1H, NHimidazole), 6.39 (s, 2H, HAr), 3.86 (s, 3H, OCH_3), 3.81 (s, 6H, 2OCH_3), 3.36 (s, 1H, NH), 1.35 (s, 2H, CH_2), 0.94 (s, 6H, 2CH_3), 0.89 (s, 9H, 3CH_3); ^{13}C NMR (101 MHz, DMSO-d_6): δ 163.3, 159.3, 144.8, 123.3, 123.1, 122.5, 114.3, 111.4, 99.1, 96.8, 91.5, 58.6, 56.5, 56.0, 55.7, 31.8, 31.5, 28.5; HRMS (ESI): (m/z) calcd for $C_{24}H_{31}N_6O_3$ $[M + H]^+$: 451.2452, found: 451.2451, (m/z) calcd for $C_{24}H_{30}N_6NaO_3$ $[M + Na]^+$: 473.2272, found: 473.2270.

6-(4-Chlorophenyl)-5-((2,4,4-trimethylpentan-2-yl)amino)-1*H*-imidazo[1,2-*a*]imidazole-2,3-dicarbonitrile (5d)

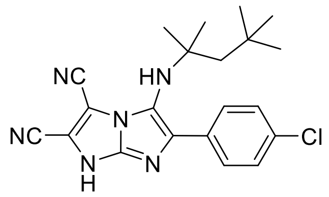

5d

White solid (228 mg, 58%); m.p.: 233-235 °C; IR (neat, cm^{-1}): 3375 (NH), 2945, 2899, 2232 (CN), 1610, 1551, 1186; ^{1}H NMR (400 MHz, DMSO-d_6): δ 13.02 (br s, 1H, NHimidazole), 7.87 (d, J = 8.0 Hz, 2H, HAr), 7.58 (d, J = 8.0 Hz, 2H, HAr), 4.63 (s, 1H, NH), 1.45 (s, 2H, CH_2), 0.97 (s, 6H, $2CH_3$), 0.91 (s, 9H, $3CH_3$); ^{13}C NMR (101 MHz, DMSO-d_6): δ 144.8, 133.9, 130.3, 129.4, 129.1, 128.6, 123.6, 121.3, 114.1, 111.3, 97.8, 59.6, 56.2, 31.9, 31.5, 28.7; HRMS (ESI): (m/z) calcd for $C_{21}H_{24}ClN_6$ [M + H]$^+$: 395.1746, found: 395.1745, (m/z) calcd for $C_{21}H_{23}ClN_6Na$ [M + Na]$^+$: 417.1565, found: 417.1563.

6-(4-(Trifluoromethyl)phenyl)-5-((2,4,4-trimethylpentan-2-yl)amino)-1*H*-imidazo[1,2-*a*]imidazole-2,3-dicarbonitrile (5e)

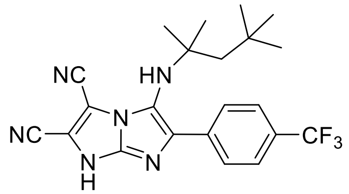

5e

White solid (327 mg, 76%); m.p.: 256-258 °C; IR (neat, cm^{-1}): 3302 (NH), 2955, 2904, 2218 (CN), 1616, 1480, 1331; ^{1}H NMR (400 MHz, DMSO-d_6): δ 13.16 (br s, 1H, NHimidazole), 8.09 (d, J = 8.1 Hz, 2H, HAr), 7.87 (d, J = 8.1 Hz, 2H, HAr), 4.73 (s, 1H, NH), 1.44 (s, 2H, CH_2), 0.98 (s, 6H, $2CH_3$), 0.89 (s, 9H, $3CH_3$); ^{13}C NMR (101 MHz, DMSO-d_6): δ 145.0, 133.8, 129.8, 129.5 (q, J = 32.0 Hz), 129.0, 129.1, 125.8 (q, J = 3.7 Hz), 123.9, 123.1 (q, J = 272.1 Hz), 122.1, 113.9, 111.2, 97.9, 70.2, 59.8, 56.2, 31.8, 31.4, 28.6; ^{19}F NMR (376 MHz, DMSO-d_6): δ -61.3 (s); HRMS (ESI): (m/z) calcd for $C_{22}H_{24}F_3N_6$ [M + H]$^+$: 429.2009, found: 429.2007, (m/z) calcd for $C_{22}H_{23}F_3N_6Na$ [M + Na]$^+$: 451.1829, found: 451.1824.

6-Ethyl-5-((2,4,4-trimethylpentan-2-yl)amino)-1*H*-imidazo[1,2-*a*]imidazole-2,3-dicarbonitrile (5f)

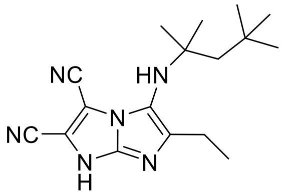

5f

White solid (228 mg, 58%); m.p.: 160-161 °C; IR (neat, cm^{-1}): 3361 (NH), 2958, 2892, 2249 (CN), 1194; ^{1}H NMR (400 MHz, DMSO-d_6): δ 12.45 (br s, 1H, NHimidazole), 4.29 (s, 1H, NH), 2.63 (q, *J* = 7.5 Hz, 2H, CH_2(Et)), 1.59 (s, 2H, CH_2octyl), 1.21 (t, *J* = 7.5 Hz, 3H, CH_3(Et)), 1.14 (s, 6H, 2CH_3), 1.01 (s, 9H, 3CH_3); ^{13}C NMR (101 MHz, DMSO-d_6): δ 144.7, 132.6, 122.8, 120.4, 114.2, 111.5, 97.2, 58.2, 55.7, 32.0, 31.7, 28.7, 18.4, 13.5; HRMS (ESI): (m/z) calcd for $C_{17}H_{25}N_6$ $[M + H]^+$: 313.2135, found: 313.2137, (m/z) calcd for $C_{17}H_{24}N_6Na$ $[M + Na]^+$: 335.1955, found: 335.1958.

6-(Pyridin-3-yl)-5-((2,4,4-trimethylpentan-2-yl)amino)-1*H*-imidazo[1,2-*a*]imidazole-2,3-dicarbonitrile (5g)

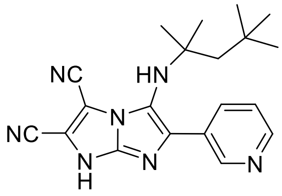

5g

White solid (285 mg, 79%); m.p.: 226-228 °C; IR (neat, cm^{-1}): 3379 (NH), 2955, 2908, 2213 (CN), 1616, 1565, 1383, 1188; ^{1}H NMR (400 MHz, DMSO-d_6): δ 13.13 (br s, 1H, NHimidazole), 9.05 (d, *J* = 1.5 Hz, 1H, HAr), 8.61 (dd, *J* = 4.9, 1.6 Hz, 1H, HAr), 8.22 (dt, *J* = 8.0, 1.9 Hz, 1H, HAr), 7.54 (dd, *J* = 7.5, 4.8 Hz, 1H, HAr), 4.74 (s, 1H, NH), 1.44 (s, 2H, CH_2), 0.97 (s, 6H, 2CH_3), 0.89 (s, 9H, 3CH_3); ^{13}C NMR (101 MHz, DMSO-d_6): δ 150.0, 149.1, 145.1, 135.8, 127.8, 126.0, 123.9, 123.7, 121.8, 114.0, 111.2, 97.8, 59.6, 56.2, 31.8, 31.4, 28.6; HRMS (ESI): (m/z) calcd for $C_{21}H_{25}N_6$ $[M + H]^+$: 362.2088, found: 362.2089.

5-(*tert*-Butylamino)-6-(4-chlorophenyl)-1*H*-imidazo[1,2-*a*]imidazole-2,3-dicarbonitrile (5h)

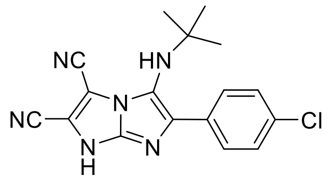

5h

White solid (202 mg, 60%); m.p.: 258-260 °C; IR (neat, cm^{-1}): 3354 (NH), 2942, 2906, 2228 (CN), 1609, 1547, 1180; ^1H NMR (400 MHz, DMSO-d_6): δ 13.01 (br s, 1H, NHimidazole), 7.90 (d, *J* = 8.6 Hz, 2H, HAr), 7.57 (d, *J* = 8.5 Hz, 2H, HAr), 4.84 (s, 1H, NH), 0.98 (s, 9H, $3CH_3$); ^{13}C NMR (101 MHz, DMSO-d_6): δ 144.8, 133.9, 129.9, 129.1, 129.2, 128.3, 123.5, 121.6, 114.0, 111.1, 97.6, 55.5, 30.0; HRMS (ESI): (m/z) calcd for $C_{17}H_{16}ClN_6$ $[M + H]^+$: 339.1120, found: 339.1122, (m/z) calcd for $C_{17}H_{15}ClN_6Na$ $[M + Na]^+$: 361.0939, found: 361.0942.

6-(4-Chlorophenyl)-5-(cyclohexylamino)-1*H*-imidazo[1,2-*a*]imidazole-2,3-dicarbonitrile (5i)

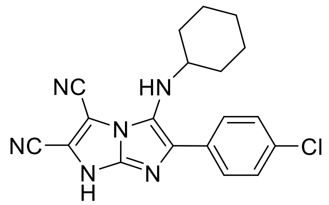

5i

Yellow solid (170 mg, 47%); m.p.: 266-268 °C; IR (neat, cm^{-1}): 3321 (NH), 2930, 2850, 1609, 1570, 1426, 1281, 1224, 1110, 1017; ^1H NMR (400 MHz, DMSO-d_6): δ 12.92 (s, 1H, NHimidazole), 7.94 (d, *J* = 8.3 Hz, 2H, HAr), 7.57 (t, *J* = 8.5 Hz, 2H, HAr), 4.94 (d, *J* = 5.1 Hz, 1H, NH), 3.09–2.83 (m, 1H, CH), 1.84–1.73 (m, 2H, CH_2), 1.62 (dd, *J* = 9.9, 3.9 Hz, 2H, CH_2), 1.49 (d, *J* = 7.4 Hz, 1H, ½CH_2), 1.21–1.04 (m, 5H, (2+½)CH_2); ^{13}C NMR (101 MHz, DMSO-d_6): δ 144.8, 133.4, 129.2, 128.5, 128.0, 126.3, 123.4, 123.1, 114.0, 111.0, 97.6, 56.6, 33.2, 25.7, 24.8; HRMS (ESI): (m/z) calcd for $C_{19}H_{18}ClN_6$ $[M + H]^+$: 365.1276, found: 365.1276.

General procedure for the synthesis of compounds 7a-i

In an open vessel containing a solution of 2-aminoimidazole **3** (1.0 mmol) in EtOH (10 mL), aldehyde (1.1 mmol) and $InCl_3$ (0.02 mmol) were sequentially added, then the mixture was refluxed at 90 °C. After consumption of the starting material (TLC control), the solvent was removed under reduced pressure, then 2 mL of *n*-butanol were added, followed by $ZrCl_4$ (0.10 mmol) and isocyanide (1.1 mmol). The mixture was poured in a closed vessel and heated under microwave irradiation at 140 °C for 10 minutes. After cooling to room temperature, the solvent was evaporated and the crude material was extracted with ethyl acetate (15 mL), washed with water and brine dried over anhydrous Na_2SO_4, filtered and concentrated under reduced pressure. The residue was purified by flash chromatography on silica gel using petroleum ether/ethyl acetate mixture (4:1 v/v) as eluent, or using dichloromethane/methanol mixture (95:5 v/v) as eluent in the case of **7g** to provide the expected pure products **7a-i**.

2-(4-Methoxyphenyl)-*N*-(2,4,4-trimethylpentan-2-yl)-3*H*-imidazo[1,2-*a*]imidazol-3-imine (7a)

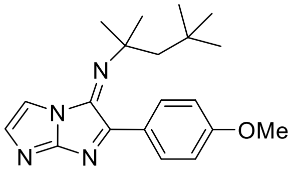

7a

Orange oil (107 mg, 32%); IR (neat, cm^{-1}): 3076, 2958, 2887, 1603, 1572, 1250, 1202, 1121; ^1H NMR (400 MHz, $CDCl_3$): δ 8.48 (d, J = 8.9 Hz, 2H, HAr), 7.18 (d, J = 1.8 Hz, 1H, Himidazole), 7.04 (d, J = 1.7 Hz, 1H, Himidazole), 6.93 (d, J = 8.9 Hz, 2H, HAr), 3.87 (s, 3H, OCH_3), 1.88 (s, 2H, CH_2), 1.55 (s, 6H, $2CH_3$), 0.98 (s, 9H, $3CH_3$); ^{13}C NMR (101 MHz, $CDCl_3$): δ 168.9, 164.2, 162.9, 138.2, 133.5, 132.6, 123.9, 116.2, 113.8, 60.2, 55.4, 53.1, 32.0, 31.7, 28.4; HRMS (ESI): (m/z) calcd for $C_{20}H_{27}N_4O$ $[M + H]^+$: 339.2124, found: 339.2122.

2-Phenyl-*N*-(2,4,4-trimethylpentan-2-yl)-3*H*-imidazo[1,2-*a*]imidazol-3-imine (7b)

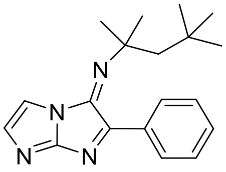

7b

Yellow oil (73 mg, 24%); IR (neat, cm^{-1}): 3081, 2955, 2896, 1606, 1569, 1268, 1209; ^{1}H NMR (400 MHz, $CDCl_3$): δ 8.44 (d, *J* = 7.3 Hz, 2H, HAr), 7.56–7.48 (m, 1H, HAr), 7.44 (t, *J* = 7.6 Hz, 2H, HAr), 7.22 (d, *J* = 1.8 Hz, 1H, Himidazole), 7.09 (d, *J* = 1.7 Hz, 1H, Himidazole), 1.89 (s, 2H, CH_2), 1.56 (s, 6H, $2CH_3$), 0.99 (s, 9H, $3CH_3$); ^{13}C NMR (101 MHz, $CDCl_3$): δ 169.6, 163.9, 137.8, 133.8, 131.9, 131.1, 130.6, 128.2, 116.4, 60.3, 53.2, 32.0, 31.7, 28.3; HRMS (ESI): (m/z) calcd for $C_{19}H_{25}N_4$ $[M + H]^+$: 309.2074, found: 309.2073.

2-(2,4,6-Trimethoxyphenyl)-*N*-(2,4,4-trimethylpentan-2-yl)-3*H*-imidazo[1,2-*a*]imidazol-3-imine (7c)

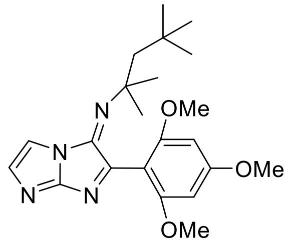

7c

Yellow oil (141 mg, 35%); IR (neat, cm^{-1}): 3069, 2954, 2898, 1609, 1557, 1263, 1211, 1125; ^{1}H NMR (400 MHz, $CDCl_3$): δ 7.18 (d, *J* = 1.8 Hz, 1H, Himidazole), 7.03 (d, *J* = 1.7 Hz, 1H, Himidazole), 6.10 (s, 2H, HAr), 3.82 (s, 3H, OCH_3), 3.69 (s, 6H, $2OCH_3$), 1.63 (s, 2H, CH_2), 1.39 (s, 6H, $2CH_3$), 0.81 (s, 9H, $3CH_3$) ; ^{13}C NMR (101 MHz, $CDCl_3$): δ 172.3, 164.6, 163.0, 159.9, 137.8, 132.8, 116.4, 102.8, 90.5, 59.0, 55.8, 55.4, 55.1, 31.8, 31.3, 27.8; HRMS (ESI): (m/z) calcd for $C_{22}H_{31}N_4O_3$ $[M + H]^+$: 399.2391, found: 399.2388.

2-(4-Chlorophenyl)-*N*-(2,4,4-trimethylpentan-2-yl)-3*H*-imidazo[1,2-*a*]imidazol-3-imine (7d)

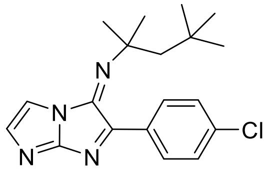

7d

Yellow oil (66 mg, 19%); IR (neat, cm^{-1}): 3085, 2963, 2896, 1601, 1578, 1249, 1202; ^{1}H NMR (400 MHz, $CDCl_3$): δ 8.42 (d, *J* = 8.2 Hz, 2H, HAr), 7.41 (d, *J* = 8.1 Hz, 2H, HAr), 7.21 (s, 1H, Himidazole), 7.09 (s, 1H, Himidazole), 1.88 (s, 2H, CH_2), 1.55 (s, 6H, $2CH_3$), 0.98 (s, 9H, $3CH_3$); ^{13}C NMR (101 MHz, $CDCl_3$): δ 168.4, 163.7, 138.5, 137.8, 134.0, 131.8, 129.5, 128.6, 116.5, 60.4, 53.2, 32.0, 31.7, 28.3; HRMS (ESI): (m/z) calcd for $C_{19}H_{24}ClN_4$ $[M + H]^+$: 343.1684, found: 343.1687.

2-(4-(Trifluoromethyl)phenyl)-*N*-(2,4,4-trimethylpentan-2-yl)-3*H*-imidazo[1,2-*a*]imidazol-3-imine (7e)

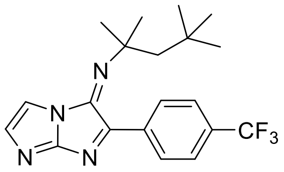

7e

Orange solid (50 mg, 13%); m.p.: 106-107 °C; IR (neat, cm^{-1}): 3080, 2971, 2893, 1608, 1553, 1236, 1192; ^{1}H NMR (400 MHz, $CDCl_3$): δ 8.57 (d, *J* = 8.1 Hz, 2H, HAr), 7.70 (d, *J* = 8.1 Hz, 2H, HAr), 7.24 (d, *J* = 1.8 Hz, 1H, Himidazole), 7.13 (d, *J* = 1.8 Hz, 1H, Himidazole), 1.90 (s, 2H, CH_2), 1.57 (s, 6H, $2CH_3$), 0.99 (s, 9H, $3CH_3$); ^{13}C NMR (101 MHz, $CDCl_3$): δ 168.0, 163.4, 137.6, 134.2, 134.1, 132.9 (q, *J* = 32.5 Hz), 130.7, 125.1 (q, *J* = 3.8 Hz), 122.4 (q, *J* = 272.3 Hz), 116.7, 60.6, 53.1, 32.0, 31.7, 28.3; ^{19}F NMR (376 MHz, $CDCl_3$): δ -61.1 (s); HRMS (ESI): (m/z) calcd for $C_{20}H_{24}F_3N_4$ $[M + H]^+$: 377.1948, found: 377.1944, (m/z) calcd for $C_{20}H_{23}F_3N_4Na$ $[M + Na]^+$: 399.1767, found: 399.1767.

2-Ethyl-*N*-(2,4,4-trimethylpentan-2-yl)-3*H*-imidazo[1,2-*a*]imidazol-3-imine (7f)

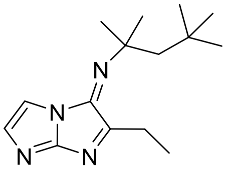

7f

Yellow oil (41 mg, 16%); IR (neat, cm^{-1}): 3083, 2977, 2889, 1602, 1560, 1245; ^1H NMR (400 MHz, $CDCl_3$): δ 7.12 (d, *J* = 1.8 Hz, 1H, Himidazole), 7.00 (d, *J* = 1.8 Hz, 1H, Himidazole), 2.74 (q, *J* = 7.4 Hz, 2H, CH_2(Et)), 1.77 (s, 2H, CH_2octyl), 1.45 (s, 6H, $2CH_3$), 1.30 (t, *J* = 7.4 Hz, 3H, CH_3(Et)), 1.00 (s, 9H, $3CH_3$); ; ^{13}C NMR (101 MHz, $CDCl_3$): δ 180.7, 164.6, 137.9, 133.0, 116.2, 59.3, 54.7, 32.0, 31.7, 27.8, 22.7, 10.6; HRMS (ESI): (m/z) calcd for $C_{15}H_{25}N_4$ $[M + H]^+$: 261.2074, found: 261.2073.

2-(Pyridin-3-yl)-*N*-(2,4,4-trimethylpentan-2-yl)-3*H*-imidazo[1,2-*a*]imidazol-3-imine (7g)

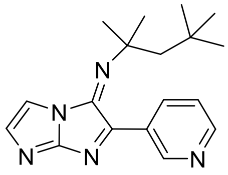

7g

Orange oil (38 mg, 12%); IR (neat, cm^{-1}): 3089, 2943, 2905, 1612, 1560, 1257, 1202; ^1H NMR (400 MHz, $CDCl_3$): δ 9.63 (s, 1H, HAr), 8.72 (dd, *J* = 4.9, 1.7 Hz, 1H, HAr), 8.66 (dt, *J* = 8.1, 2.0 Hz, 1H, HAr), 7.40 (ddd, *J* = 8.0, 4.8, 0.9 Hz, 1H, HAr), 7.24 (d, *J* = 1.8 Hz, 1H, Himidazole), 7.12 (d, *J* = 1.7 Hz, 1H, Himidazole), 1.89 (s, 2H, CH_2), 1.57 (s, 6H, $2CH_3$), 0.98 (s, 9H, $3CH_3$); ^{13}C NMR (101 MHz, $CDCl_3$): δ 167.9, 163.7, 152.1, 151.3, 137.5, 137.2, 134.1, 127.1, 123.2, 116.7, 60.6, 53.2, 32.0, 31.7, 28.4; HRMS (ESI): (m/z) calcd for $C_{18}H_{24}N_5$ $[M + H]^+$: 310.2026, found: 310.2027, (m/z) calcd for $C_{18}H_{23}N_5Na$ $[M + Na]^+$: 332.1846, found: 332.1848.

***N*-(*tert*-Butyl)-2-(4-methoxyphenyl)-3*H*-imidazo[1,2-*a*]imidazol-3-imine (7h)**

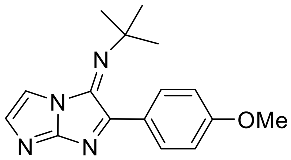

7h

Orange oil (47 mg, 17%); IR (neat, cm^{-1}): 3088, 2945, 2890, 1606, 1542, 1256, 1211, 1120; ^1H NMR (400 MHz, $CDCl_3$): δ 8.50 (d, *J* = 9.0 Hz, 2H, HAr), 7.15 (d, *J* = 1.8 Hz, 1H, Himidazole), 7.04 (d, *J* = 1.8 Hz, 1H, Himidazole), 6.94 (d, *J* = 9.0 Hz, 2H, HAr), 3.87 (s, 3H, OCH_3), 1.51 (s, 9H, $3CH_3$); ^{13}C

NMR (101 MHz, $CDCl_3$): δ 169.1, 164.1, 163.0, 139.3, 133.6, 132.5, 123.8, 116.3, 113.9, 56.1, 55.4, 28.2; HRMS (ESI): (m/z) calcd for $C_{16}H_{19}N_4O$ $[M + H]^+$: 283.1553, found: 283.1553, (m/z) calcd for $C_{16}H_{18}N_4NaO$ $[M + Na]^+$: 305.1373, found: 305.1374.

_N_-Cyclohexyl-2-(4-methoxyphenyl)-3_H_-imidazo[1,2-_a_]imidazol-3-imine (7i)

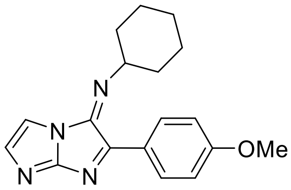

7i

Orange oil (30 mg, 10%); IR (neat, cm^{-1}): 3082, 2951, 2884, 1605, 1562, 1248, 1224, 1129; ^1H NMR (400 MHz, $CDCl_3$): δ 8.55 (d, J = 9.0 Hz, 2H, HAr), 7.04 (dd, J = 5.0, 1.8 Hz, 2H, Himidazole), 6.96 (d, J = 9.0 Hz, 2H, HAr), 3.88 (s, 3H, OCH_3), 3.82–3.76 (m, 1H, CH), 1.93–1.88 (m, 3H, 3/2CH_2), 1.73–1.62 (m, 4H, 2CH_2), 1.50–1.39 (m, 3H, 3/2CH_2); ^{13}C NMR (101 MHz, $CDCl_3$): δ 167.9, 163.2, 163.0, 142.6, 133.9, 132.2, 123.7, 115.0, 114.0, 60.4, 55.4, 32.5, 25.5, 24.2; HRMS (ESI): (m/z) calcd for $C_{18}H_{21}N_4O$ $[M + H]^+$: 309.1710, found: 309.1709, (m/z) calcd for $C_{18}H_{20}N_4NaO$ $[M + Na]^+$: 331.1529, found: 331.1529.

General procedure for the synthesis of compounds 8a-d, 9a-g

In a closed vessel containing a mixture of DCM/TFA (5 mL, 1:1 v/v), 0.2 mmol of **4a-d** or **5a-g** was added at room temperature. After completion of the reaction (30 min), the solvents were removed under reduced pressure, and the mixture was partitioned between saturated aqueous solution of $NaHCO_3$ (10 mL) and EtOAc (15 mL). The organic layer was washed with brine, dried over anhydrous Na_2SO_4, filtered and concentrated under reduced pressure. The residue was then purified by flash chromatography on silica gel using petroleum ether/ethyl acetate mixture (1:1 – 1:4 v/v) as eluent to afford the expected pure products **8a-d** and **9a-g.**

Ethyl 5-amino-6-phenyl-1*H*-imidazo[1,2-*a*]imidazole-3-carboxylate (8a)

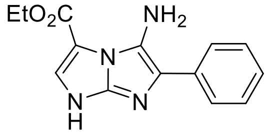

8a

Yellow solid (38 mg, 70%); m.p.: 202-204 °C; IR (neat, cm^{-1}): 3420 (NH), 2981, 2899, 1693 (CO), 1596, 1510, 1247, 1196; ^1H NMR (400 MHz, DMSO-d_6): δ 11.80 (br s, 1H, NHimidazole), 7.71 (s, 1H, Himidazole), 7.66 (d, *J* = 7.2 Hz, 2H, HAr), 7.41 (t, *J* = 7.8 Hz, 2H, HAr), 7.19 (t, *J* = 7.4 Hz, 1H, HAr), 5.95 (s, 2H, NH_2), 4.27 (q, *J* = 7.1 Hz, 2H, CH_2ester), 1.30 (t, *J* = 7.1 Hz, 3H, CH_3ester); ^{13}C NMR (101 MHz, DMSO-d_6): δ 161.3, 147.4, 142.3, 131.4, 129.1, 125.8, 125.7, 124.4, 113.0, 110.1, 60.4, 14.8; HRMS (ESI): (m/z) calcd for $C_{14}H_{15}N_4O_2$ $[M + H]^+$: 271.1189, found: 271.1186.

Ethyl 5-amino-6-(4-methoxyphenyl)-1*H*-imidazo[1,2-*a*]imidazole-3-carboxylate (8b)

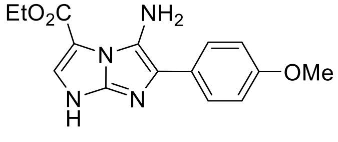

8b

Brown solid (44 mg, 74%); m.p.: 104-106 °C; IR (neat, cm^{-1}): 3379 (NH), 2975, 2902, 1698 (CO), 1601, 1502, 1258, 1108; ^1H NMR (250 MHz, DMSO-d_6): δ 11.72 (br s, 1H, NHimidazole), 7.67 (s, 1H, Himidazole), 7.59 (d, *J* = 8.8 Hz, 2H, HAr), 7.00 (d, *J* = 8.8 Hz, 2H, HAr), 5.72 (s, 2H, NH_2), 4.26

(q, J = 7.1 Hz, 2H, CH_2ester), 3.78 (s, 3H, OCH_3), 1.29 (t, J = 7.1 Hz, 3H, CH_3ester); ^{13}C NMR (62.9 MHz, DMSO-d_6): δ 161.2, 157.8, 146.0, 140.6, 126.2, 124.5, 123.7, 114.7, 113.2, 110.8, 60.6, 55.6, 14.8; HRMS (ESI): (m/z) calcd for $C_{15}H_{17}N_4O_3$ $[M + H]^+$: 301.1295, found: 301.1301.

Ethyl 5-amino-6-(4-chlorophenyl)-1*H*-imidazo[1,2-*a*]imidazole-3-carboxylate (8c)

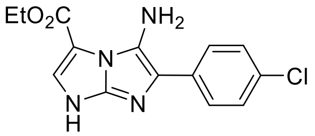

8c

Yellow solid (41 mg, 68%); m.p.: 212-214 °C; IR (neat, cm^{-1}): 3393 (NH), 2989, 2909, 1691 (CO), 1612, 1508, 1270; ^1H NMR (400 MHz, DMSO-d_6): δ 11.86 (br s, 1H, NHimidazole), 7.70 (s, 1H, Himidazole), 7.66 (d, J = 8.4 Hz, 2H, HAr), 7.00 (d, J = 8.3 Hz, 2H, HAr), 6.01 (s, 2H, NH_2), 4.27 (q, J = 7.1 Hz, 2H, CH_2ester), 1.30 (t, J = 7.1 Hz, 3H, CH_3ester); ^{13}C NMR (100.1 MHz, DMSO-d_6): δ 161.3, 147.4, 142.3, 130.4, 129.8, 129.0, 126.3, 125.9, 113.1, 109.4, 60.5, 14.8; HRMS (ESI): (m/z) calcd for $C_{14}H_{14}ClN_4O_2$ $[M + H]^+$: 305.0800, found: 305.0796.

Ethyl 5-amino-6-(4-(trifluoromethyl)phenyl)-1*H*-imidazo[1,2-*a*]imidazole-3-carboxylate (8d)

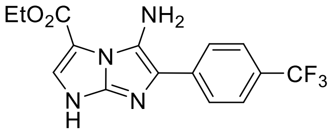

8d

Yellow solid (44 mg, 65%); m.p.: 223-225 °C; IR (neat, cm^{-1}): 3382 (NH), 2990, 2895, 1698 (CO), 1610, 1488, 1254; ^1H NMR (400 MHz, DMSO-d_6): δ 11.86 (br s, 1H, NHimidazole), 7.70 (s, 1H, Himidazole), 7.66 (d, J = 8.4 Hz, 2H, HAr), 7.00 (d, J = 8.3 Hz, 2H, HAr), 6.01 (s, 2H, NH_2), 4.27 (q, J = 7.1 Hz, 2H, CH_2ester), 1.30 (t, J = 7.1 Hz, 3H, CH_3ester); ^{13}C NMR (100.1 MHz, DMSO-d_6): δ 161.3, 147.6, 142.6, 135.6, 127.8, 125.9 (q, J = 3.9 Hz), 125.0 (q, J = 32.0 Hz), 124.3, 123.6 (q, J = 271.4 Hz), 113.2, 108.9, 60.6, 14.8; ^{19}F NMR (376 MHz, $CDCl_3$): δ -60.7 (s); HRMS (ESI): (m/z) calcd for $C_{15}H_{14}F_3N_4O_2$ $[M + H]^+$: 339.1063, found: 339.1064.

5-Amino-6-phenyl-1*H*-imidazo[1,2-*a*]imidazole-2,3-dicarbonitrile (9a)

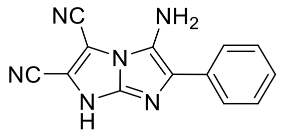

9a

Yellow solid (29 mg, 59%); m.p.: 260-262 °C; IR (neat, cm^{-1}): 3396 (NH), 2954, 2909, 2214 (CN), 1627, 1567, 1389; ^{1}H NMR (400 MHz, DMSO-d_6): δ 12.66 (s, 1H, NHimidazole), 7.79 (d, *J* = 7.2 Hz, 2H, HAr), 7.49 (t, *J* = 7.7 Hz, 2H, HAr), 7.37 (t, *J* = 7.4 Hz, 1H, HAr), 4.90 (s, 2H, NH_2); ^{13}C NMR (101 MHz, DMSO-d_6): δ 144.3, 129.4, 129.3, 128.2, 126.6, 123.1, 122.8, 121.8, 114.2, 110.9, 96.9; HRMS (ESI): (m/z) calcd for $C_{13}H_9N_6$ $[M + H]^+$: 249.0883, found: 249.0881, (m/z) calcd for $C_{13}H_8N_6Na$ $[M + Na]^+$: 271.0703, found: 271.0701.

5-Amino-6-(4-methoxyphenyl)-1*H*-imidazo[1,2-*a*]imidazole-2,3-dicarbonitrile (9b)

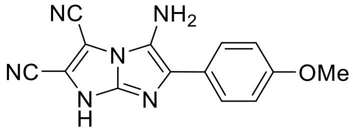

9b

Yellow solid (34 mg, 61%); m.p.: 227-229 °C; IR (neat, cm^{-1}): 3403 (NH), 2949, 2912, 2225 (CN), 1618, 1560, 1398, 1134; ^{1}H NMR (400 MHz, DMSO-d_6): δ 12.57 (s, 1H, NHimidazole), 7.74 (d, *J* = 8.5 Hz, 2H, HAr), 7.06 (d, *J* = 8.5 Hz, 2H, HAr), 4.73 (s, 2H, NH_2), 3.81 (s, 3H, OCH_3); ^{13}C NMR (101 MHz, DMSO-d_6): δ 159.4, 144.1, 128.2, 122.8, 122.7, 121.7, 121.5, 114.7, 114.2, 110.9, 96.8, 55.7; HRMS (ESI): (m/z) calcd for $C_{14}H_{10}N_6O$ $[M + H]^+$: 279.0989, found: 279.0988.

5-Amino-6-(2,4,6-trimethoxyphenyl)-1*H*-imidazo[1,2-*a*]imidazole-2,3-dicarbonitrile (9c)

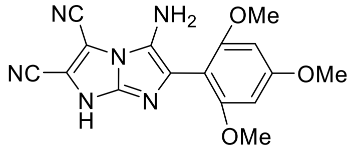

9c

Yellow solid (22 mg, 32%); m.p.: 262-264 °C; IR (neat, cm^{-1}): 3392 (NH), 2964, 2906, 2217 (CN), 1611, 1552, 1420, 1102; ^{1}H NMR (400 MHz, DMSO-d_6): δ 12.04 (s, 1H, NHimidazole), 6.35 (s, 2H,

HAr), 4.31 (s, 2H, NH_2), 3.85 (s, 3H, OCH_3), 3.77 (s, 6H, $2OCH_3$); ^{13}C NMR (101 MHz, DMSO-d_6): δ 162.8, 159.6, 144.0, 124.4, 122.4, 114.4, 113.3, 111.2, 98.2, 95.9, 91.3, 56.3, 56.0; HRMS (ESI): (m/z) calcd for $C_{16}H_{15}N_6O_3$ $[M + H]^+$: 339.1200, found: 339.1200, (m/z) calcd for $C_{16}H_{14}N_6NaO_3$ $[M + Na]^+$: 361.1020, found: 361.1019.

5-Amino-6-(4-chlorophenyl)-1*H*-imidazo[1,2-*a*]imidazole-2,3-dicarbonitrile (9d)

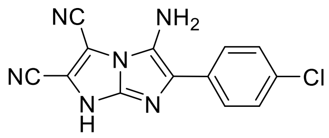

9d

Yellow solid (34 mg, 57%); m.p.: 268-270 °C; IR (neat, cm^{-1}): 3403 (NH), 2949, 2912, 2225 (CN), 1618, 1560, 1398, 1134; ^{1}H NMR (400 MHz, DMSO-d_6): δ 12.70 (s, 1H, NHimidazole), 7.81 (d, J = 8.7 Hz, 2H, HAr), 7.56 (d, J = 8.6 Hz, 2H, HAr), 4.95 (s, 2H, NH_2); ^{13}C NMR (101 MHz, DMSO-d_6): δ 144.4, 132.7, 129.3, 128.3, 128.2, 123.3, 123.2, 120.8, 114.1, 110.8, 97.0; HRMS (ESI): (m/z) calcd for $C_{13}H_8ClN_6$ $[M + H]^+$: 283.0493, found: 283.0492.

5-Amino-6-(4-(trifluoromethyl)phenyl)-1*H*-imidazo[1,2-*a*]imidazole-2,3-dicarbonitrile (9e)

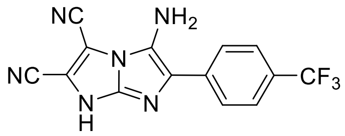

9e

Yellow solid (50 mg, 79%); m.p.: 247-249 °C; IR (neat, cm^{-1}): 3384 (NH), 2963, 2905, 2221 (CN), 1626, 1545, 1410, 1117; ^{1}H NMR (400 MHz, DMSO-d_6): δ 12.80 (s, 1H, NHimidazole), 7.98 (d, J = 8.2 Hz, 2H, HAr), 7.84 (d, J = 8.2 Hz, 2H, HAr), 5.15 (s, 2H, NH_2); ^{13}C NMR (101 MHz, DMSO-d_6): δ 144.6, 133.5, 127.6 (q, J = 32.0 Hz), 126.8, 126.1 (q, J = 3.9 Hz), 124.6, 123.7, 122.4 (q, J = 271.8 Hz), 119.5, 114.0, 110.7, 97.1; ^{19}F NMR (376 MHz, $CDCl_3$): δ -61.0 (s); HRMS (ESI): (m/z) calcd for $C_{14}H_7F_3N_6$ $[M + H]^+$: 317.0757, found: 317.0758.

5-Amino-6-ethyl-1*H*-imidazo[1,2-*a*]imidazole-2,3-dicarbonitrile (9f)

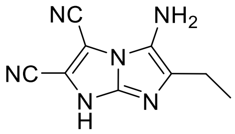

9f

Colorless oil (16 mg, 41%); IR (neat, cm^{-1}): 3403 (NH), 2949, 2912, 2225 (CN), 1618, 1560, 1398, 1134; ^{1}H NMR (400 MHz, DMSO-d_6): δ 12.13 (s, 1H, NHimidazole), 4.45 (s, 2H, NH_2), 2.58 (q, *J* = 7.5 Hz, 2H, CH_2), 1.16 (t, *J* = 7.5 Hz, 3H, CH_3); ^{13}C NMR (101 MHz, DMSO-d_6): δ 173.5, 143.9, 124.4, 121.8, 114.3, 111.0, 96.1, 17.4, 13.8; HRMS (ESI): (m/z) calcd for $C_9H_9N_6$ $[M + H]^+$: 200.0924, found: 200.0921.

5-Amino-6-(pyridin-3-yl)-1*H*-imidazo[1,2-*a*]imidazole-2,3-dicarbonitrile (9g)

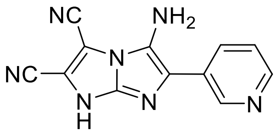

9g

Yellow solid (13 mg, 26%); m.p.: 255-257 °C; IR (neat, cm^{-1}): 3374 (NH), 2966, 2900, 2219 (CN), 1632, 1548, 1365; ^{1}H NMR (400 MHz, DMSO-d_6): δ 12.83 (br s, 1H, NHimidazole), 9.01 (s, 1H, HAr), 8.54 (dd, *J* = 4.9, 1.6 Hz, 1H, HAr), 8.16 (dt, *J* = 8.1, 1.9 Hz, 1H, HAr), 7.52 (dd, *J* = 8.1, 4.8 Hz, 1H, HAr), 5.04 (s, 2H, NH_2); ^{13}C NMR (101 MHz, DMSO-d_6): δ 148.8, 147.4, 144.9, 133.7, 125.9, 124.1, 123.8, 123.4, 119.1, 114.1, 110.8, 96.9; HRMS (ESI): (m/z) calcd for $C_{12}H_8N_7$ $[M + H]^+$: 250.0836, found: 250.0834.

General procedure for the synthesis of compounds 10a, 10b, 10d, 10e, 10g

In a closed vessel containing a mixture of DCM/TFA (5 mL, 4:1 v/v), 0.15 mmol of **7a**, **7b**, **7d**, **7e** or **7g** was added. The solution was stirred at room temperature for 10 min. After completion of the reaction, TFA and DCM were evaporated under reduced pressure. The mixture was then partitioned between saturated aqueous solution of $NaHCO_3$ (10 mL) and EtOAc (15 mL). The organic layer was washed with brine, dried over anhydrous Na_2SO_4, filtered and concentrated under reduced pressure. The residue was purified by flash chromatography on silica gel using petroleum ether/ethyl acetate mixture (3:2 v/v) as eluent, or using dichloromethane/methanol mixture (96:4 v/v) as eluent in the case of **10g** to afford the desired pure products **10a**, **10b**, **10d**, **10e** and **10g**.

2-(4-Methoxyphenyl)-3*H*-imidazo[1,2-*a*]imidazol-3-imine (10a)

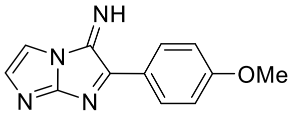

10a

Yellow solid (25 mg, 74%); m.p.: 180-182 °C; IR (neat, cm^{-1}): 3261 (NH), 3084, 2938, 2873, 1592, 1224, 1116; ^1H NMR (400 MHz, $CDCl_3$): δ 12.79 (s, 1H, NH), 8.02 (d, *J* = 8.5 Hz, 2H, HAr), 7.38 (s, 1H, Himidazole), 7.17 (d, *J* = 8.9 Hz, 3H, Himidazole+2HAr), 3.88 (s, 3H, OCH_3); ^{13}C NMR (101 MHz, $CDCl_3$): δ 163.3, 147.5, 131.7, 129.9, 127.2, 120.0, 115.3, 113.2, 56.2; HRMS (ESI): (m/z) calcd for $C_{12}H_{11}N_4O$ $[M + H]^+$: 227.0928, found: 227.0927, (m/z) calcd for $C_{12}H_{10}N_4NaO$ $[M + Na]^+$: 249.0747, found: 249.0746.

2-Phenyl-3*H*-imidazo[1,2-*a*]imidazol-3-imine (10b)

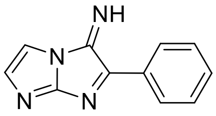

10b

Yellow solid (25 mg, 85%); m.p.: 185-187 °C; IR (neat, cm^{-1}): 3273 (NH), 2934, 2836, 1604, 1510, 1463, 1247; ^1H NMR (400 MHz, $CDCl_3$): δ 12.97 (s, 1H, NH), 8.07 (dd, *J* = 7.9, 1.8 Hz, 2H, HAr), 7.67–7.59 (m, 3H, HAr), 7.35 (s, 2H, Himidazole); ^{13}C NMR (101 MHz, $CDCl_3$): δ 147.2, 134.5, 132.9, 131.6, 129.8, 127.8, 113.2; HRMS (ESI): (m/z) calcd for $C_{11}H_9N_4$ $[M + H]^+$: 197.0822, found: 197.0823, (m/z) calcd for $C_{11}H_8N_4Na$ $[M + Na]^+$: 219.0641, found: 219.0641.

2-(4-Chlorophenyl)-3*H*-imidazo[1,2-*a*]imidazol-3-imine (10d)

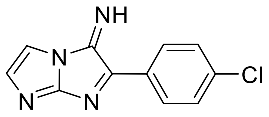

10d

Yellow solid (28 mg, 82%); m.p.: 156-158 °C; IR (neat, cm^{-1}): 3296 (NH), 3080, 2946, 2871, 1564, 1226; ^1H NMR (400 MHz, $CDCl_3$): δ 13.01 (s, 1H, NH), 8.04 (d, *J* = 8.7 Hz, 2H, HAr), 7.68 (d, *J* = 8.7 Hz, 2H, 2HAr), 7.56–7.19 (m, 2H, Himidazole); ^{13}C NMR (101 MHz, $CDCl_3$): δ 147.0, 137.6, 133.5, 130.2, 129.9, 129.4, 113.1; HRMS (ESI): (m/z) calcd for $C_{11}H_8ClN_4$ $[M + H]^+$: 231.0539, found: 231.0537.

2-(4-(Trifluoromethyl)phenyl)-3*H*-imidazo[1,2-*a*]imidazol-3-imine (10e)

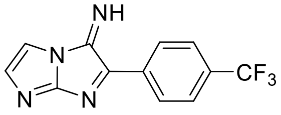

10e

Yellow solid (25 mg, 63%); m.p.: 185-186 °C; IR (neat, cm^{-1}): 3305 (NH), 3079, 2950, 2866, 1553, 1216; ^1H NMR (400 MHz, $CDCl_3$): δ 13.15 (s, 1H, NH), 8.24 (d, *J* = 8.2 Hz, 2H, HAr), 7.68 (d, *J* = 8.3 Hz, 2H, 2HAr), 7.43 (s, 2H, Himidazole); ^{13}C NMR (101 MHz, $CDCl_3$): δ 146.9, 138.2, 138.2, 131.7 (q, *J* = 32.2 Hz), 129.6, 129.3, 128.5, 126.6 (q, *J* = 3.9 Hz), 122.1 (q, *J* = 272.4 Hz), 113.2; ^{19}F NMR (376 MHz, $CDCl_3$): δ -61.5 (s); HRMS (ESI): (m/z) calcd for $C_{12}H_8F_3N_4$ $[M + H]^+$: 265.0696, found: 265.0697, (m/z) calcd for $C_{12}H_7F_3N_4Na$ $[M + Na]^+$: 287.0515, found: 287.0520.

2-(Pyridin-3-yl)-3*H*-imidazo[1,2-*a*]imidazol-3-imine (10g)

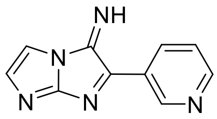

10g

Yellow solid (19 mg, 64%); m.p.: 176-178 °C; IR (neat, cm^{-1}): 3286 (NH), 2977, 2861, 1609, 1523, 1438, 1259; ^{1}H NMR (400 MHz, $CDCl_3$): δ 13.10 (br s, 1H, NH), 9.18 (d, *J* = 1.5 Hz, 1H, , HAr), 8.78 (dd, *J* = 4.8, 1.6 Hz, 1H, HAr), 8.34 (ddd, *J* = 8.1, 2.4, 1.6 Hz, 1H, , HAr), 7.64 (ddd, *J* = 8.1, 4.8, 0.9 Hz, 1H, HAr), 7.39 (s, 2H, Himidazole); ^{13}C NMR (101 MHz, $CDCl_3$): δ 152.8, 148.6, 147.1, 135.2, 130.6, 129.2, 124.6, 112.9; HRMS (ESI): (m/z) calcd for $C_{10}H_8N_5$ $[M + H]^+$: 198.0774, found: 198.0769, (m/z) calcd for $C_{10}H_7N_5Na$ $[M + Na]^+$: 220.0594, found: 220.0588.

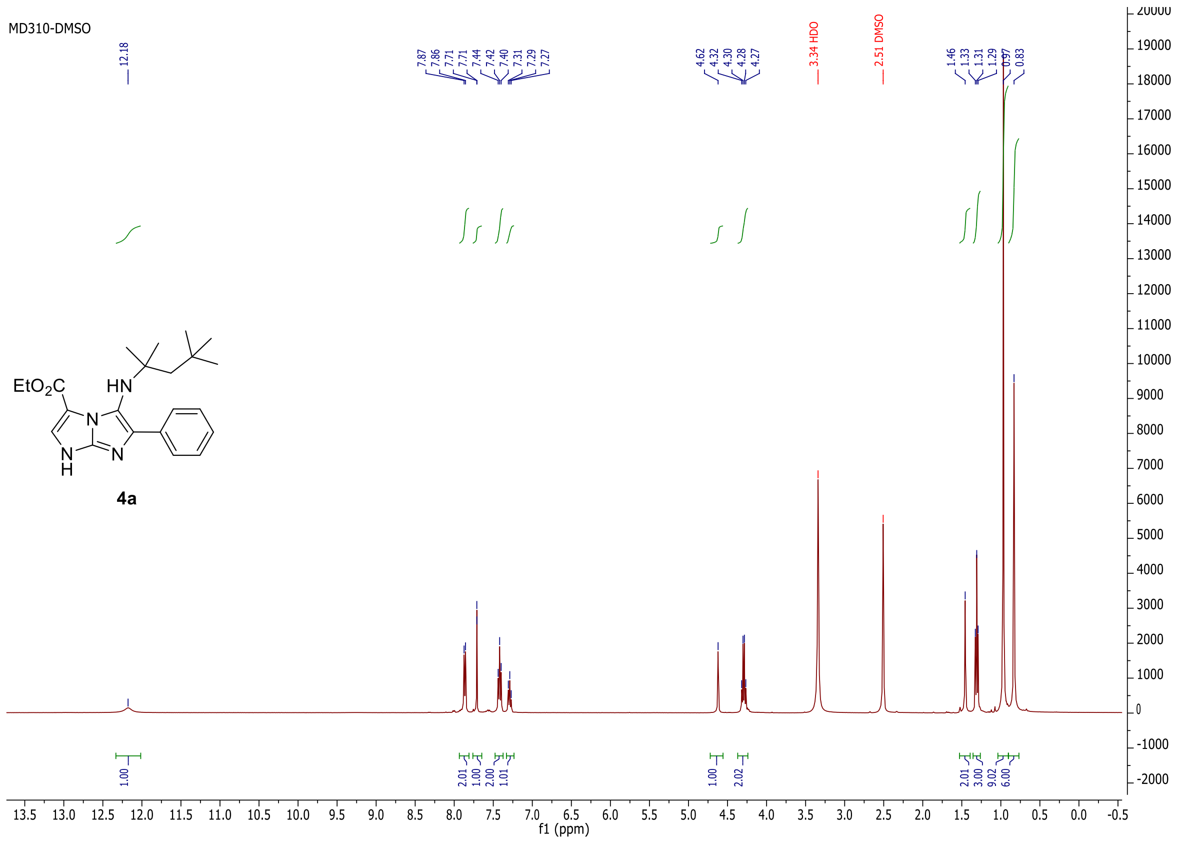
MD310-DMSO
12.18
7.87
7.86
7.71
7.71
7.44
7.42
7.40
7.31
7.29
7.27
4.62
4.32
4.30
4.28
4.27
3.34 HDO
2.51 DMSO
1.46
1.33
1.31
1.29
0.97
0.83
EtO2C
HN
N
N
H
4a
1.00
2.01
1.00
2.00
1.01
1.00
2.02
2.01
3.00
9.02
6.00
f1 (ppm)

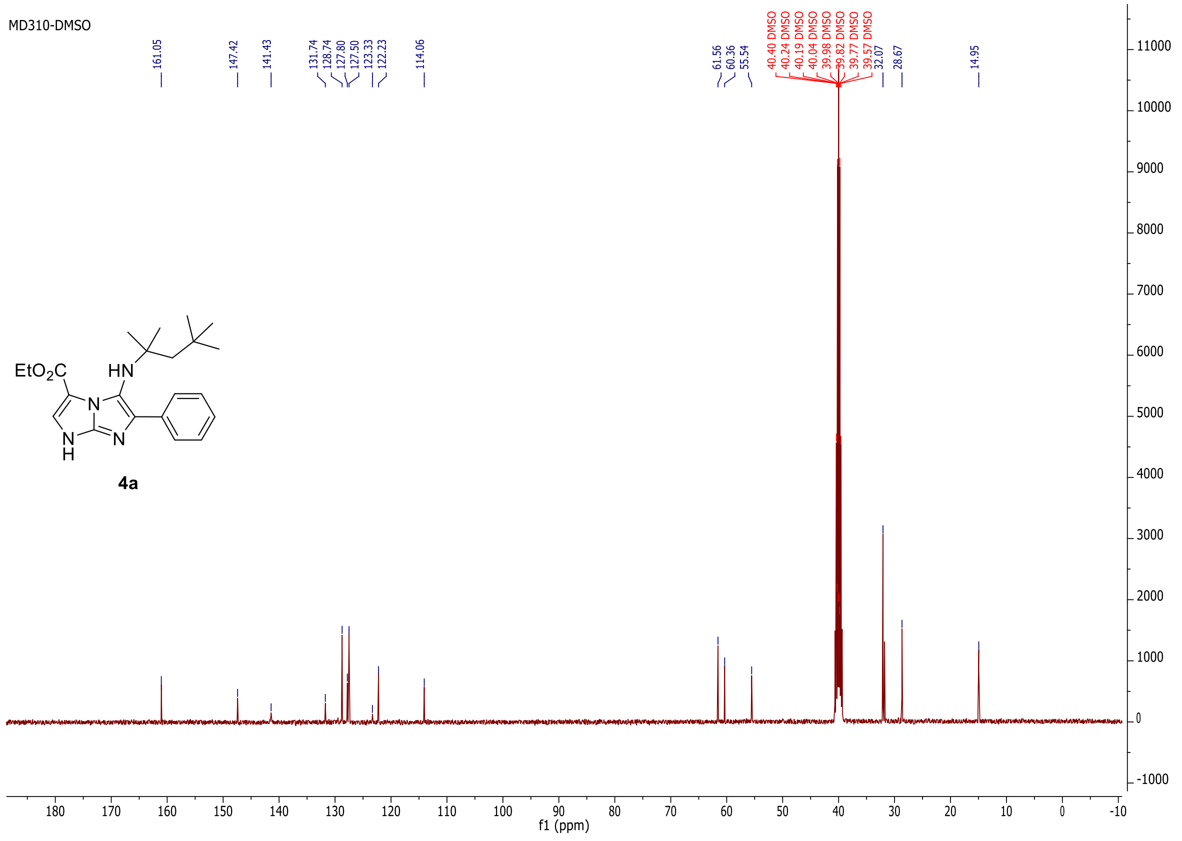
MD310-DMSO
161.05
147.42
141.43
131.74
128.74
127.80
127.50
123.33
122.23
114.06
61.56
60.36
55.54
40.40 DMSO
40.24 DMSO
40.19 DMSO
40.04 DMSO
39.98 DMSO
39.82 DMSO
39.77 DMSO
39.57 DMSO
32.07
28.67
14.95
EtO2C
HN
N
N
H
4a
f1 (ppm)

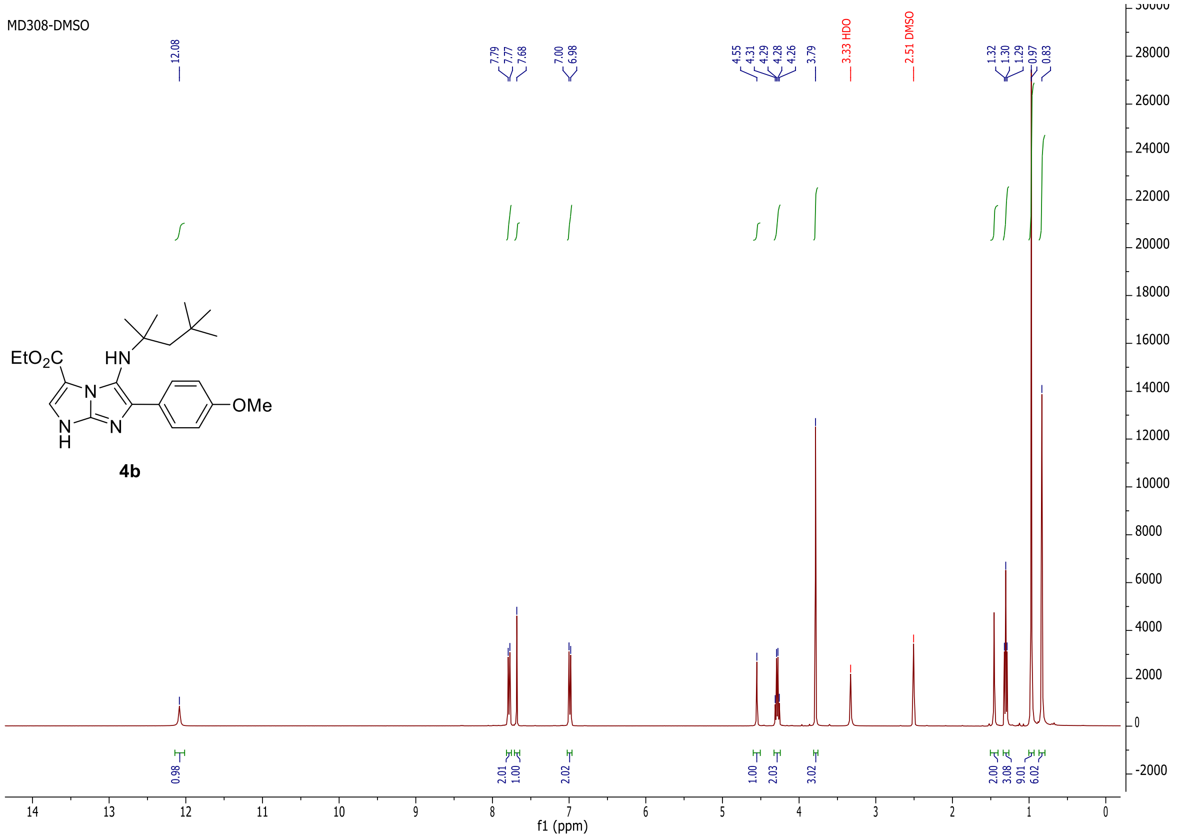

MD308-DMSO
12.08
7.79
7.77
7.68
7.00
6.98
4.55
4.31
4.29
4.28
4.26
3.79
3.33 HDO
2.51 DMSO
1.32
1.30
1.29
0.97
0.83
EtO2C
HN
N
N
H
OMe
4b
0.98
2.01
1.00
2.02
1.00
2.03
3.02
2.00
3.08
9.01
6.02
f1 (ppm)

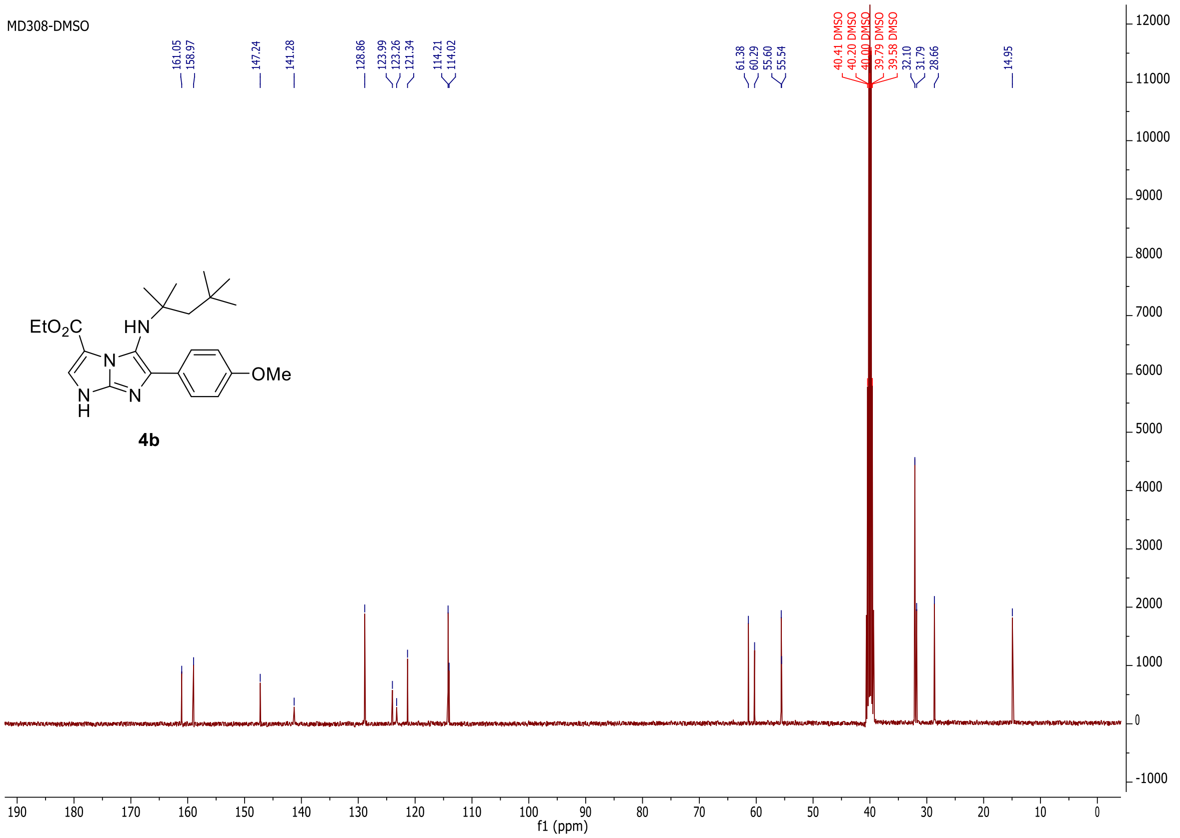

MD308-DMSO
161.05
158.97
147.24
141.28
128.86
123.99
123.26
121.34
114.21
114.02
61.38
60.29
55.60
55.54
40.41 DMSO
40.20 DMSO
40.00 DMSO
39.79 DMSO
39.58 DMSO
32.10
31.79
28.66
14.95
EtO2C
HN
N
N
H
OMe
4b
f1 (ppm)

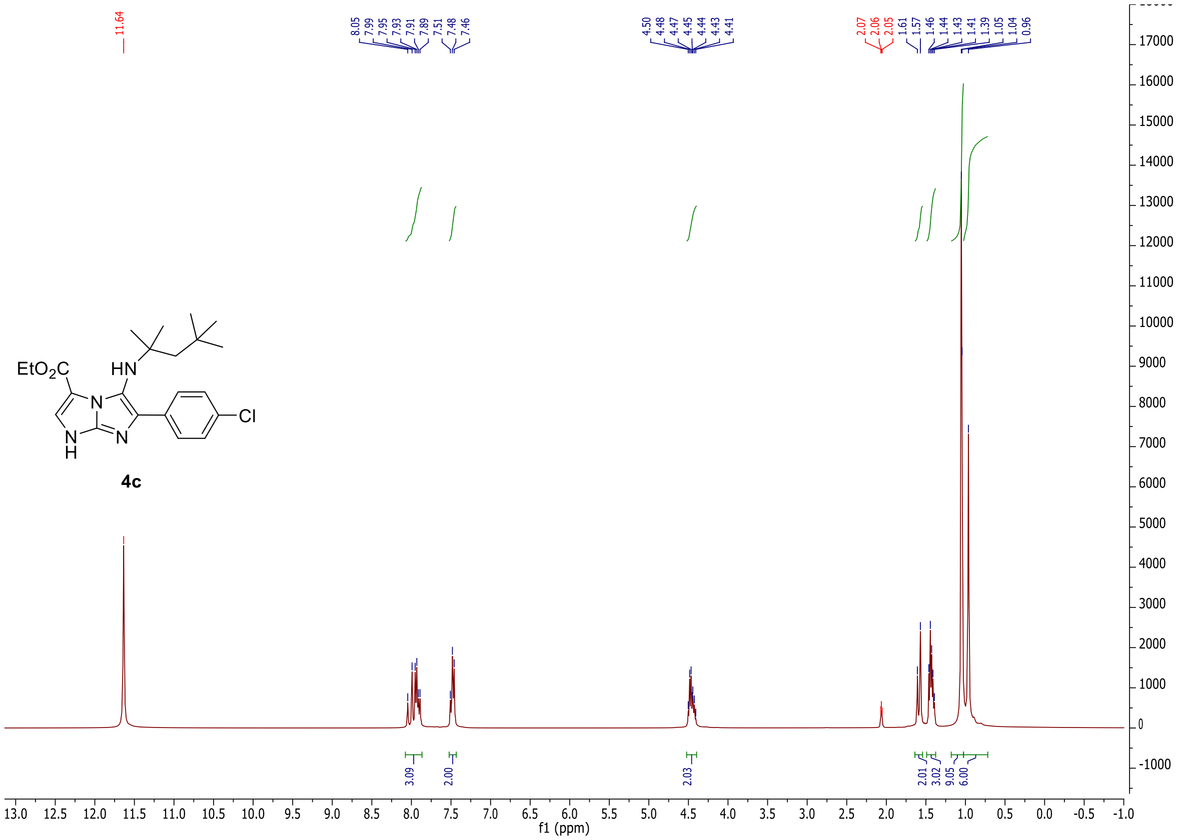
11.64
8.05 7.99 7.95 7.93 7.91 7.89 7.51 7.48 7.46
4.50 4.48 4.47 4.45 4.44 4.43 4.41
2.07 2.06 2.05 1.61 1.57 1.46 1.44 1.43 1.41 1.39 1.05 1.04 0.96
EtO2C
HN
N
N
H
Cl
4c
3.09
2.00
2.03
2.01
3.02
9.05
6.00
f1 (ppm)

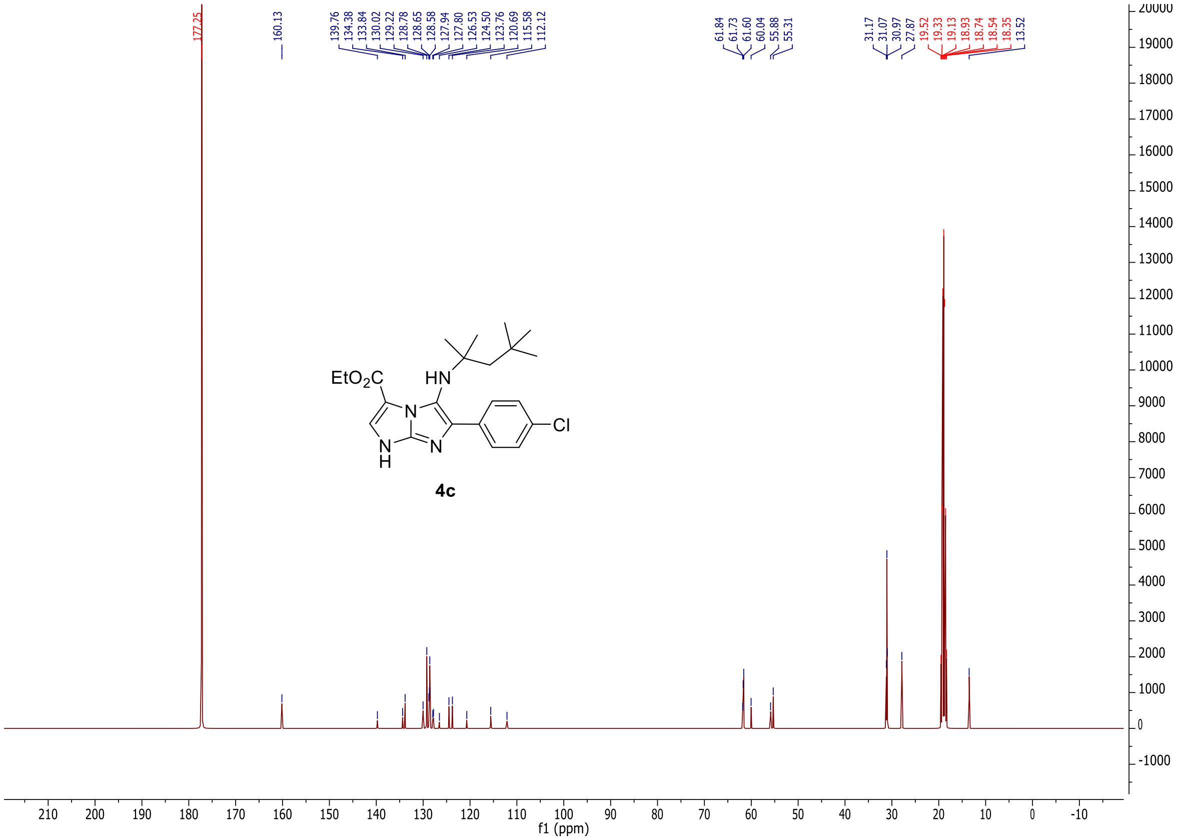
177.25
160.13
139.76 134.38 133.84 130.02 129.22 128.78 128.65 128.58 127.94 127.80 126.53 124.50 123.76 120.69 115.58 112.12
61.84 61.73 61.60 60.04 55.88 55.31
31.17 31.07 30.97 27.87 19.52 19.33 19.13 18.93 18.74 18.54 18.35 13.52
EtO2C
HN
N
N
H
Cl
4c
f1 (ppm)

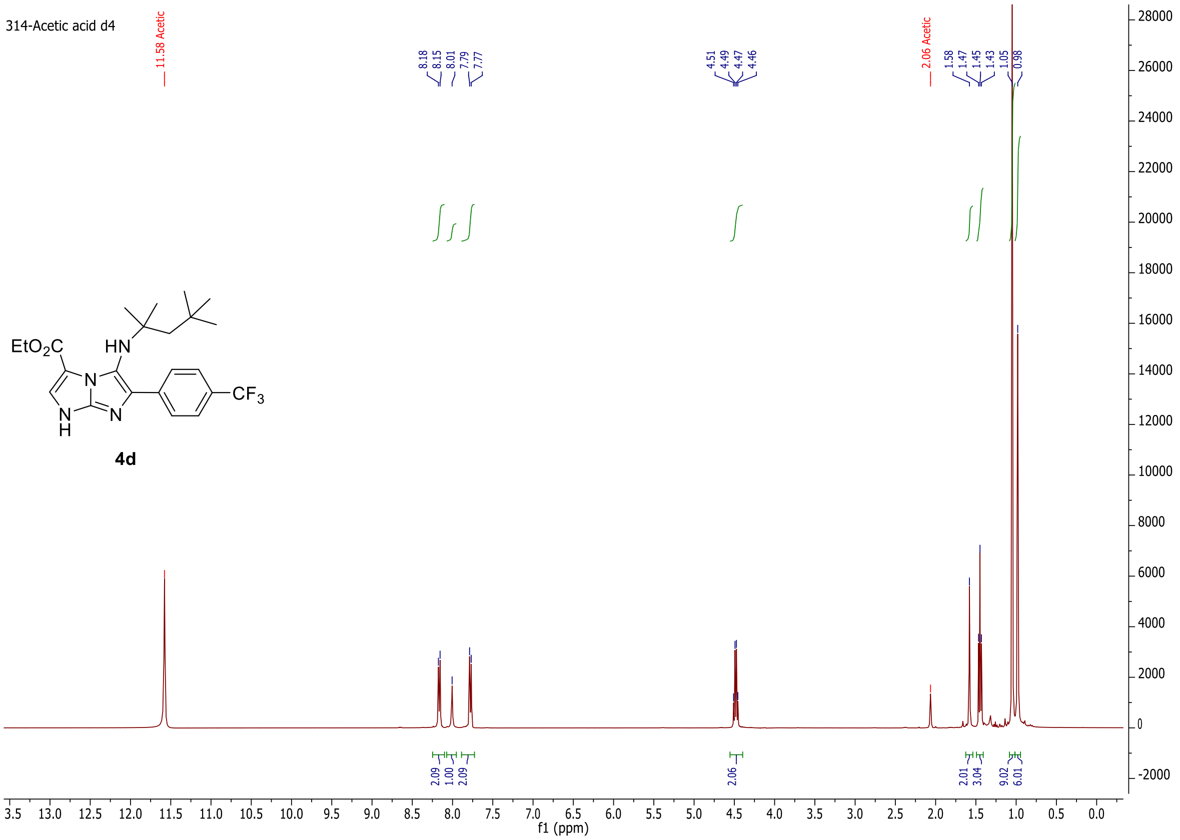
314-Acetic acid d4
11.58 Acetic
8.18
8.15
8.01
7.79
7.77
4.51
4.49
4.47
4.46
2.06 Acetic
1.58
1.47
1.45
1.43
1.05
0.98
2.09
1.00
2.09
2.06
2.01
3.04
9.02
6.01
EtO2C
HN
N
N
H
CF3
4d
f1 (ppm)

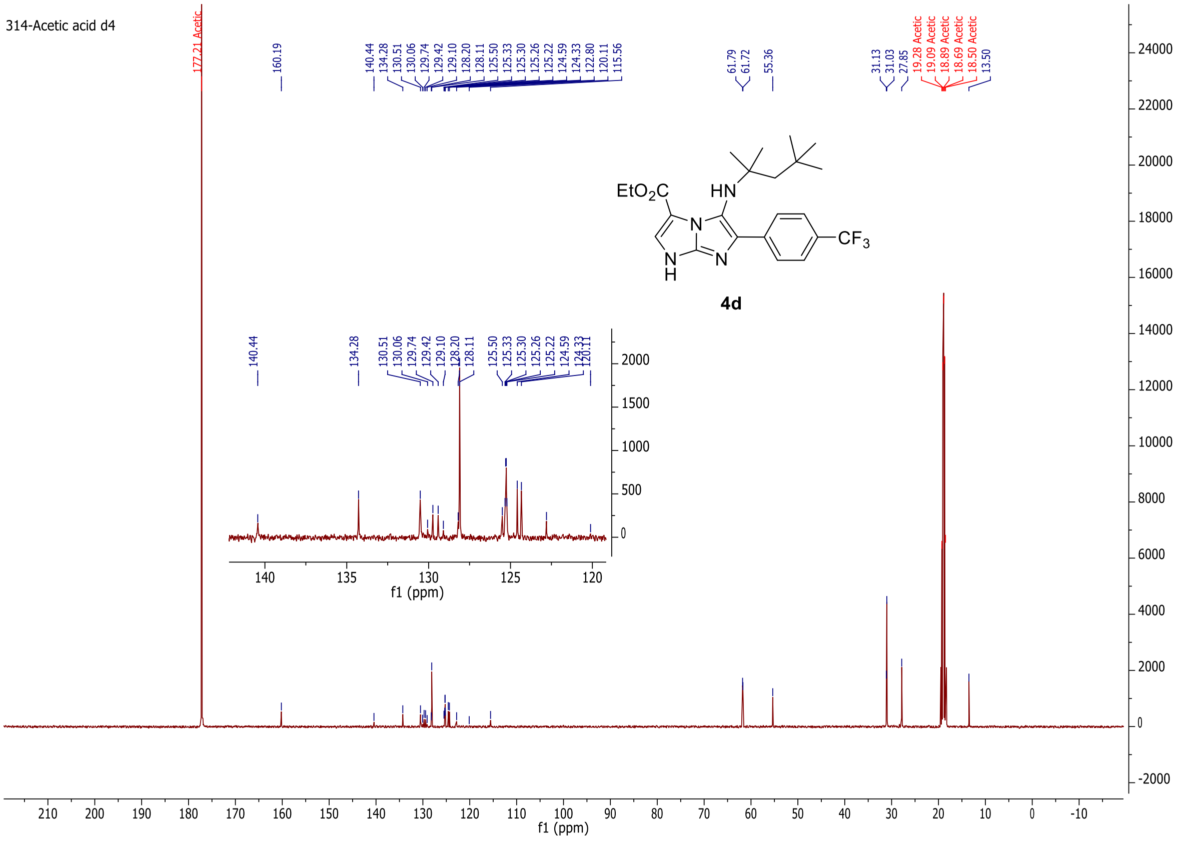
314-Acetic acid d4
177.21 Acetic
160.19
140.44
134.28
130.51
130.06
129.74
129.42
129.10
128.20
128.11
125.50
125.33
125.30
125.26
125.22
124.59
124.33
122.80
120.11
115.56
61.79
61.72
55.36
31.13
31.03
27.85
19.28 Acetic
19.09 Acetic
18.89 Acetic
18.69 Acetic
18.50 Acetic
13.50
EtO2C
HN
N
N
H
CF3
4d
f1 (ppm)

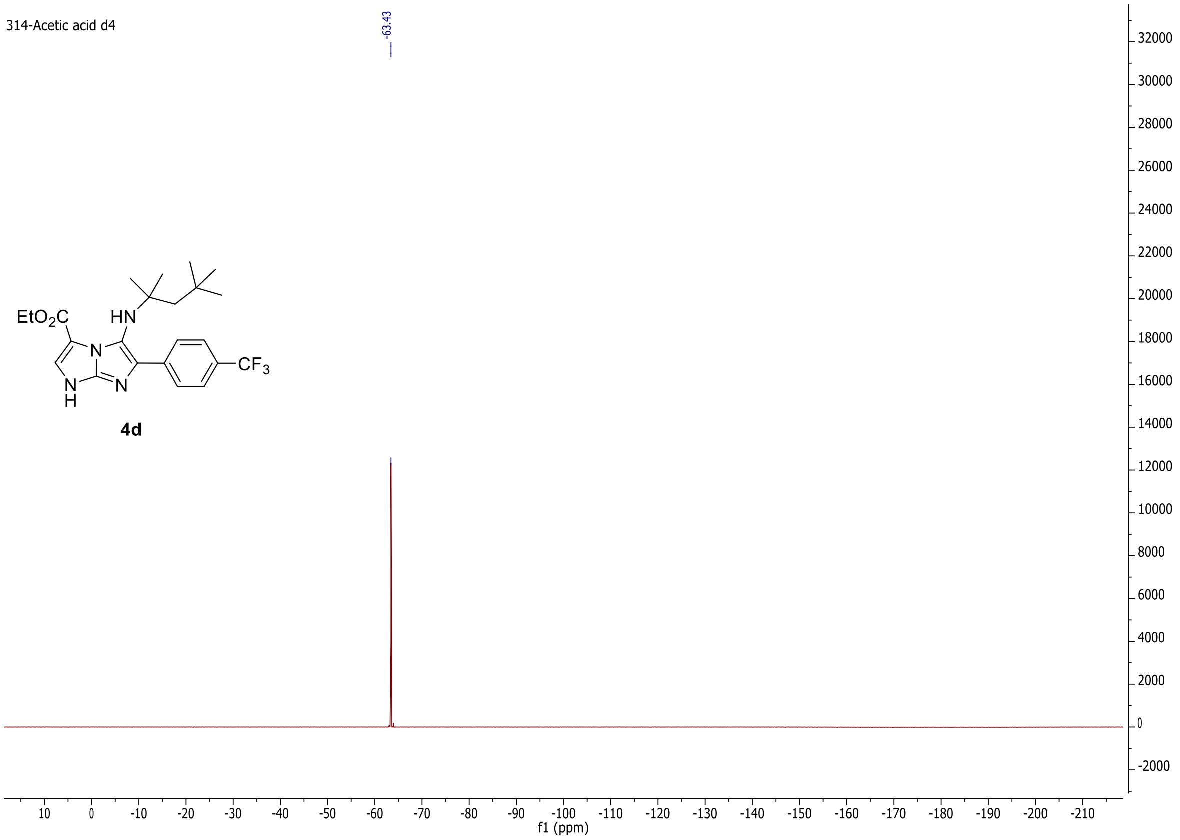
314-Acetic acid d4
-63.43
EtO2C
HN
N
N
N
H
CF3
4d
f1 (ppm)

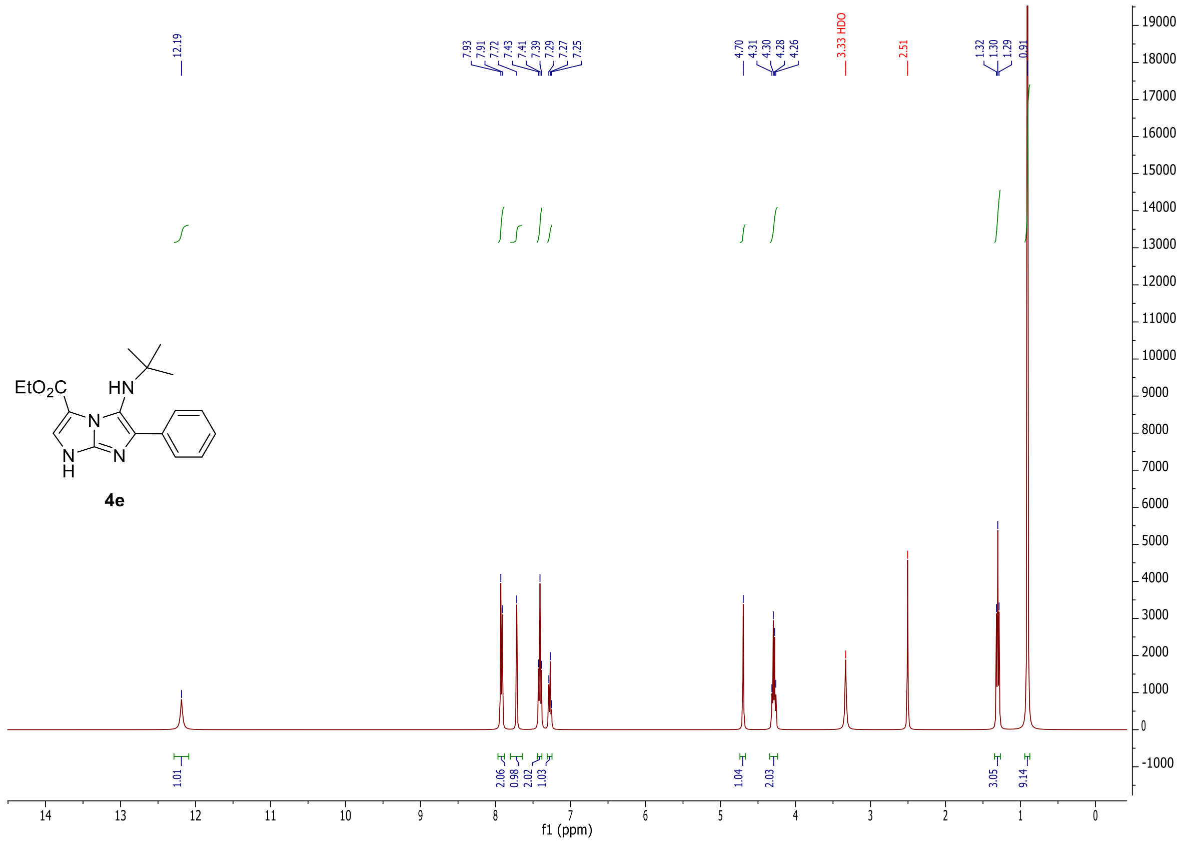

12.19
7.93
7.91
7.72
7.43
7.41
7.39
7.29
7.27
7.25
4.70
4.31
4.30
4.28
4.26
3.33 HDO
2.51
1.32
1.30
1.29
0.91
EtO$_2$C
HN
N
N
H
4e
1.01
2.06
0.98
2.02
1.03
1.04
2.03
3.05
9.14
f1 (ppm)

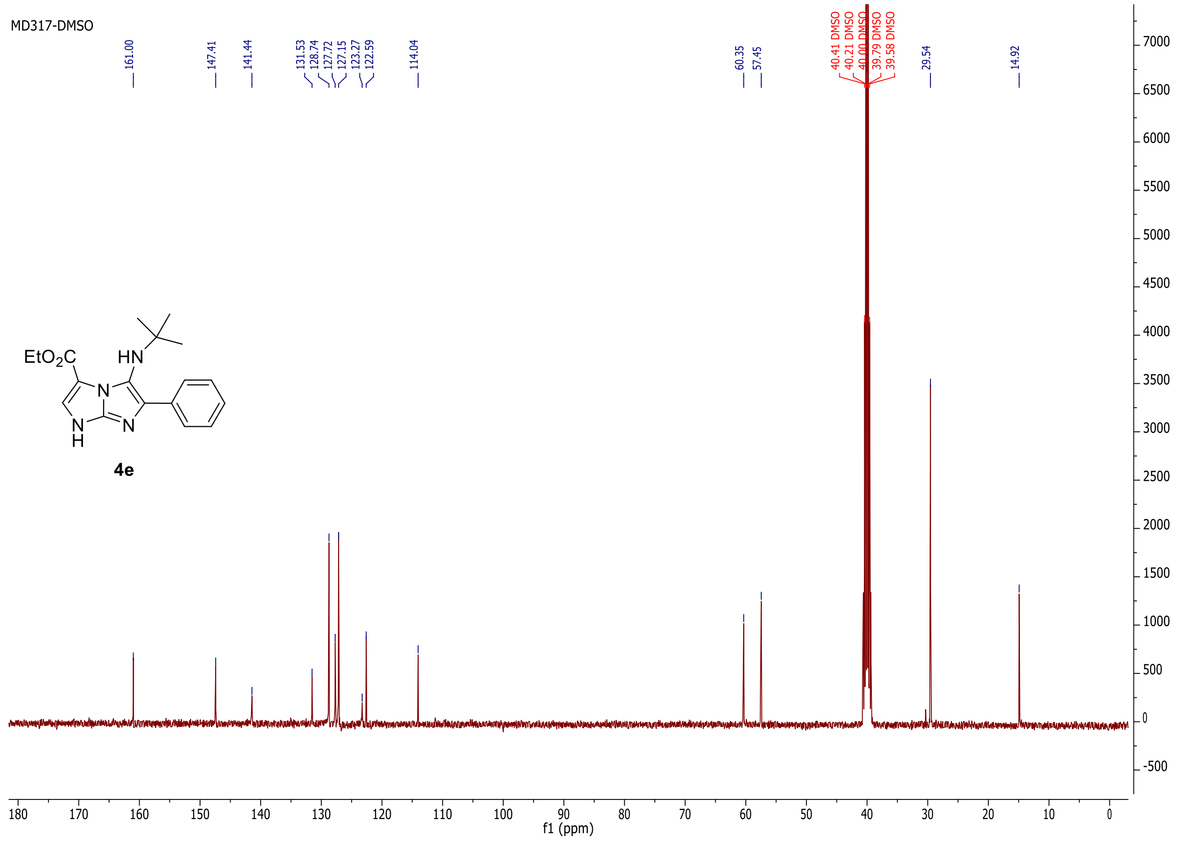

MD317-DMSO
161.00
147.41
141.44
131.53
128.74
127.72
127.15
123.27
122.59
114.04
60.35
57.45
40.41 DMSO
40.21 DMSO
40.00 DMSO
39.79 DMSO
39.58 DMSO
29.54
14.92
EtO$_2$C
HN
N
N
H
4e
f1 (ppm)

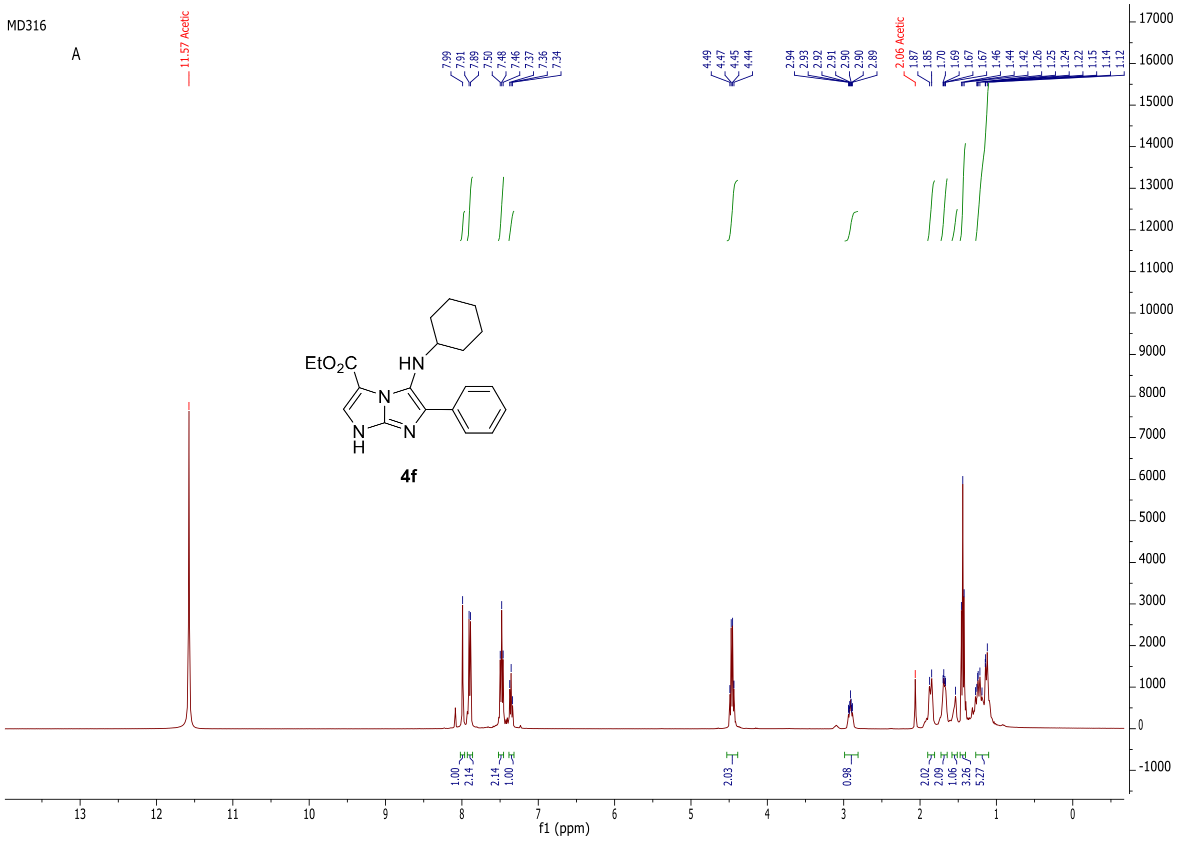

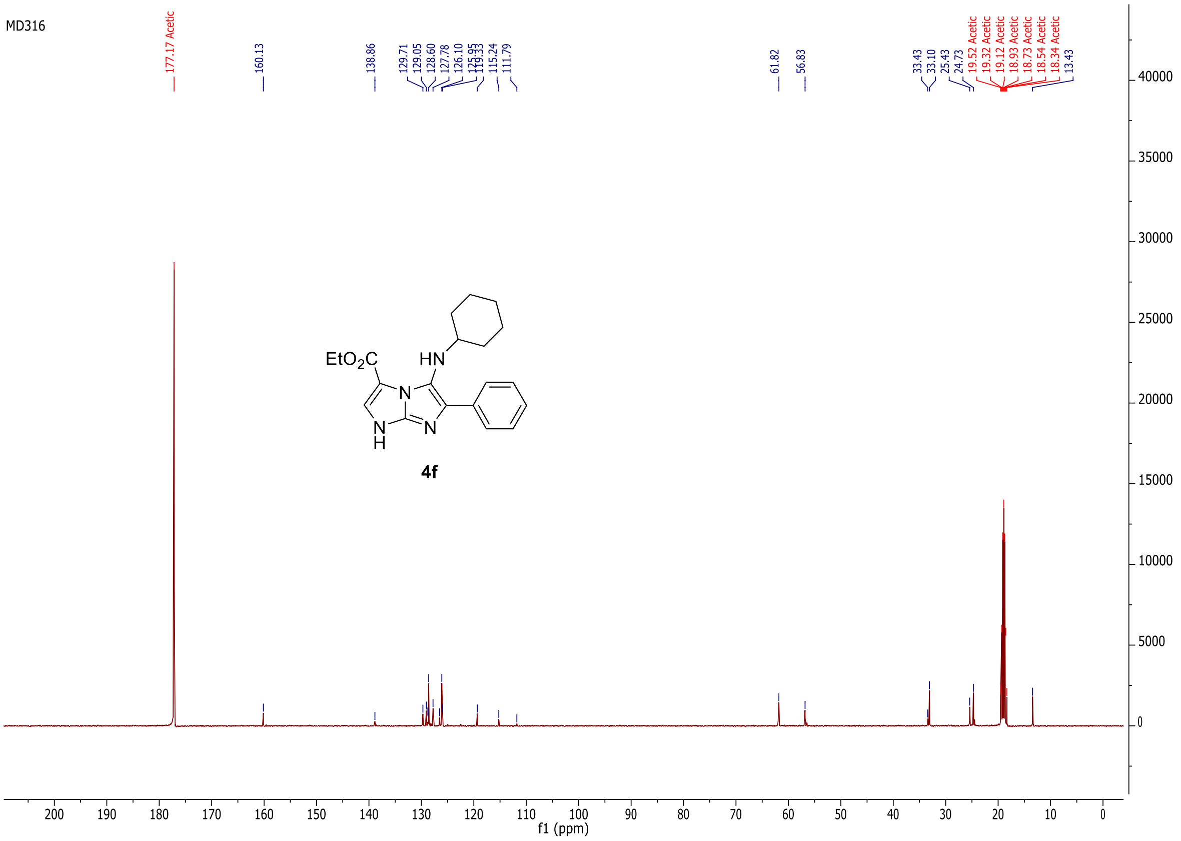

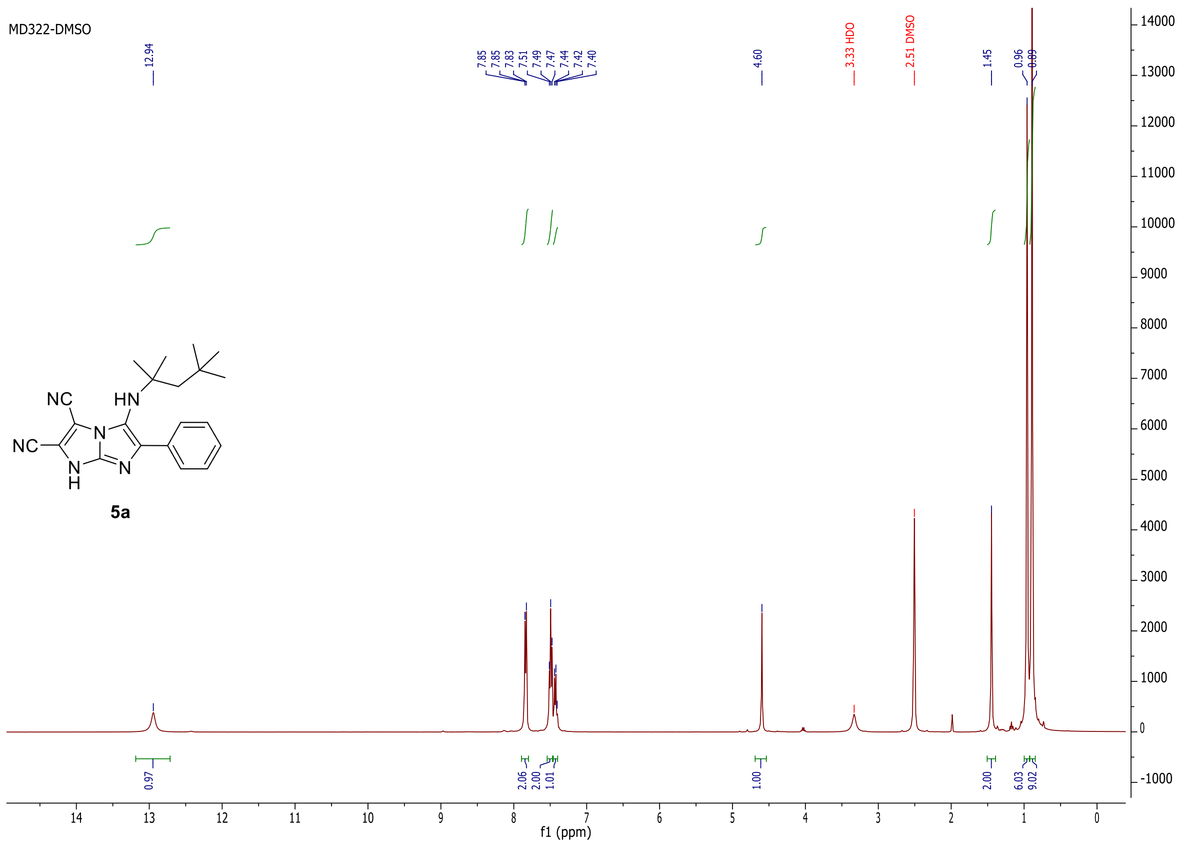

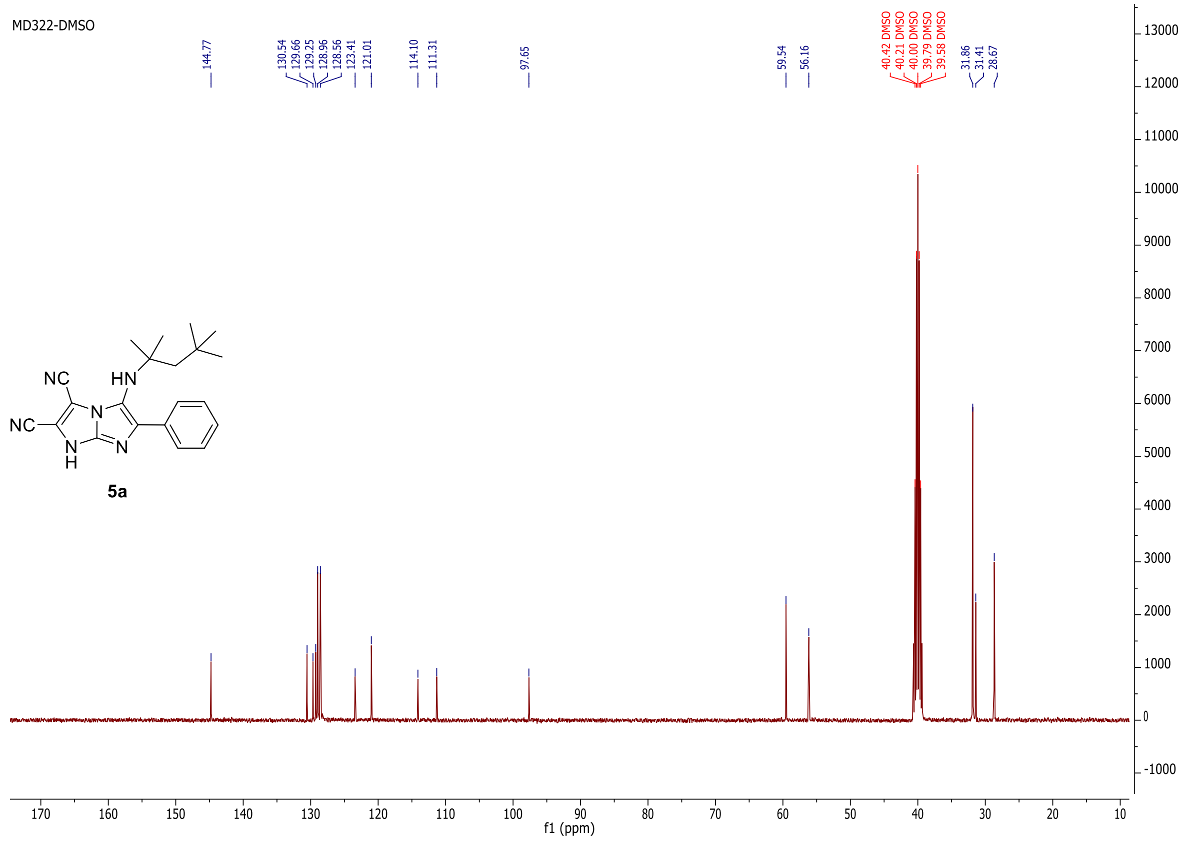

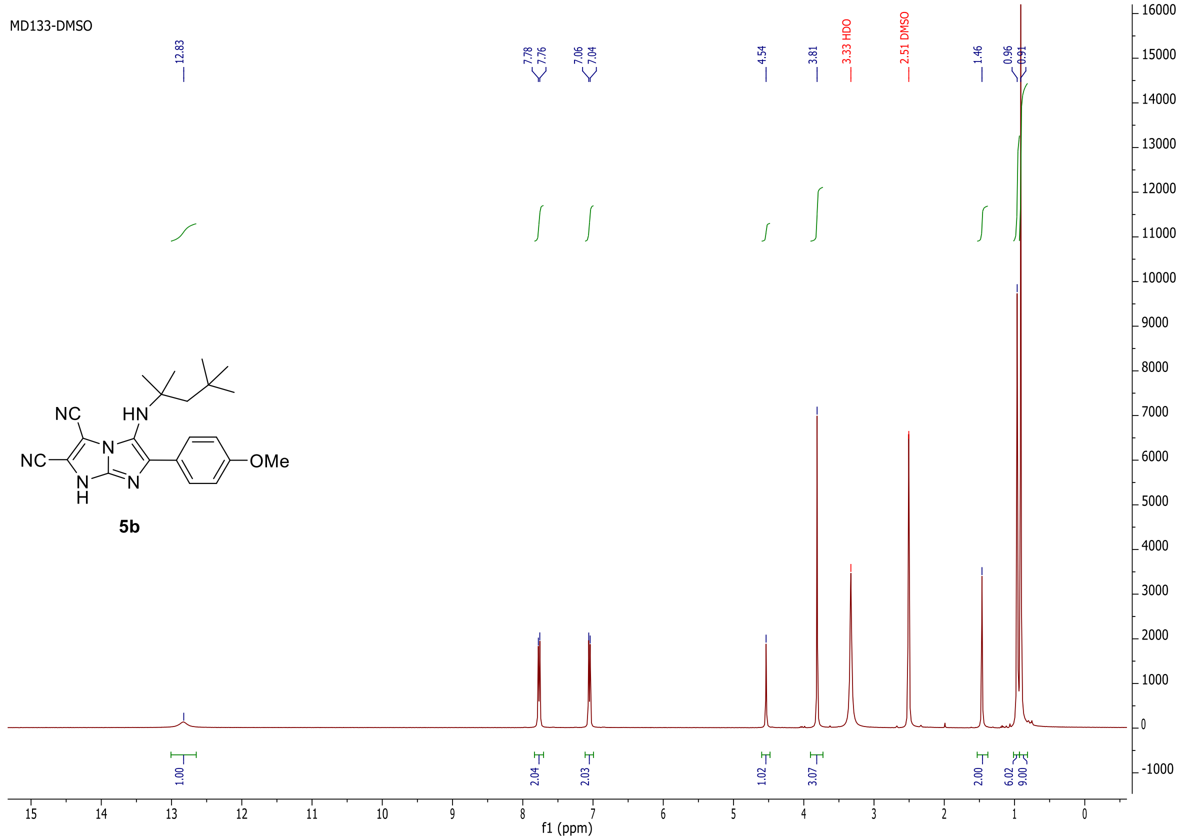
MD133-DMSO
12.83
7.78
7.76
7.06
7.04
4.54
3.81
3.33 HDO
2.51 DMSO
1.46
0.96
0.91
1.00
2.04
2.03
1.02
3.07
2.00
6.02
9.00
f1 (ppm)
NC
HN
NC
OMe
N
H
N
N
5b

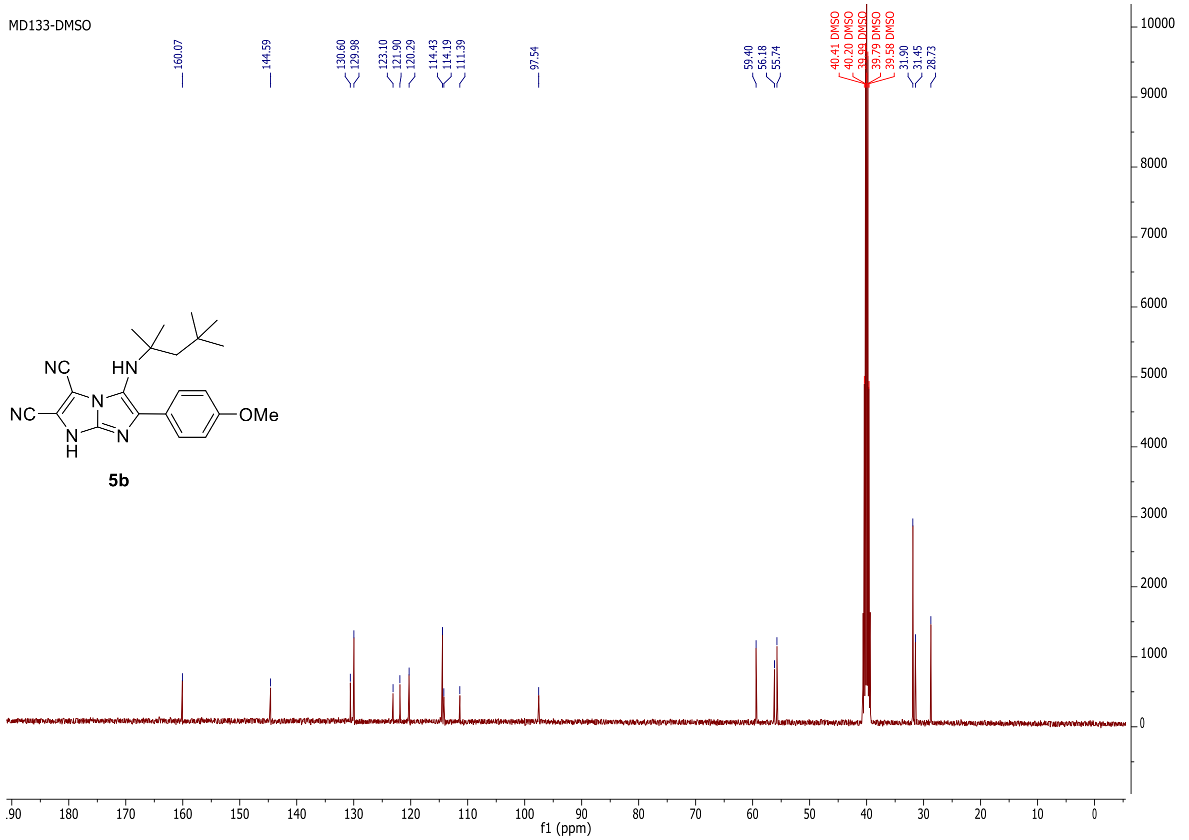
MD133-DMSO
160.07
144.59
130.60
129.98
123.10
121.90
120.29
114.43
114.19
111.39
97.54
59.40
56.18
55.74
40.41 DMSO
40.20 DMSO
39.99 DMSO
39.79 DMSO
39.58 DMSO
31.90
31.45
28.73
f1 (ppm)
NC
HN
NC
OMe
N
H
N
N
5b

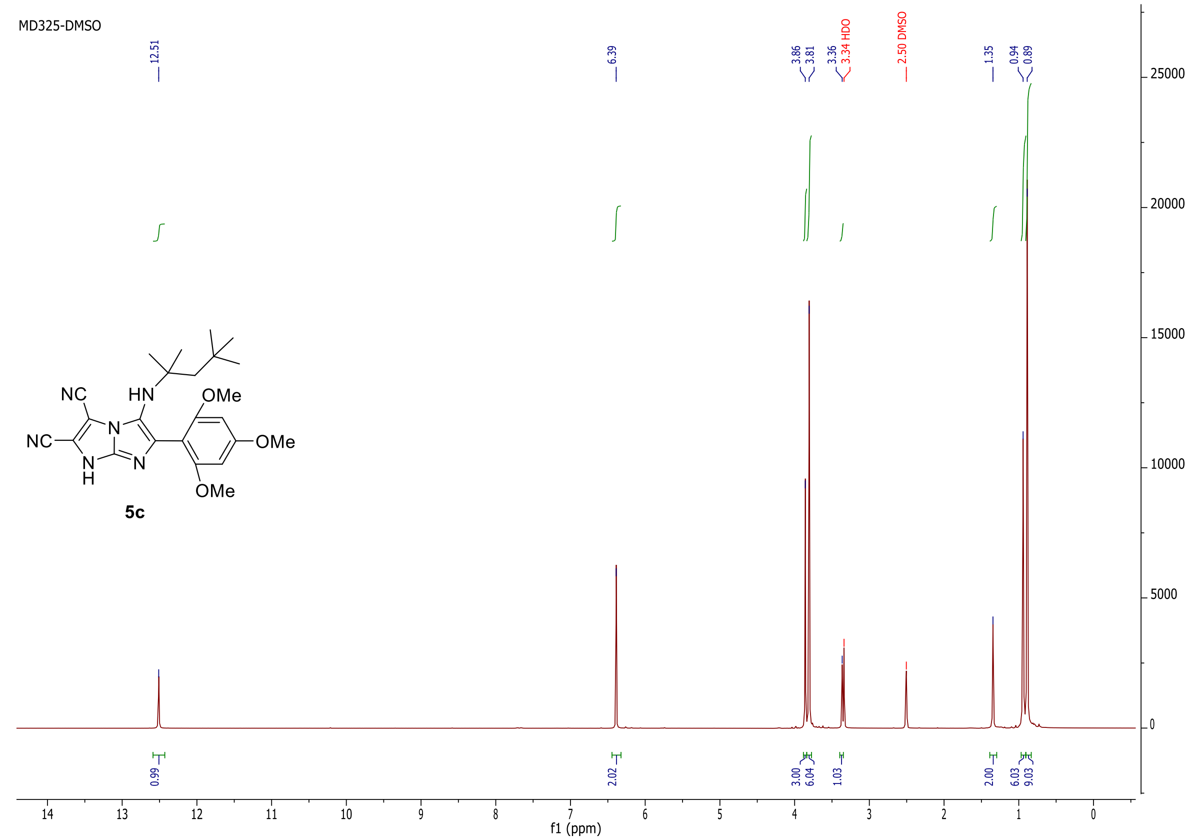
MD325-DMSO
12.51
6.39
3.86
3.81
3.36
3.34 HDO
2.50 DMSO
1.35
0.94
0.89
NC
HN
OMe
NC
N
OMe
N
H
N
OMe
5c
0.99
2.02
3.00
6.04
1.03
2.00
6.03
9.03
f1 (ppm)

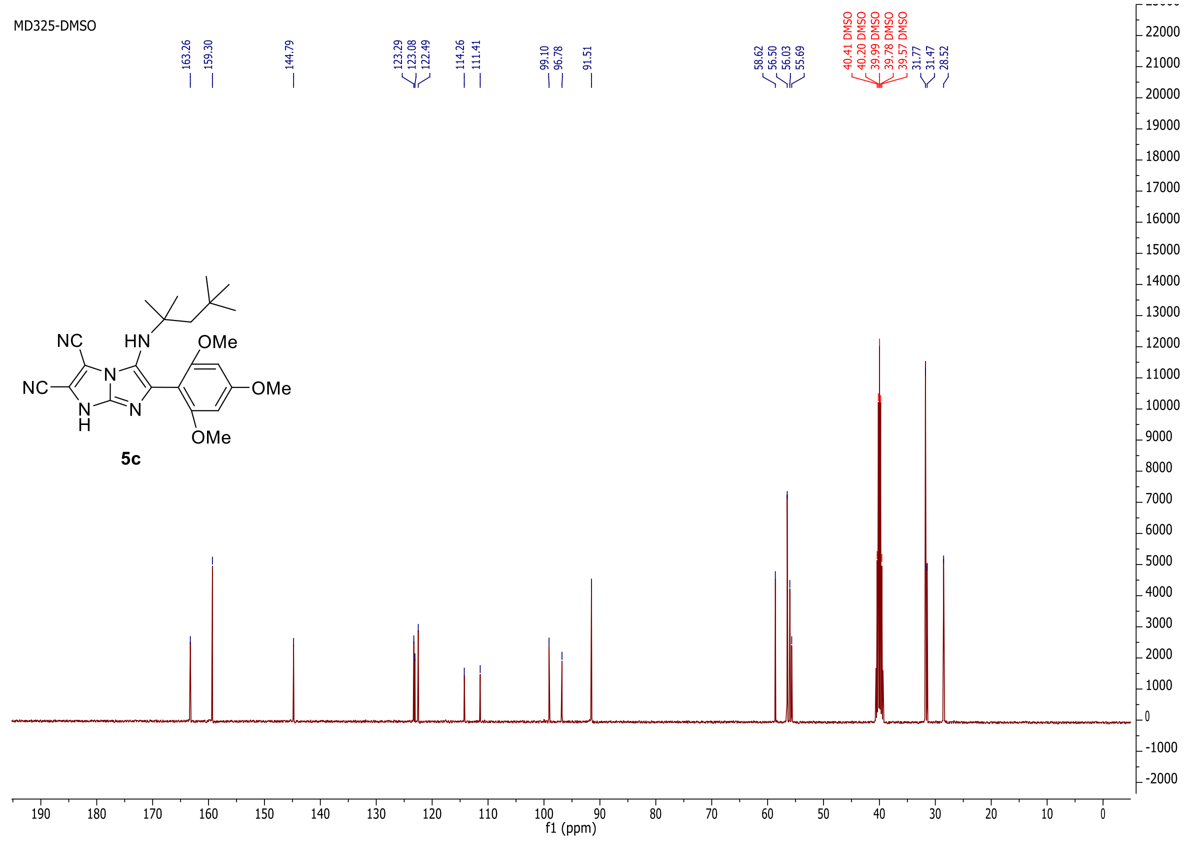
MD325-DMSO
163.26
159.30
144.79
123.29
123.08
122.49
114.26
111.41
99.10
96.78
91.51
58.62
56.50
56.03
55.69
40.41 DMSO
40.20 DMSO
39.99 DMSO
39.78 DMSO
39.57 DMSO
31.77
31.47
28.52
NC
HN
OMe
NC
N
OMe
N
H
N
OMe
5c
f1 (ppm)

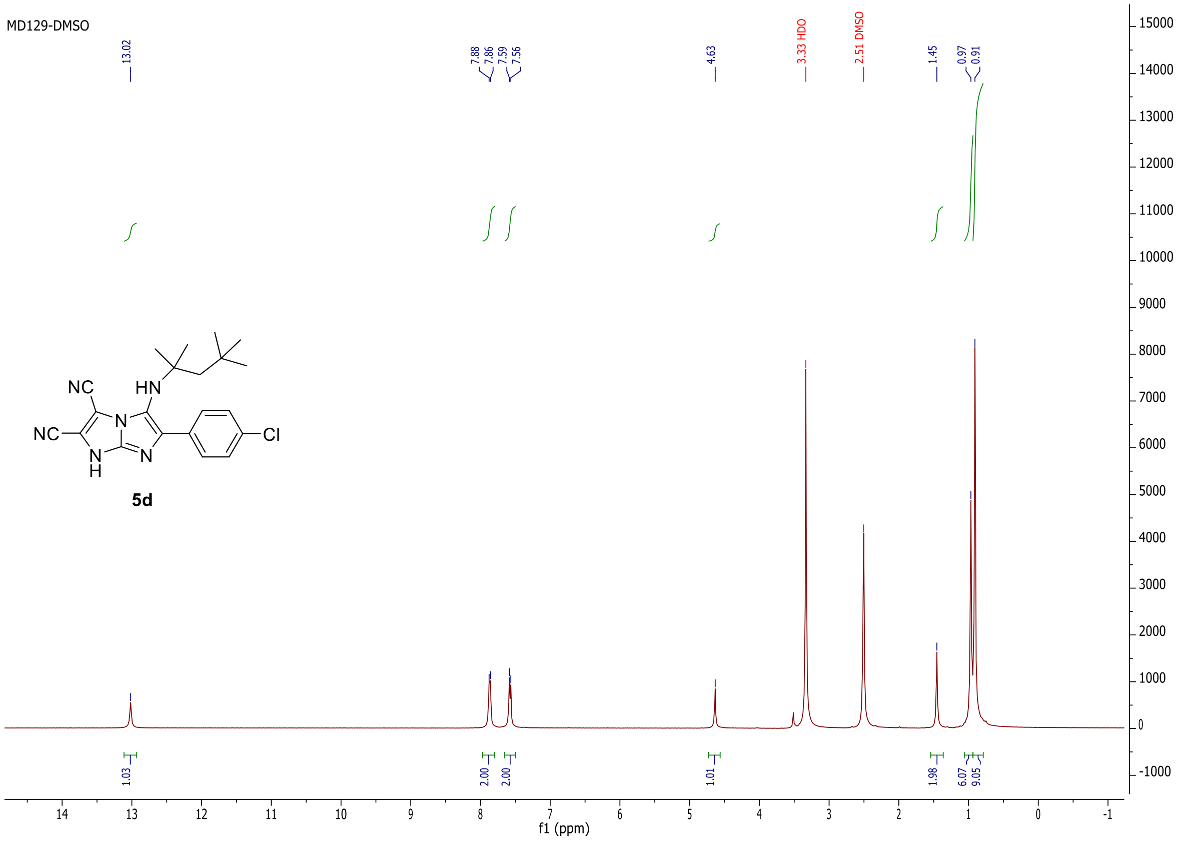
MD129-DMSO
13.02
7.88
7.86
7.59
7.56
4.63
3.33 HDO
2.51 DMSO
1.45
0.97
0.91
1.03
2.00
2.00
1.01
1.98
6.07
9.05
f1 (ppm)
NC
HN
NC
N
N
H
N
Cl
5d

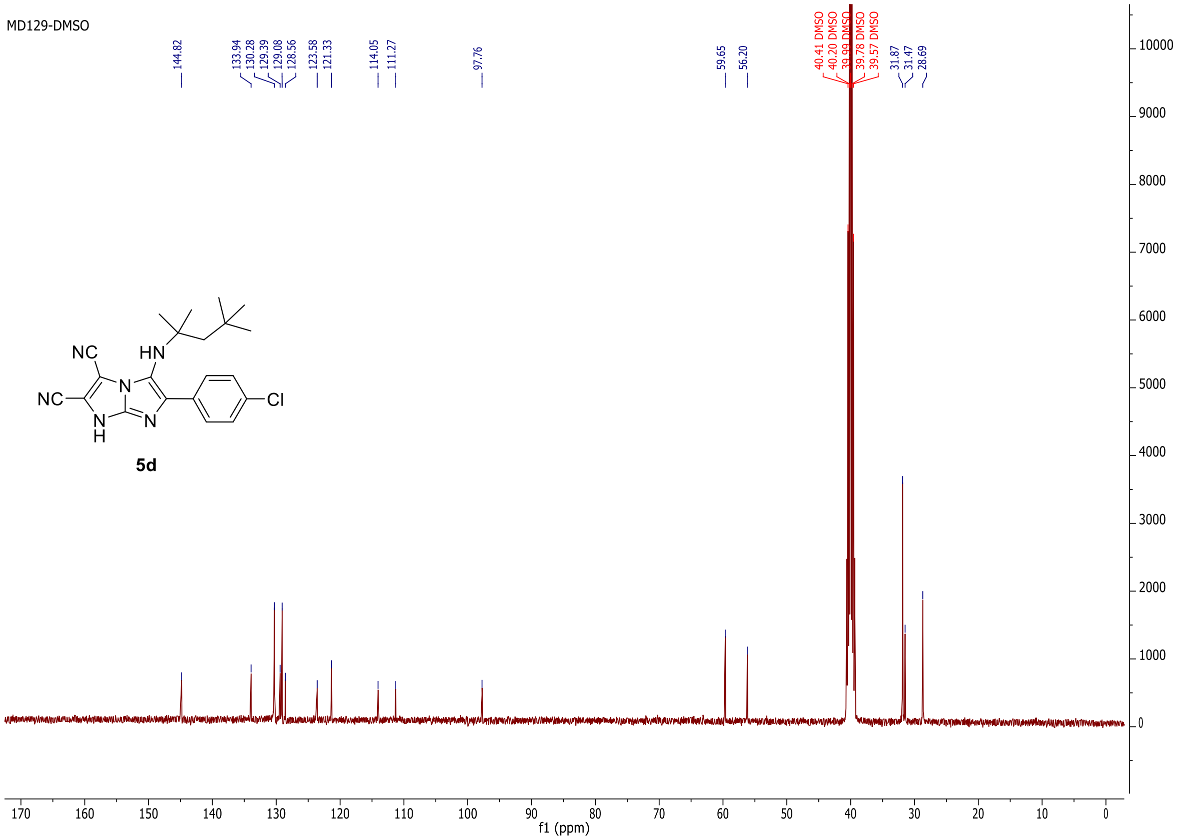
MD129-DMSO
144.82
133.94
130.28
129.39
129.08
128.56
123.58
121.33
114.05
111.27
97.76
59.65
56.20
40.41 DMSO
40.20 DMSO
39.99 DMSO
39.78 DMSO
39.57 DMSO
31.87
31.47
28.69
f1 (ppm)
NC
HN
NC
N
N
H
N
Cl
5d

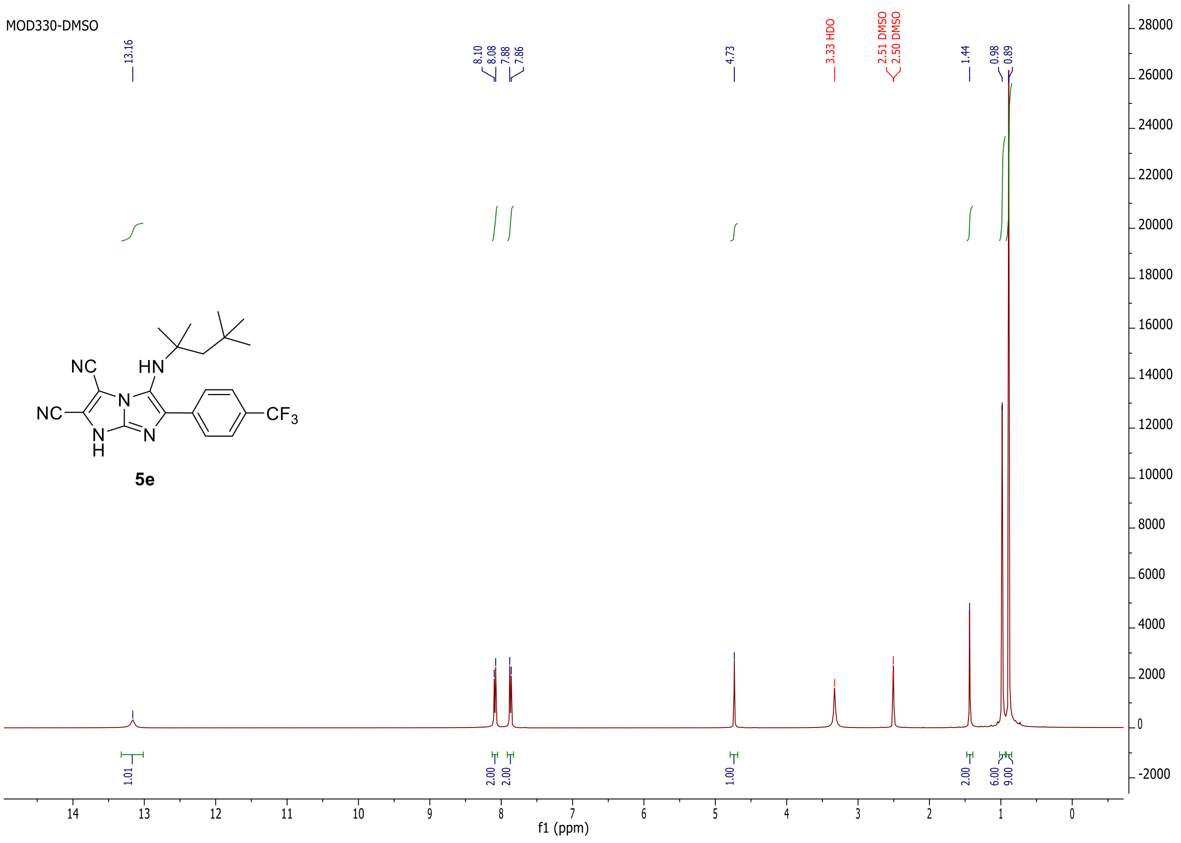
MOD330-DMSO
5e
f1 (ppm)

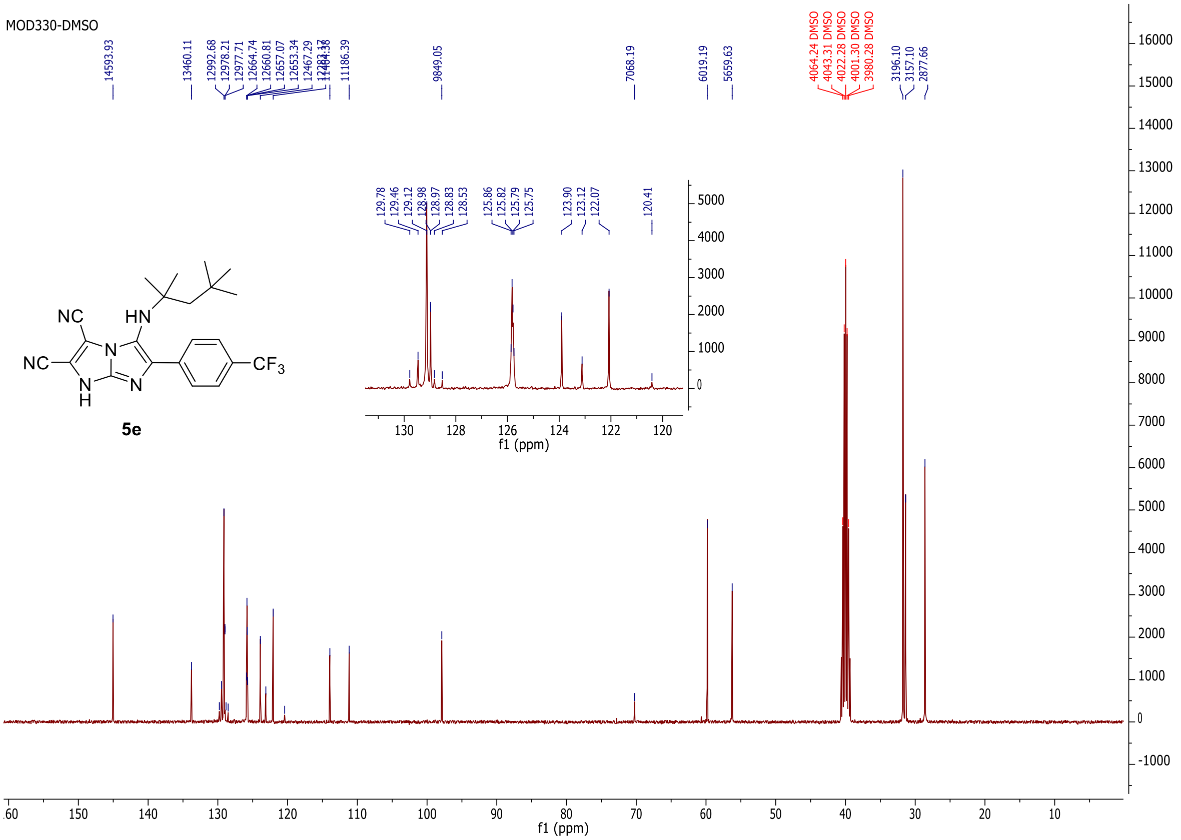
MOD330-DMSO
5e
f1 (ppm)

MOD330-DMSO

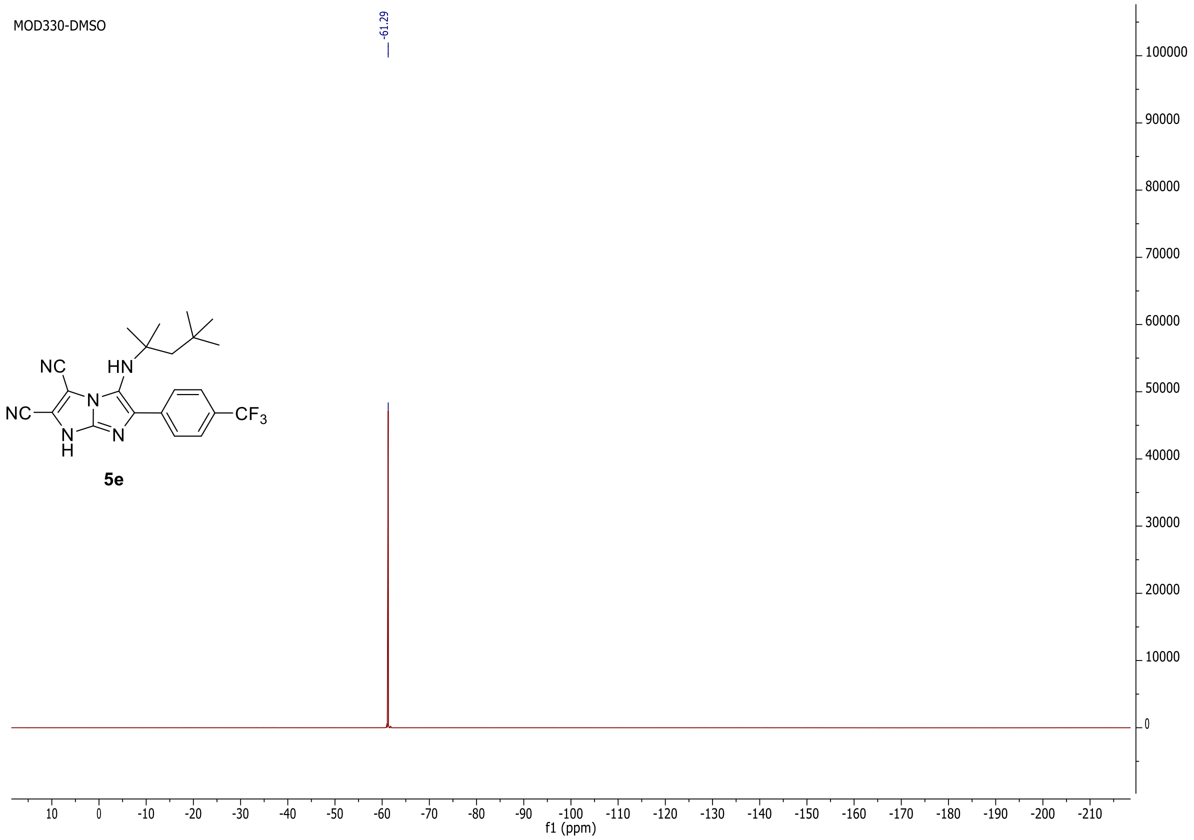

5e

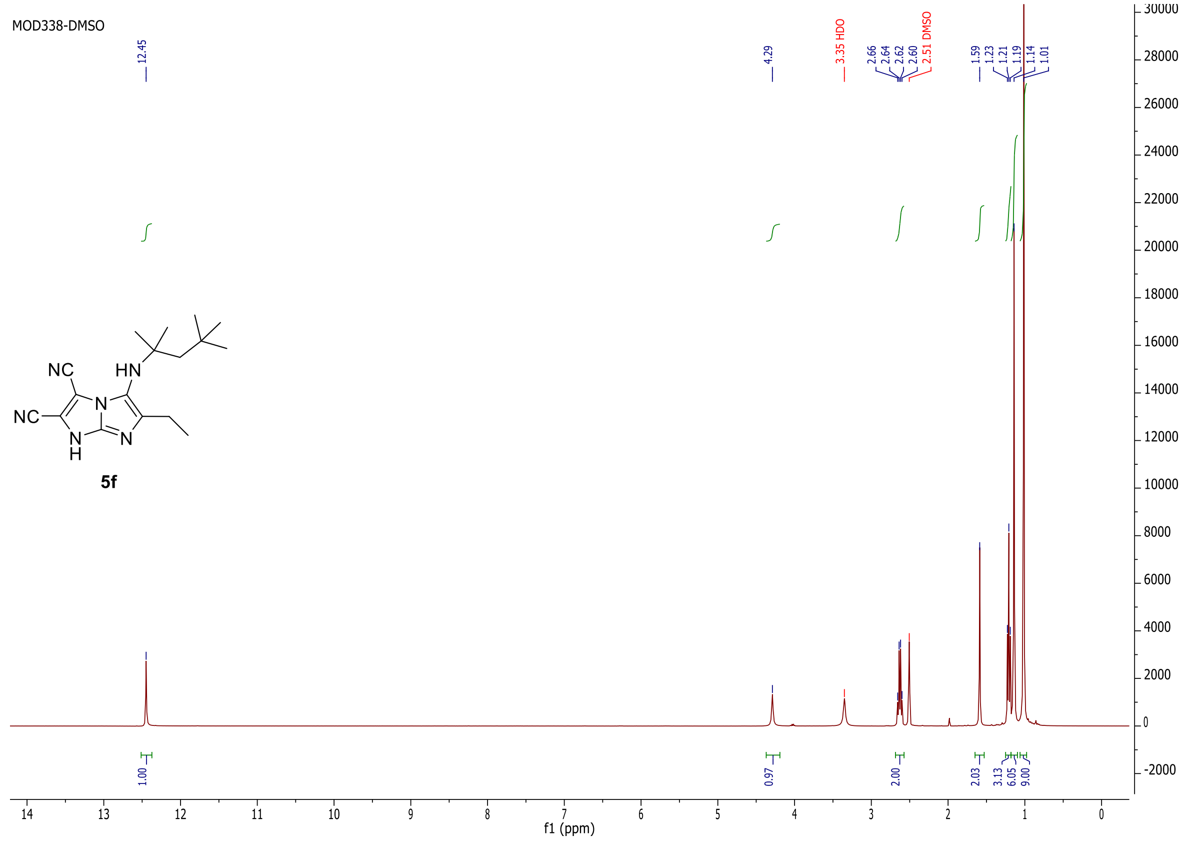
MOD338-DMSO
12.45
4.29
3.35 HDO
2.66
2.64
2.62
2.60
2.51 DMSO
1.59
1.23
1.21
1.19
1.14
1.01
NC
HN
NC
N
H
N
5f
1.00
0.97
2.00
2.03
3.13
6.05
9.00
f1 (ppm)

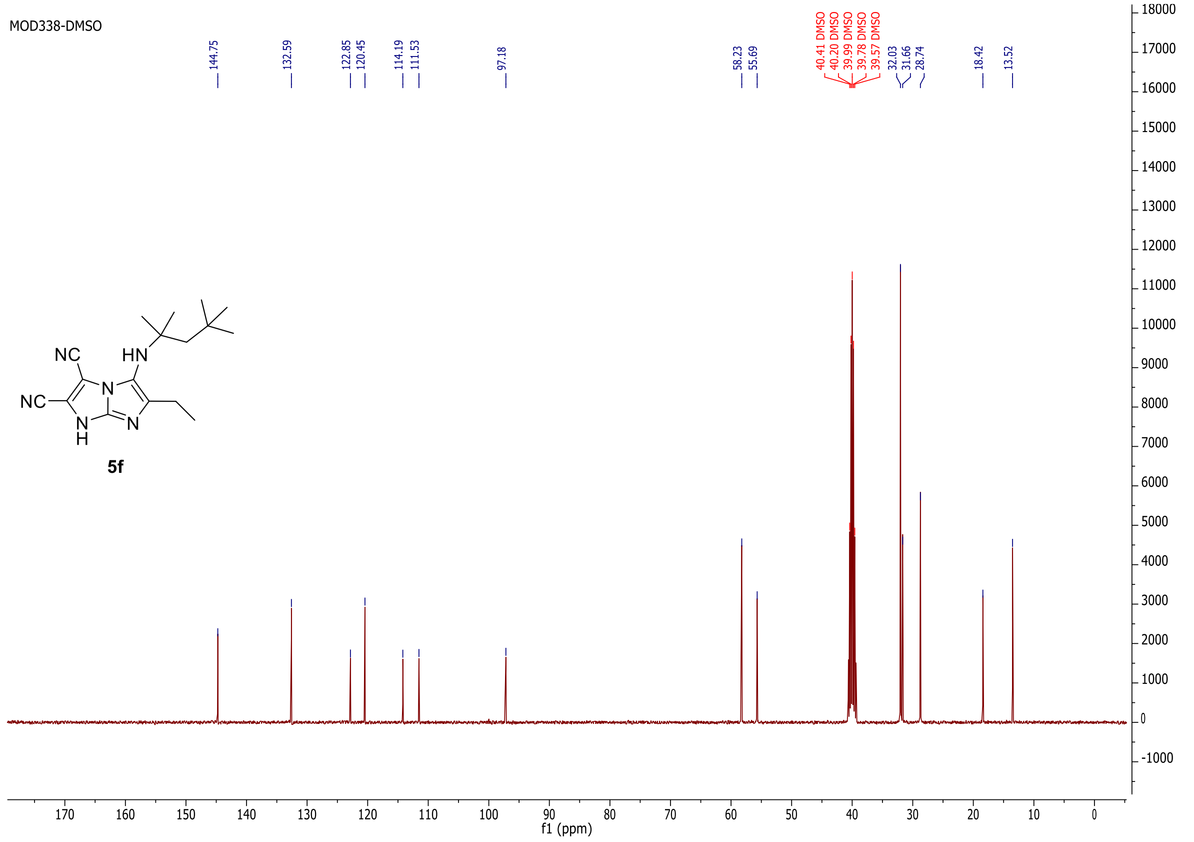
MOD338-DMSO
144.75
132.59
122.85
120.45
114.19
111.53
97.18
58.23
55.69
40.41 DMSO
40.20 DMSO
39.99 DMSO
39.78 DMSO
39.57 DMSO
32.03
31.66
28.74
18.42
13.52
NC
HN
NC
N
H
N
5f
f1 (ppm)

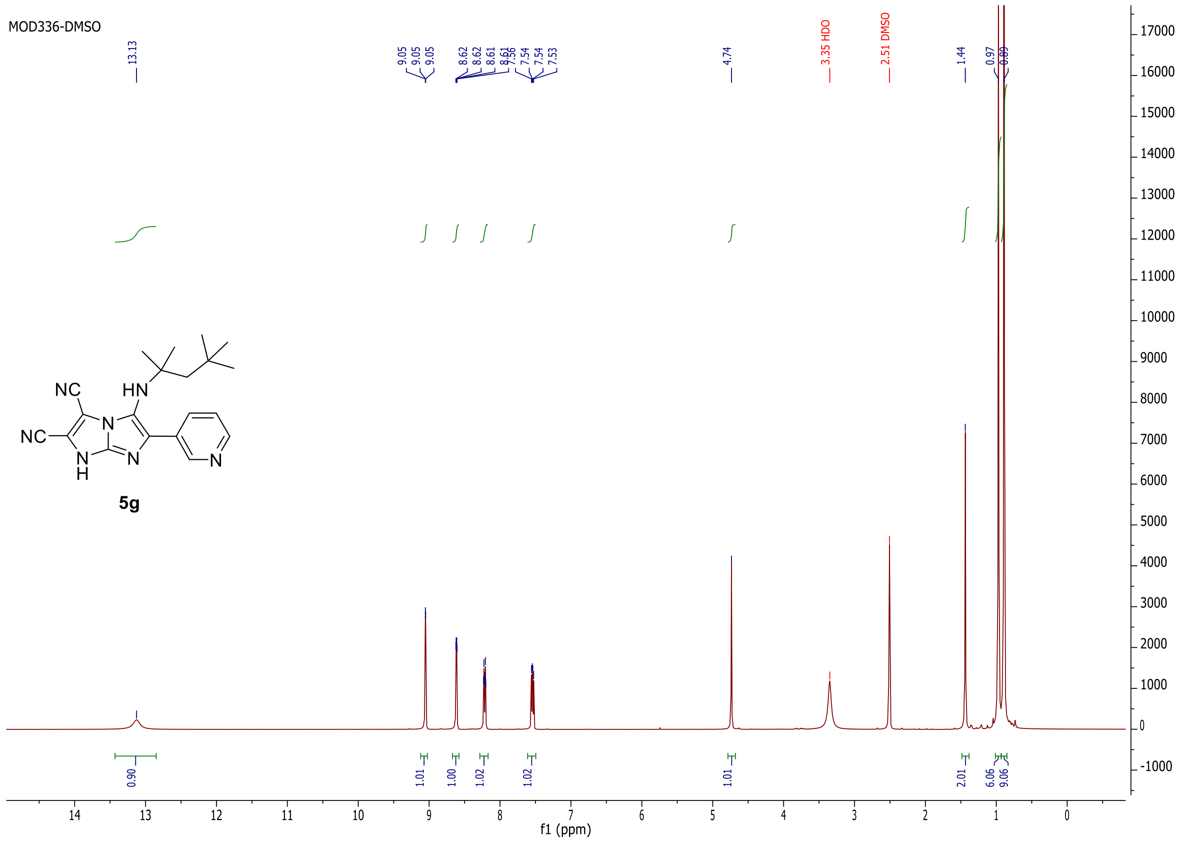
MOD336-DMSO
13.13
9.05
9.05
9.05
8.62
8.62
8.61
8.61
7.56
7.54
7.54
7.53
4.74
3.35 HDO
2.51 DMSO
1.44
0.97
0.89
0.90
1.01
1.00
1.02
1.02
1.01
2.01
6.06
9.06
NC
HN
NC
N
H
N
N
5g
f1 (ppm)

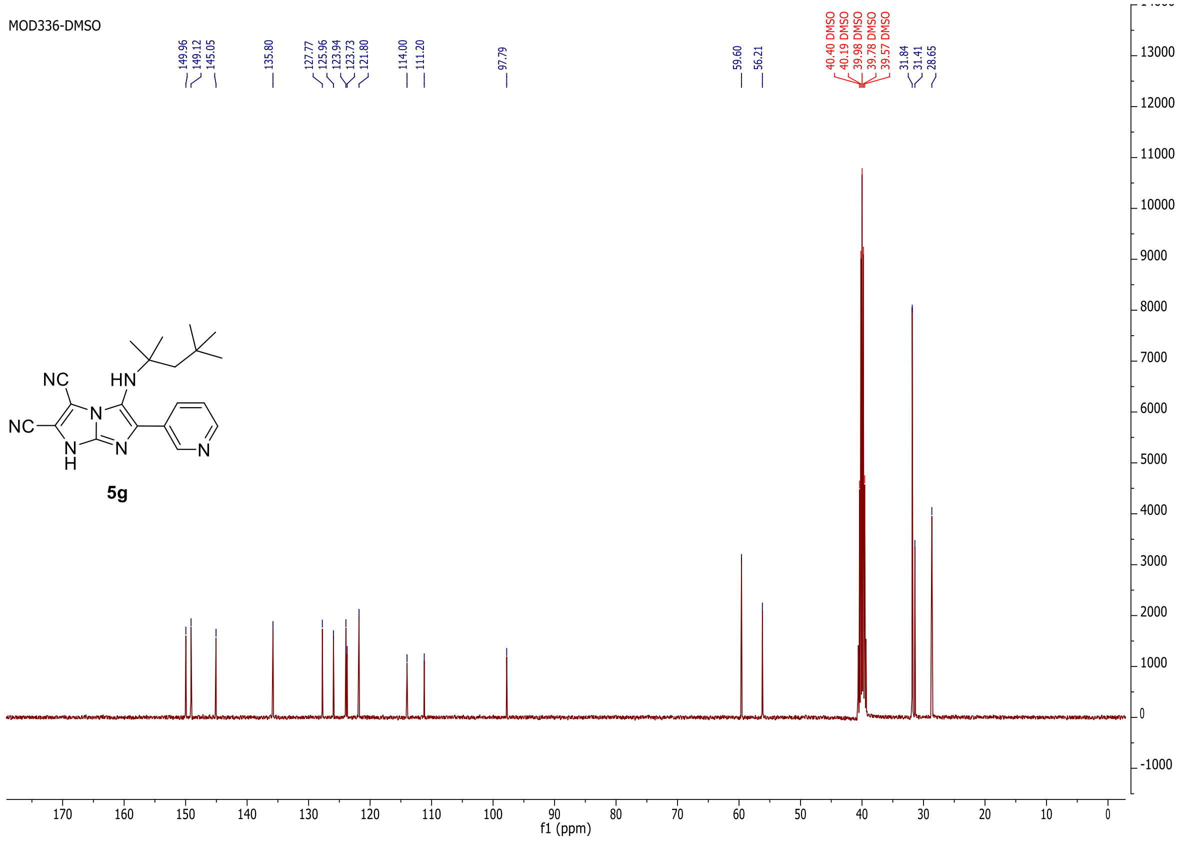
MOD336-DMSO
149.96
149.12
145.05
135.80
127.77
125.96
123.94
123.73
121.80
114.00
111.20
97.79
59.60
56.21
40.40 DMSO
40.19 DMSO
39.98 DMSO
39.78 DMSO
39.57 DMSO
31.84
31.41
28.65
NC
HN
NC
N
H
N
N
5g
f1 (ppm)

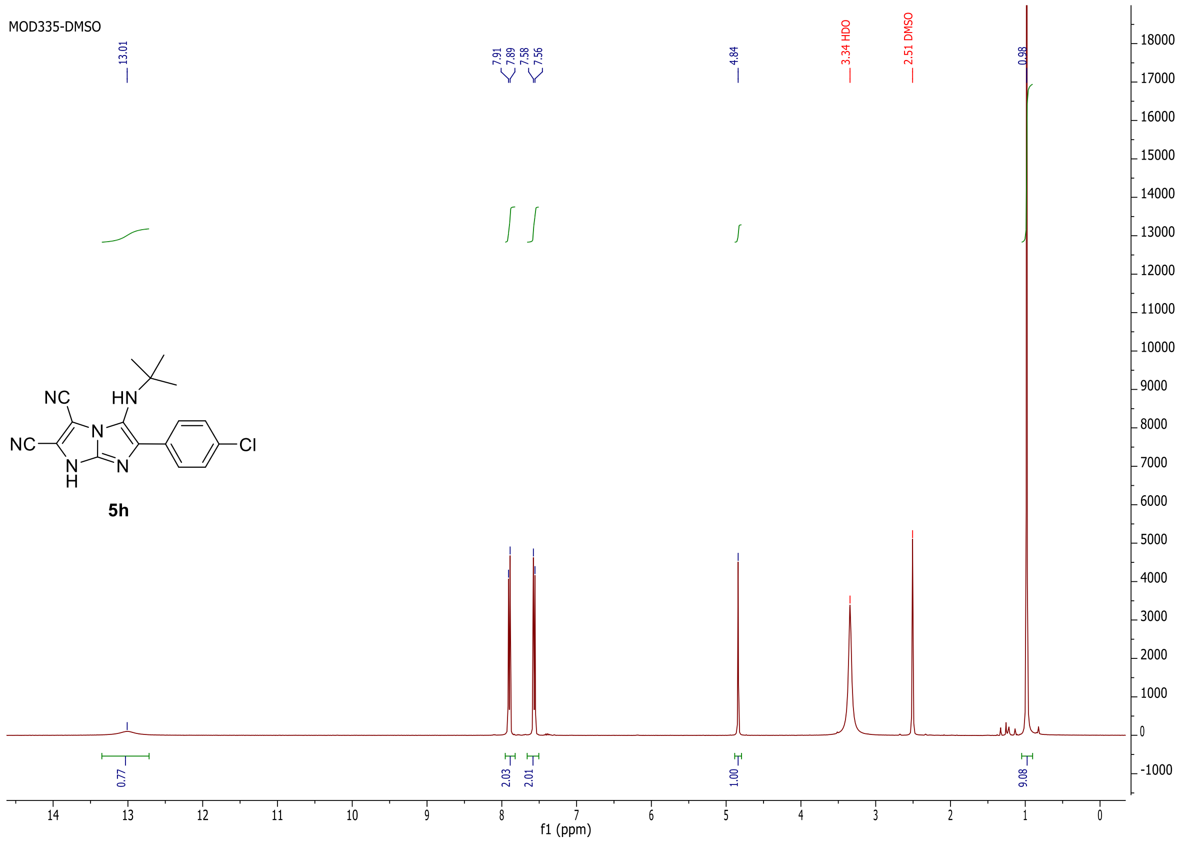
MOD335-DMSO
13.01
7.91
7.89
7.58
7.56
4.84
3.34 HDO
2.51 DMSO
0.98
0.77
2.03
2.01
1.00
9.08
NC
HN
NC
N
H
N
N
Cl
5h
f1 (ppm)

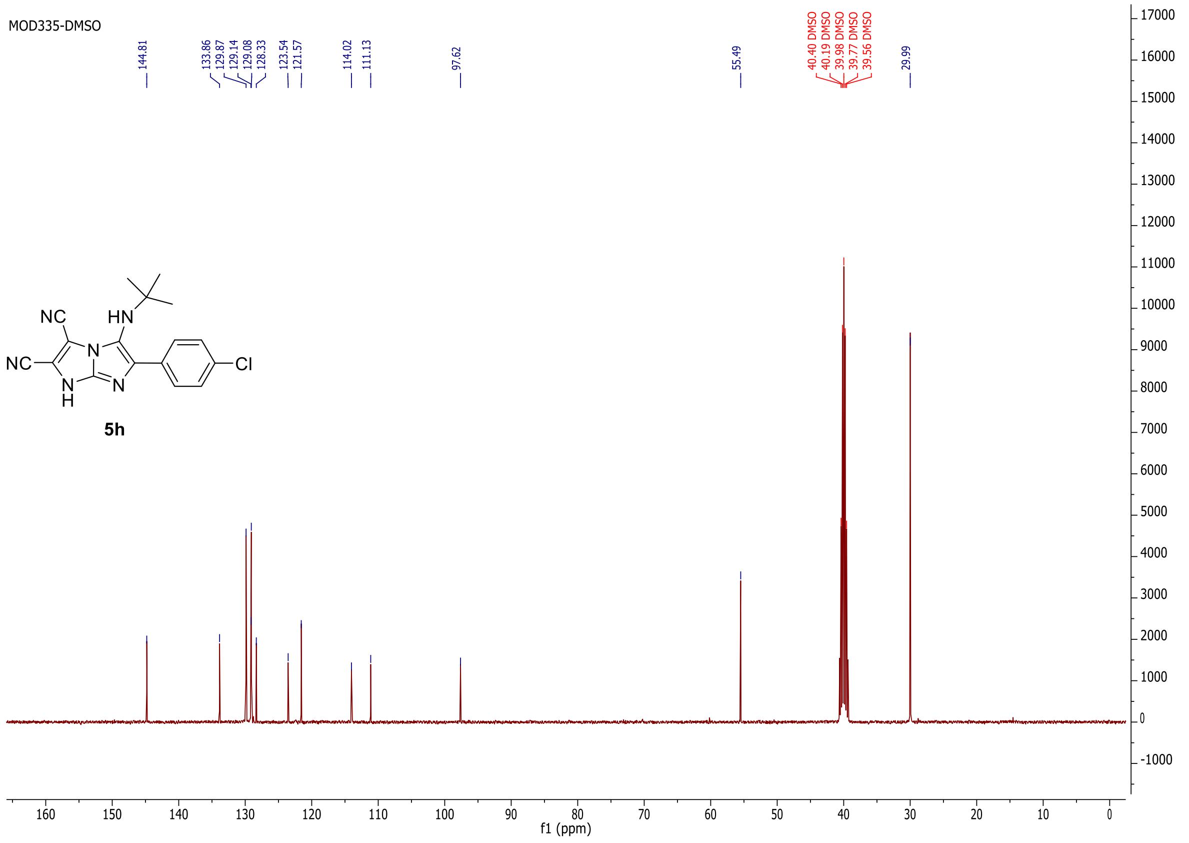
MOD335-DMSO
144.81
133.86
129.87
129.14
129.08
128.33
123.54
121.57
114.02
111.13
97.62
55.49
40.40 DMSO
40.19 DMSO
39.98 DMSO
39.77 DMSO
39.56 DMSO
29.99
NC
HN
NC
N
H
N
N
Cl
5h
f1 (ppm)

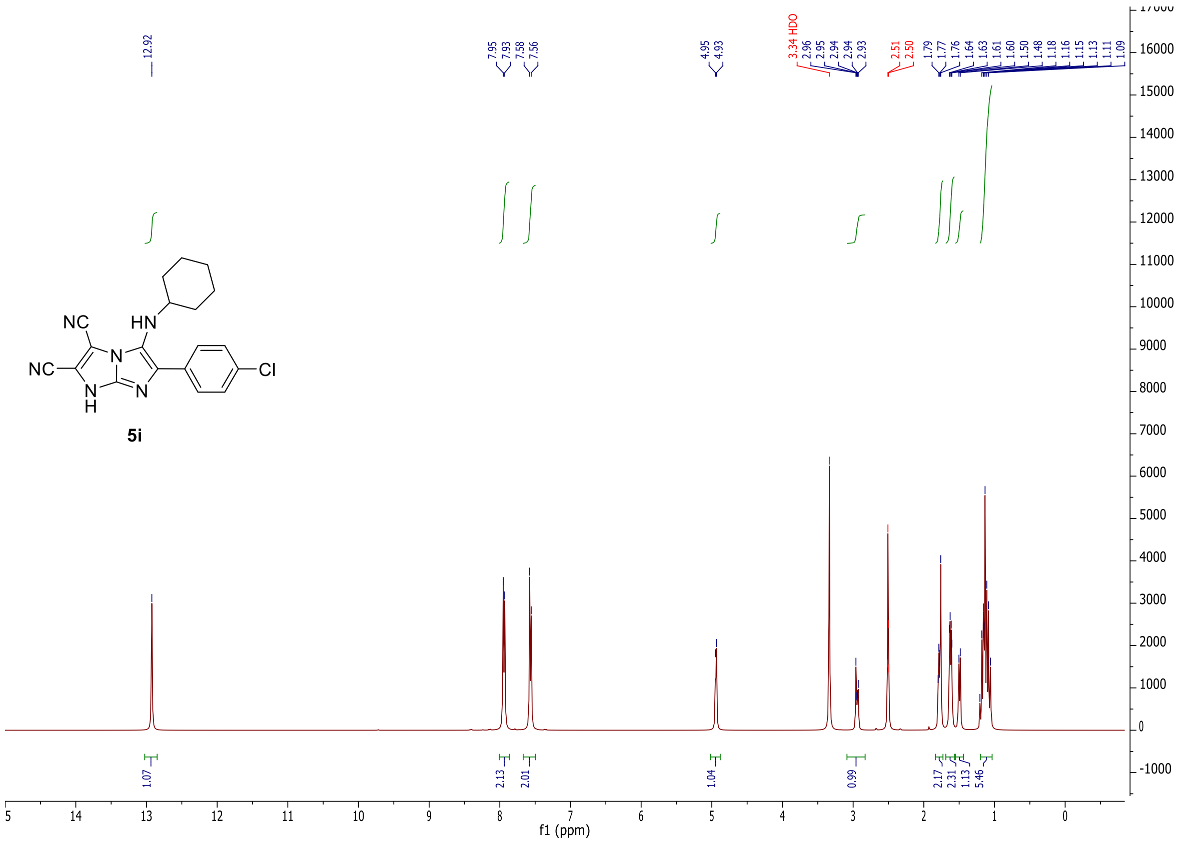
12.92
7.95
7.93
7.58
7.56
4.95
4.93
3.34 HDO
2.96
2.95
2.94
2.94
2.93
2.51
2.50
1.79
1.77
1.76
1.64
1.63
1.61
1.60
1.50
1.48
1.18
1.16
1.15
1.13
1.11
1.09
NC
HN
NC
N
N
N
H
Cl
5i
1.07
2.13
2.01
1.04
0.99
2.17
2.31
1.13
5.46
f1 (ppm)

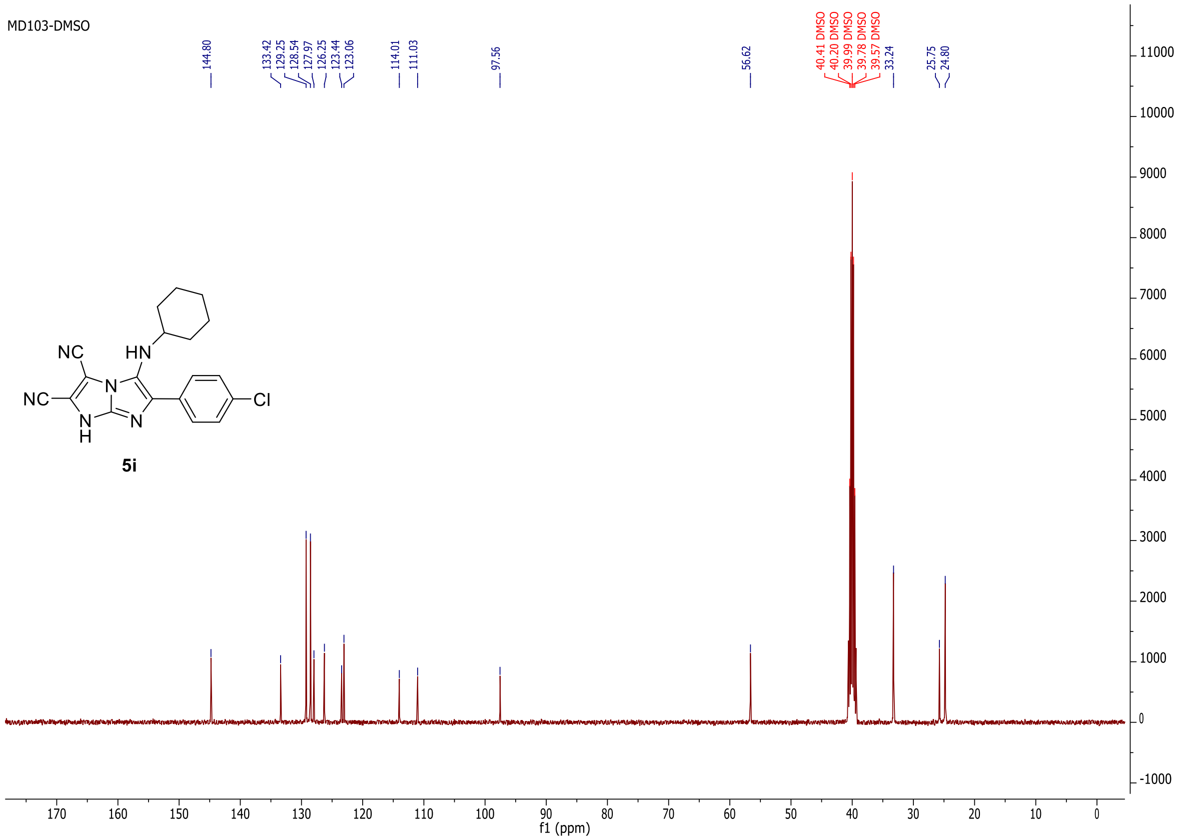
MD103-DMSO
144.80
133.42
129.25
128.54
127.97
126.25
123.44
123.06
114.01
111.03
97.56
56.62
40.41 DMSO
40.20 DMSO
39.99 DMSO
39.78 DMSO
39.57 DMSO
33.24
25.75
24.80
NC
HN
NC
N
N
N
H
Cl
5i
f1 (ppm)

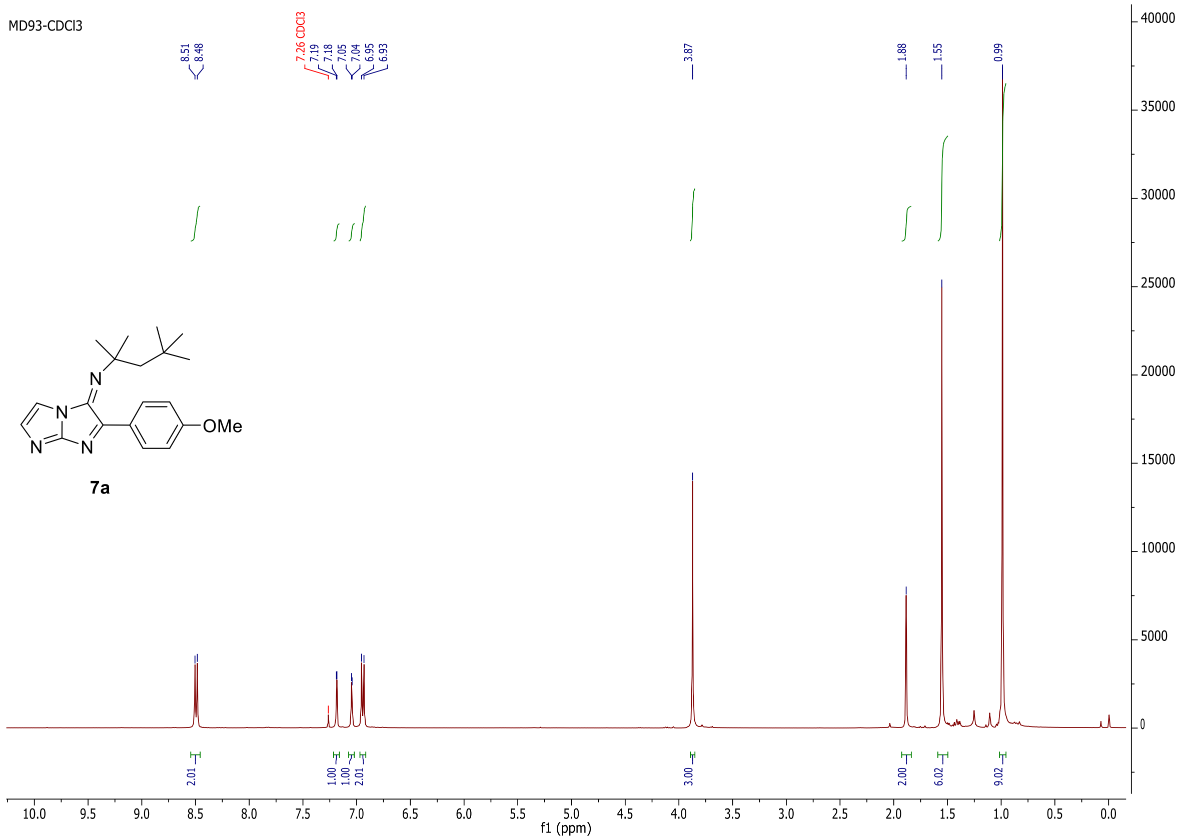
MD93-CDCl3
8.51
8.48
7.26 CDCl3
7.19
7.18
7.05
7.04
6.95
6.93
3.87
1.88
1.55
0.99
OMe
N
7a
2.01
1.00
1.00
2.01
3.00
2.00
6.02
9.02
f1 (ppm)

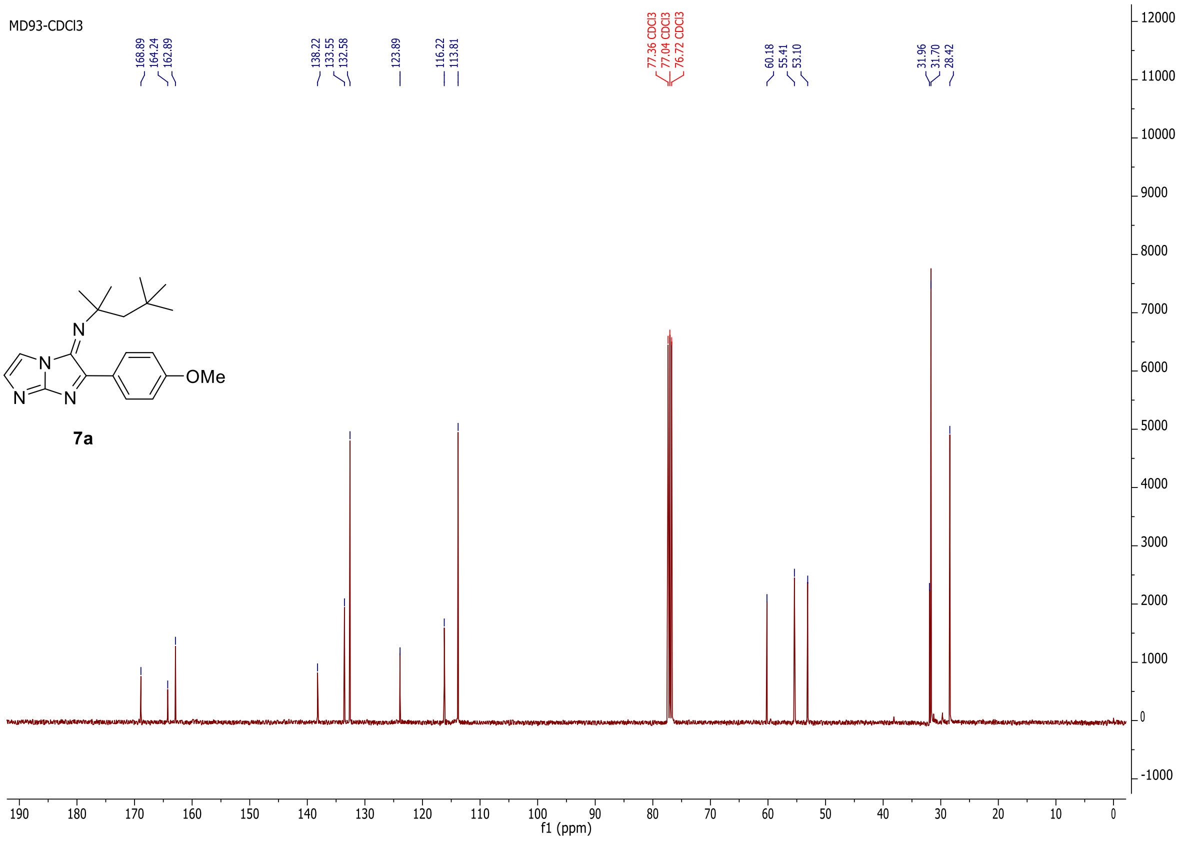
MD93-CDCl3
168.89
164.24
162.89
138.22
133.55
132.58
123.89
116.22
113.81
77.36 CDCl3
77.04 CDCl3
76.72 CDCl3
60.18
55.41
53.10
31.96
31.70
28.42
OMe
N
7a
f1 (ppm)

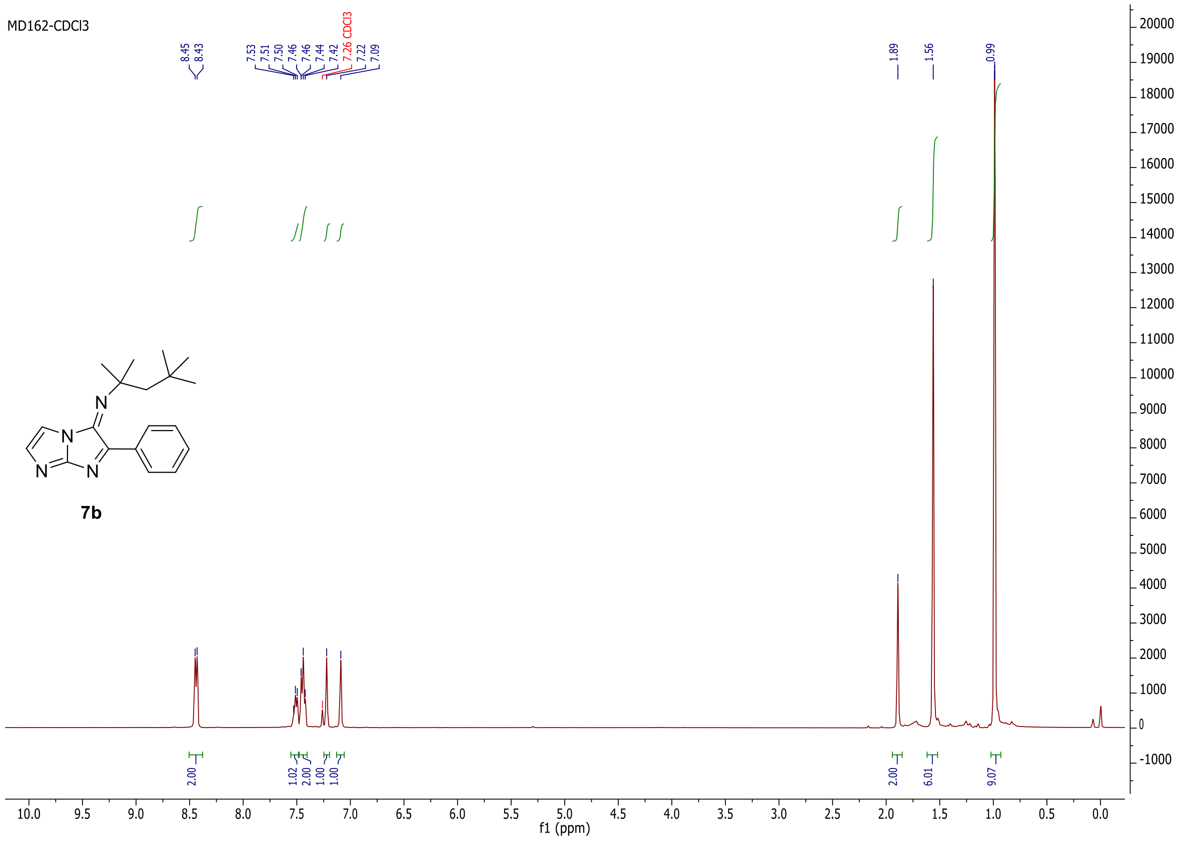
MD162-CDCl3
7b
f1 (ppm)

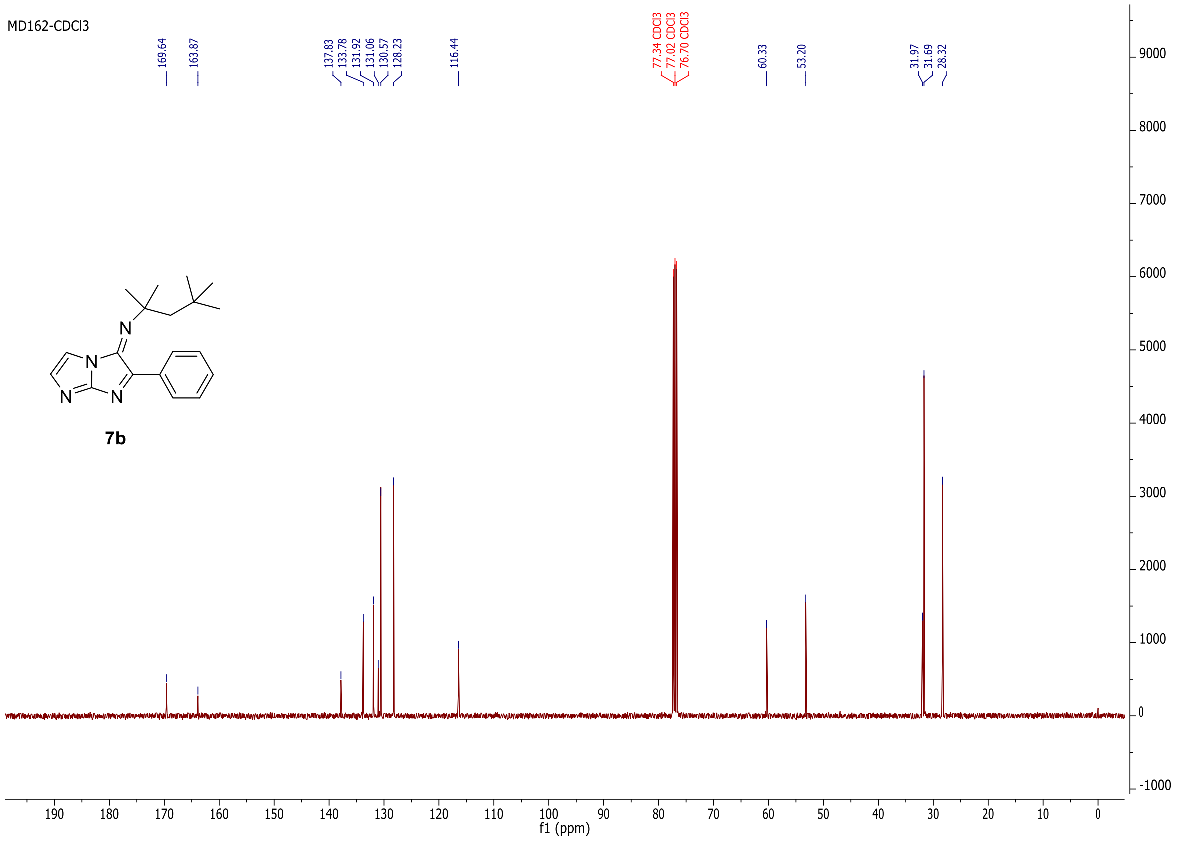
MD162-CDCl3
7b
f1 (ppm)

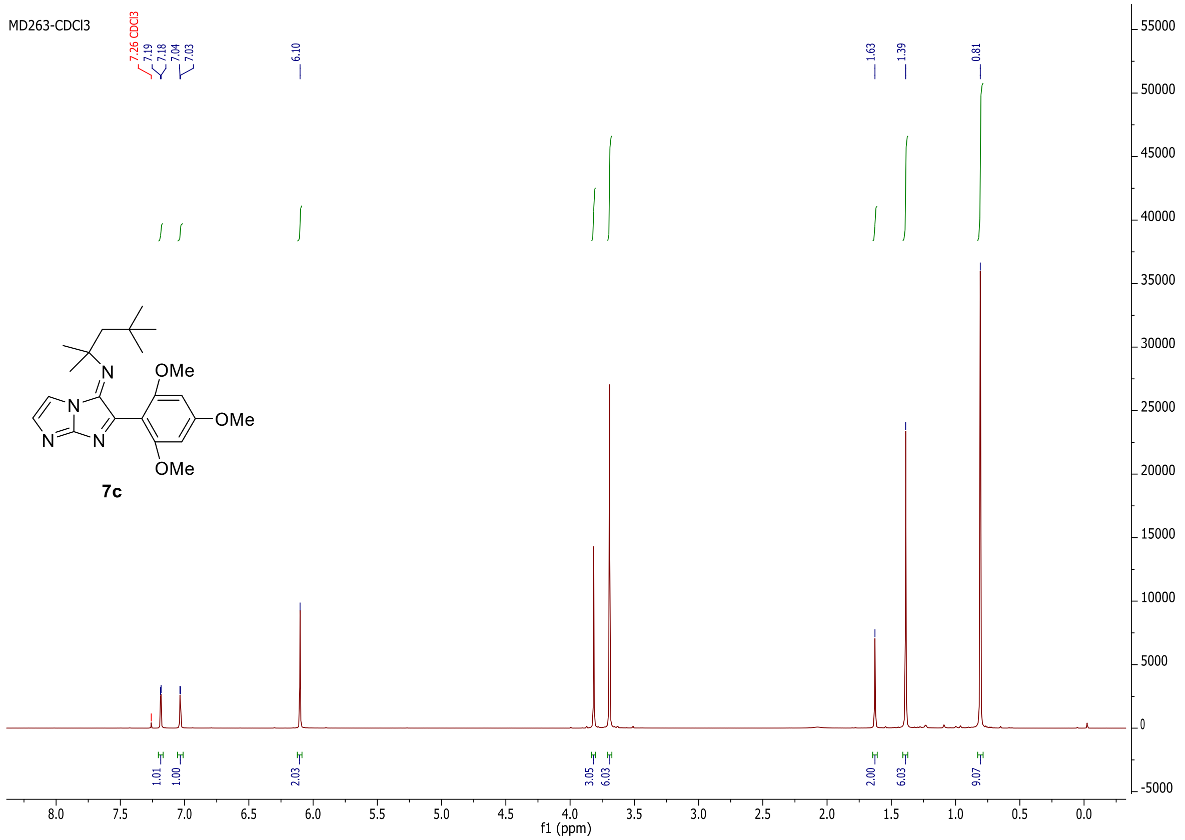
MD263-CDCl3
7.26 CDCl3
7.19
7.18
7.04
7.03
6.10
1.63
1.39
0.81
OMe
OMe
OMe
N
N
N
N
7c
1.01
1.00
2.03
3.05
6.03
2.00
6.03
9.07
f1 (ppm)

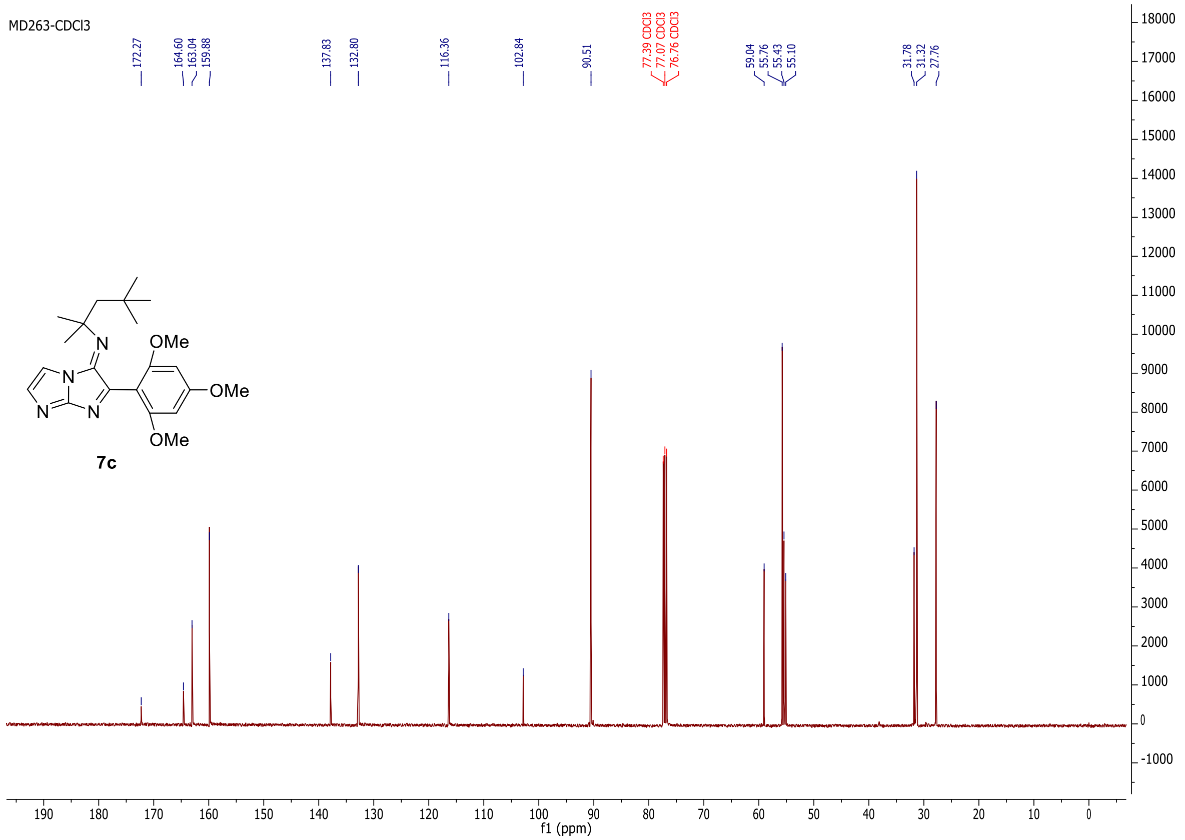
MD263-CDCl3
172.27
164.60
163.04
159.88
137.83
132.80
116.36
102.84
90.51
77.39 CDCl3
77.07 CDCl3
76.76 CDCl3
59.04
55.76
55.43
55.10
31.78
31.32
27.76
OMe
OMe
OMe
7c
f1 (ppm)

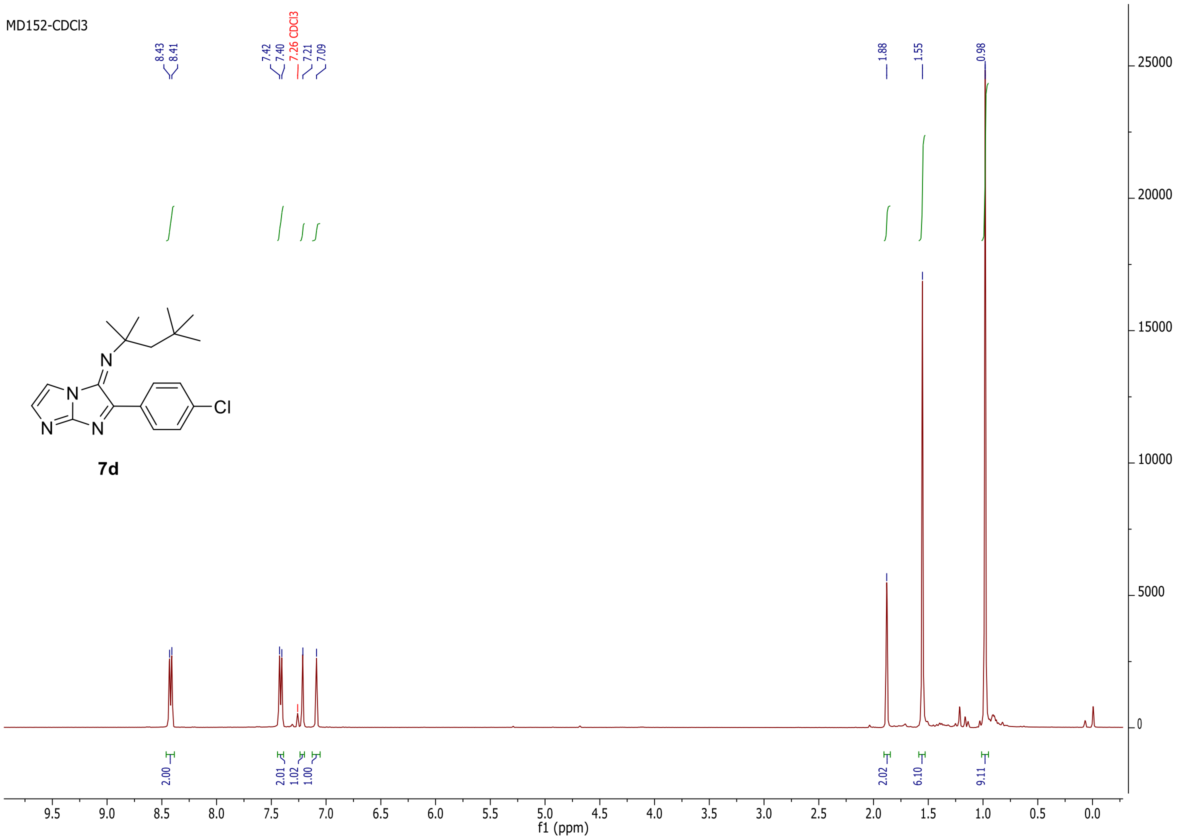
MD152-CDCl3
8.43
8.41
7.42
7.40
7.26 CDCl3
7.21
7.09
1.88
1.55
0.98
2.00
2.01
1.02
1.00
2.02
6.10
9.11
7d
f1 (ppm)

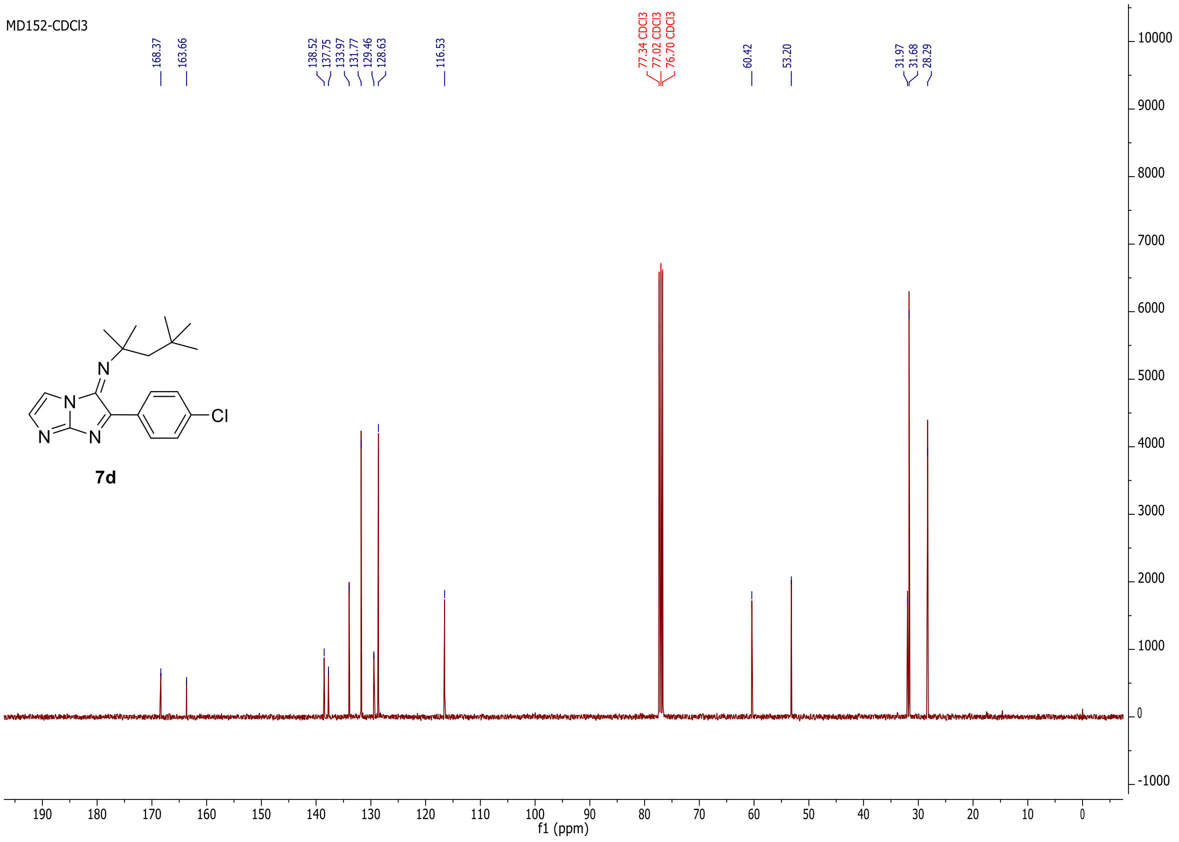
MD152-CDCl3
168.37
163.66
138.52
137.75
133.97
131.77
129.46
128.63
116.53
77.34 CDCl3
77.02 CDCl3
76.70 CDCl3
60.42
53.20
31.97
31.68
28.29
7d
f1 (ppm)

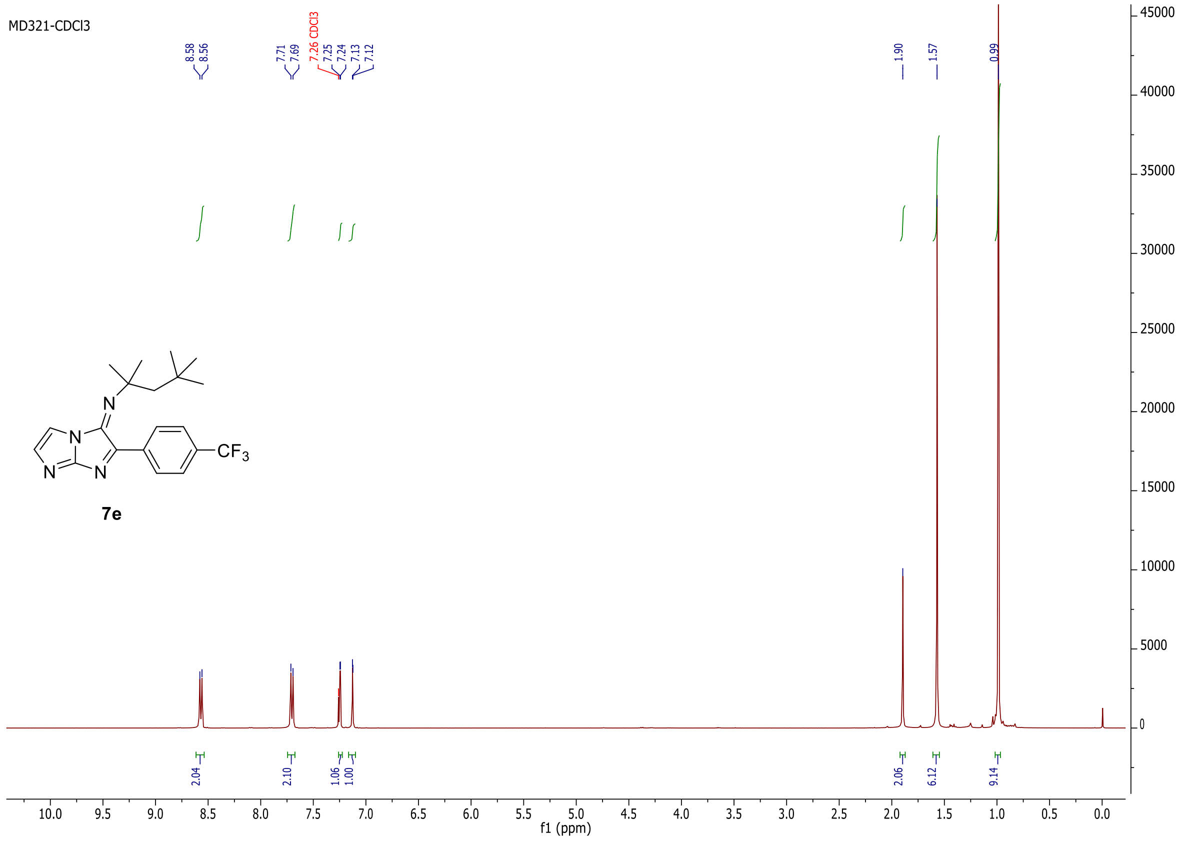

MD321-CDCl3
8.58
8.56
7.71
7.69
7.26 CDCl3
7.25
7.24
7.13
7.12
1.90
1.57
0.99
7e
2.04
2.10
1.06
1.00
2.06
6.12
9.14
f1 (ppm)

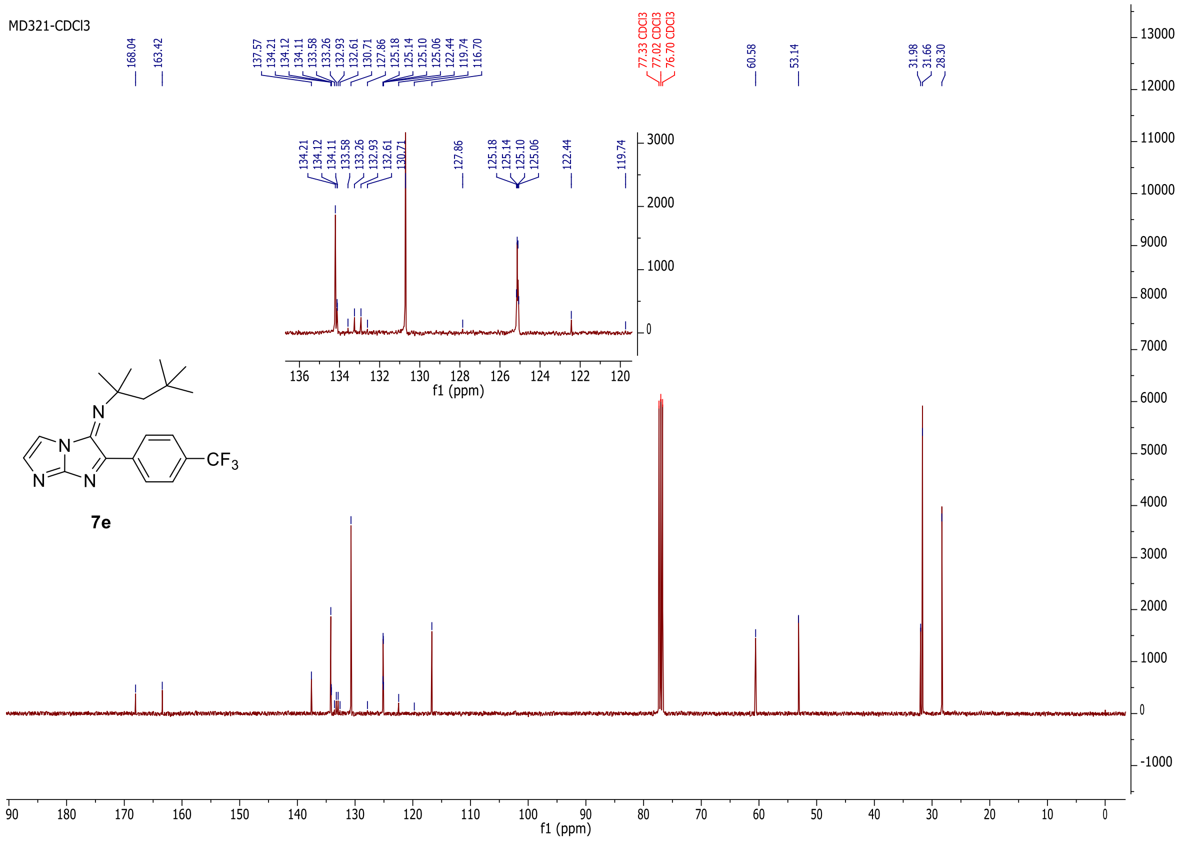

MD321-CDCl3
168.04
163.42
137.57
134.21
134.12
134.11
133.58
133.26
132.93
132.61
130.71
127.86
125.18
125.14
125.10
125.06
122.44
119.74
116.70
77.33 CDCl3
77.02 CDCl3
76.70 CDCl3
60.58
53.14
31.98
31.66
28.30
7e
f1 (ppm)

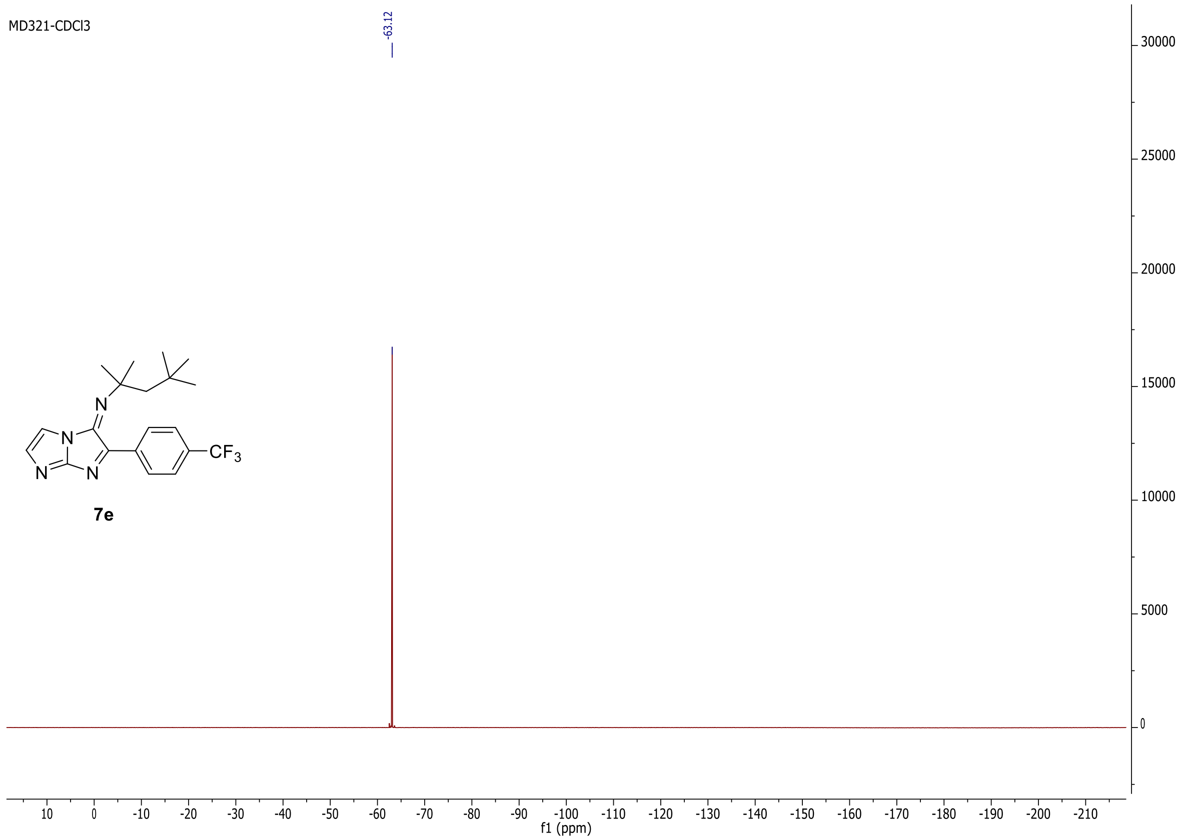

MD321-CDCl3
-63.12
CF3
N
N
N
N
7e
30000
25000
20000
15000
10000
5000
0
10 0 -10 -20 -30 -40 -50 -60 -70 -80 -90 -100 -110 -120 -130 -140 -150 -160 -170 -180 -190 -200 -210
f1 (ppm)

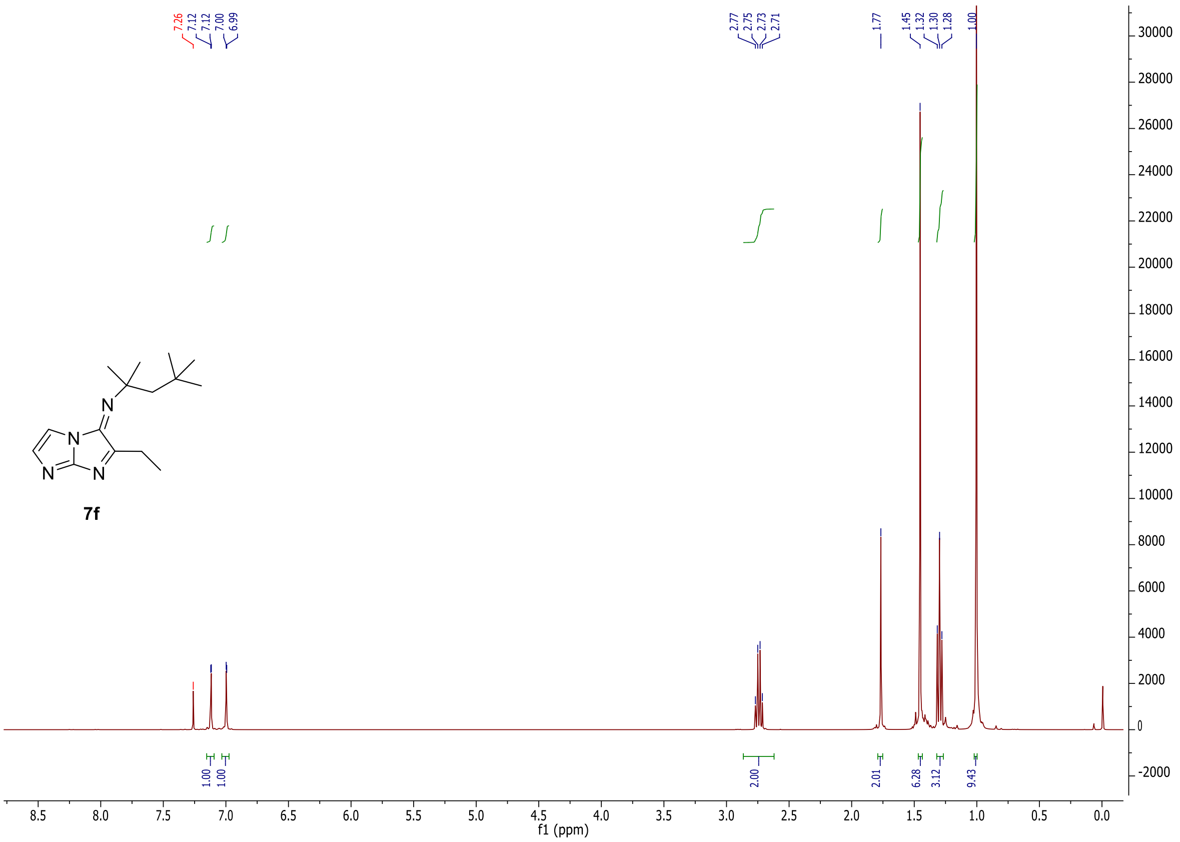

7.26
7.12
7.12
7.00
6.99
2.77
2.75
2.73
2.71
1.77
1.45
1.32
1.30
1.28
1.00
7f
1.00
1.00
2.00
2.01
6.28
3.12
9.43
f1 (ppm)

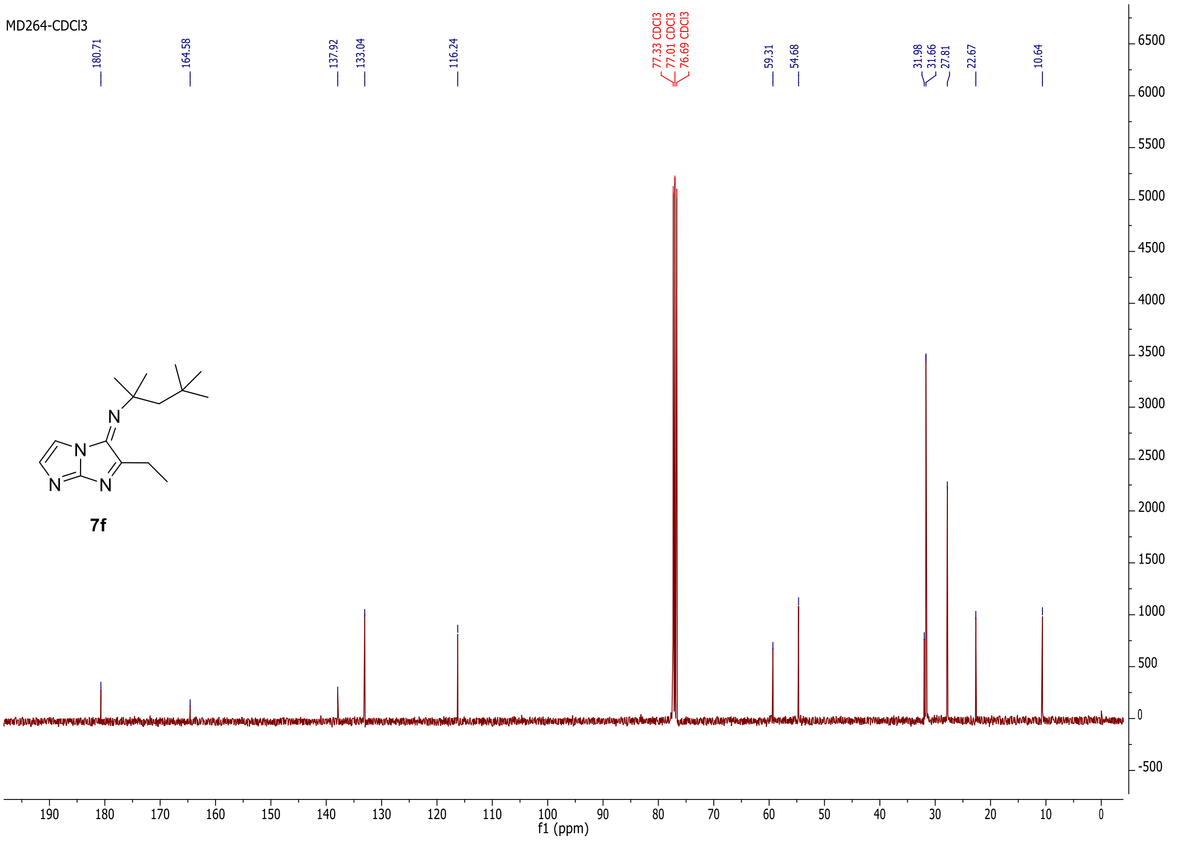

MD264-CDCl3
180.71
164.58
137.92
133.04
116.24
77.33 CDCl3
77.01 CDCl3
76.69 CDCl3
59.31
54.68
31.98
31.66
27.81
22.67
10.64
7f
f1 (ppm)

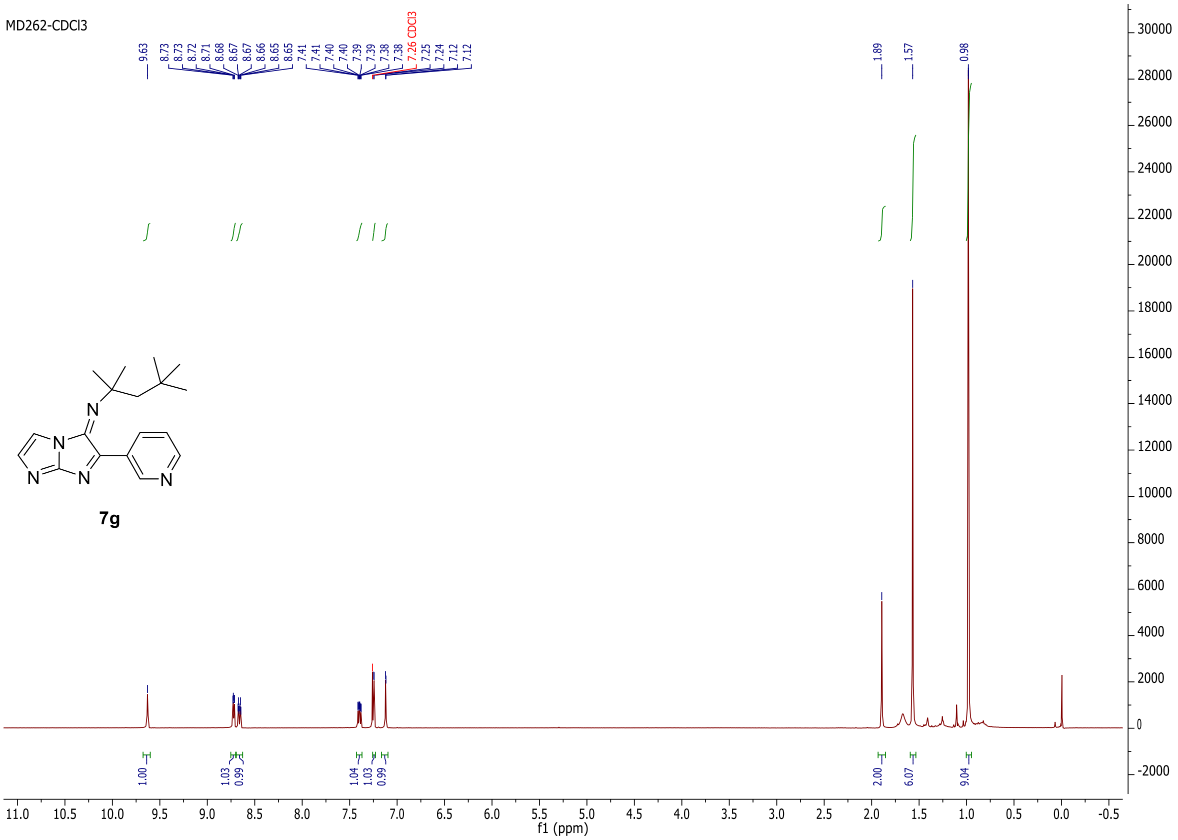
MD262-CDCl3
9.63
8.73 8.73 8.72 8.71 8.68 8.67 8.67 8.66 8.65 8.65
7.41 7.41 7.40 7.40 7.39 7.39 7.38 7.38
7.26 CDCl3
7.25 7.24 7.12 7.12
1.89
1.57
0.98
N
N
N
N
N
7g
1.00
1.03
0.99
1.04
1.03
0.99
2.00
6.07
9.04
f1 (ppm)

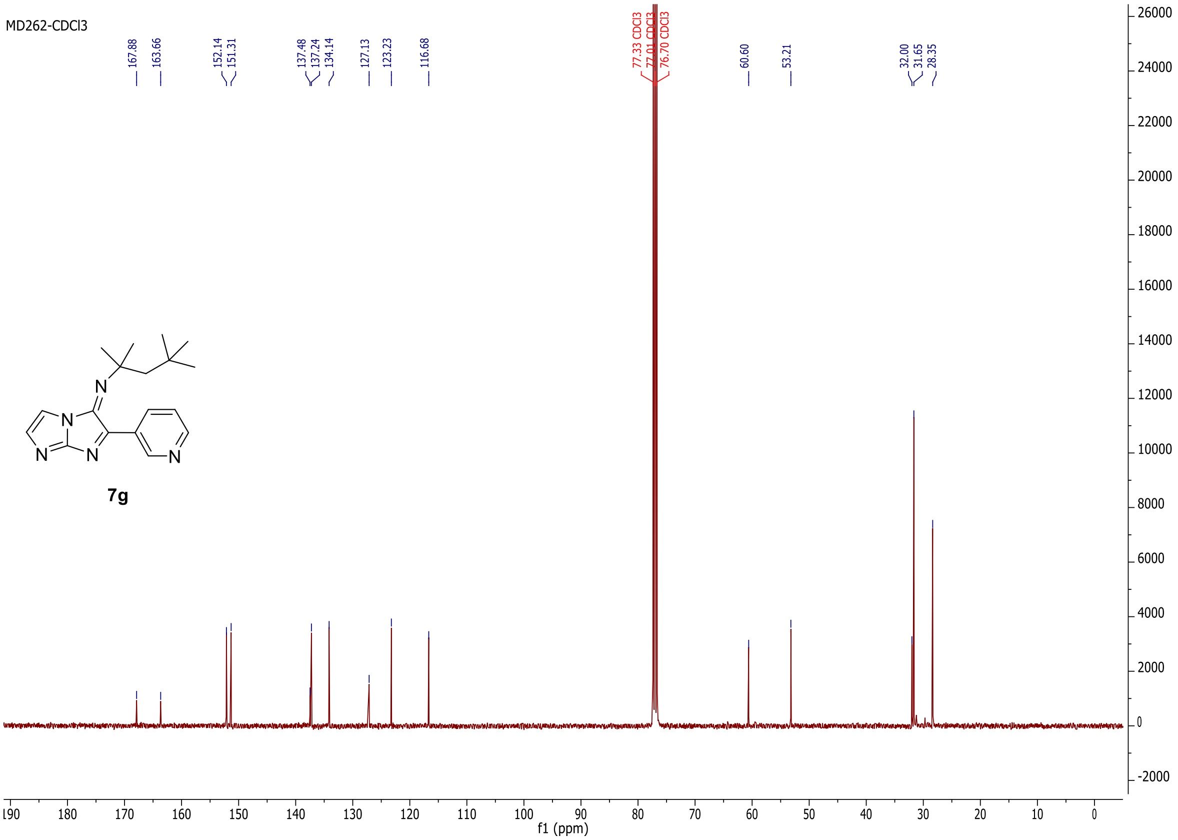
MD262-CDCl3
167.88
163.66
152.14
151.31
137.48
137.24
134.14
127.13
123.23
116.68
77.33 CDCl3
77.01 CDCl3
76.70 CDCl3
60.60
53.21
32.00
31.65
28.35
N
N
N
N
N
7g
f1 (ppm)

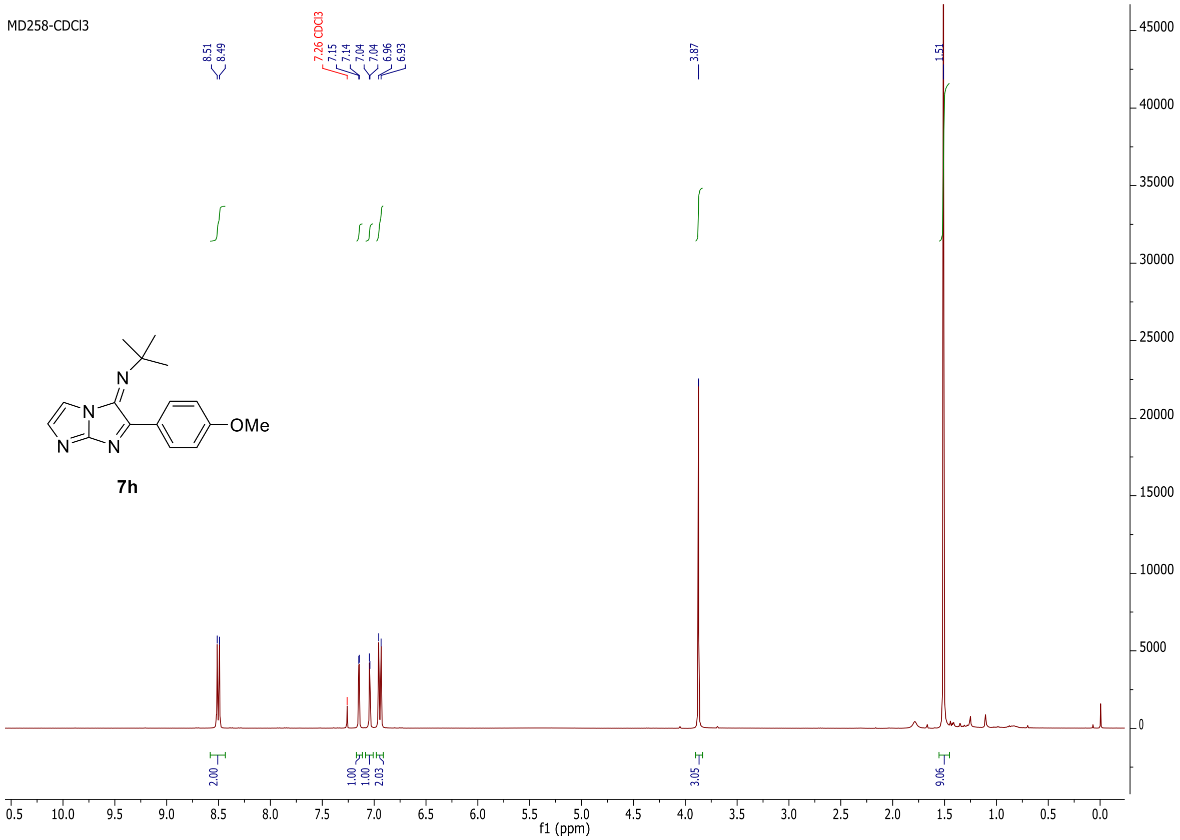
MD258-CDCl3
8.51
8.49
7.26 CDCl3
7.15
7.14
7.04
7.04
6.96
6.93
3.87
1.51
OMe
7h
2.00
1.00
1.00
2.03
3.05
9.06
f1 (ppm)

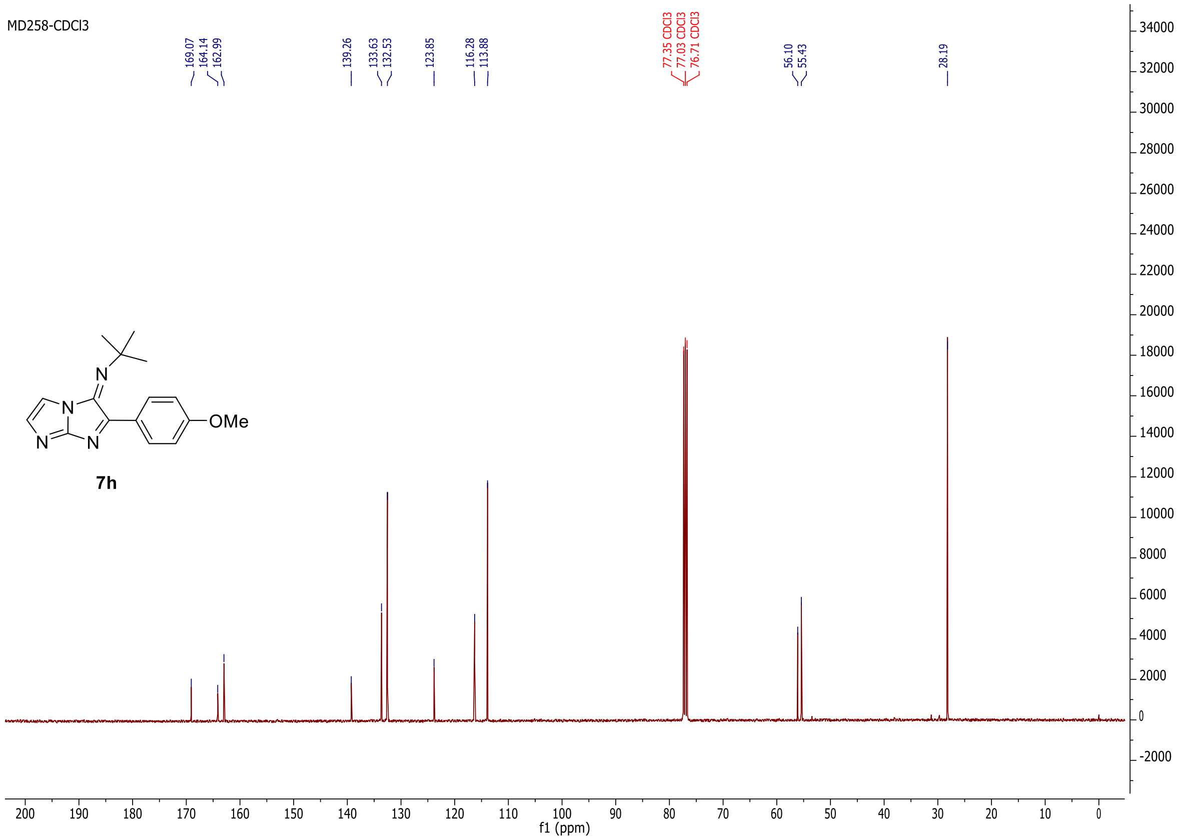
MD258-CDCl3
169.07
164.14
162.99
139.26
133.63
132.53
123.85
116.28
113.88
77.35 CDCl3
77.03 CDCl3
76.71 CDCl3
56.10
55.43
28.19
OMe
7h
f1 (ppm)

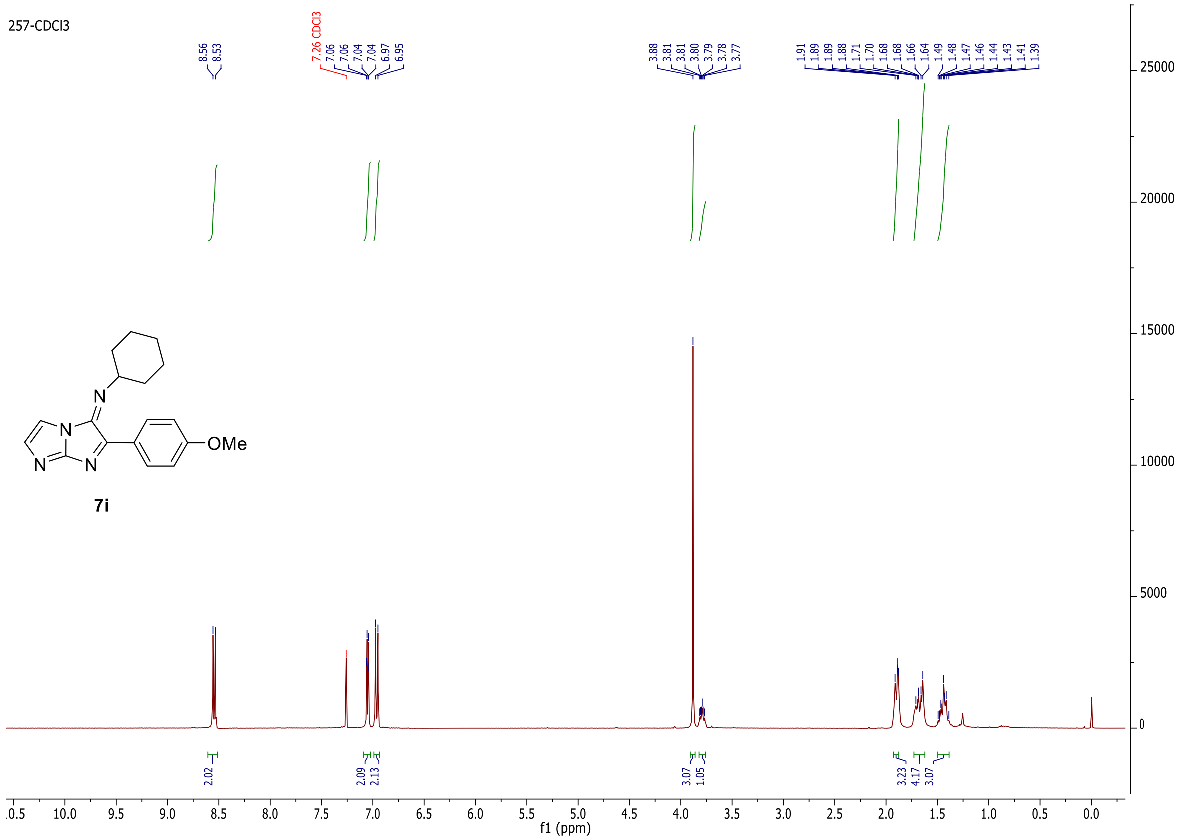
257-CDCl3
7i
OMe
f1 (ppm)

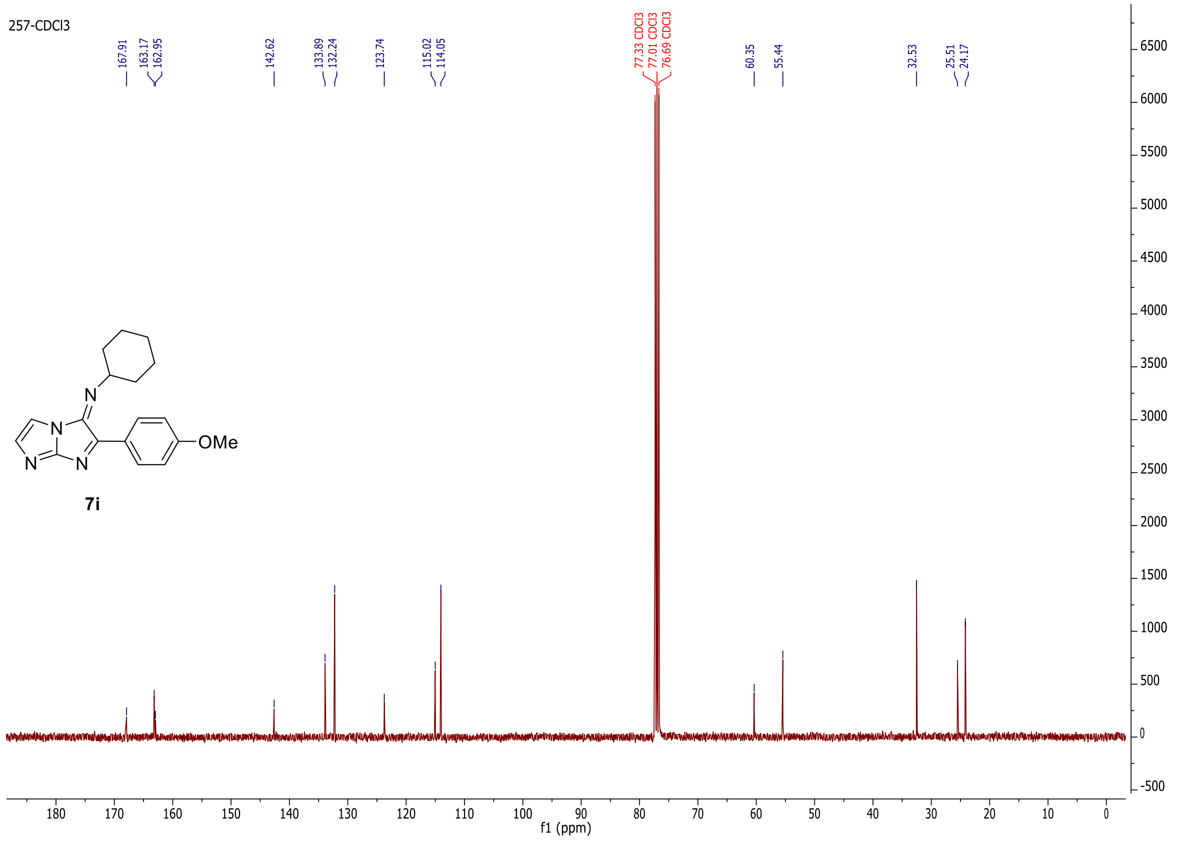
257-CDCl3
7i
OMe
f1 (ppm)

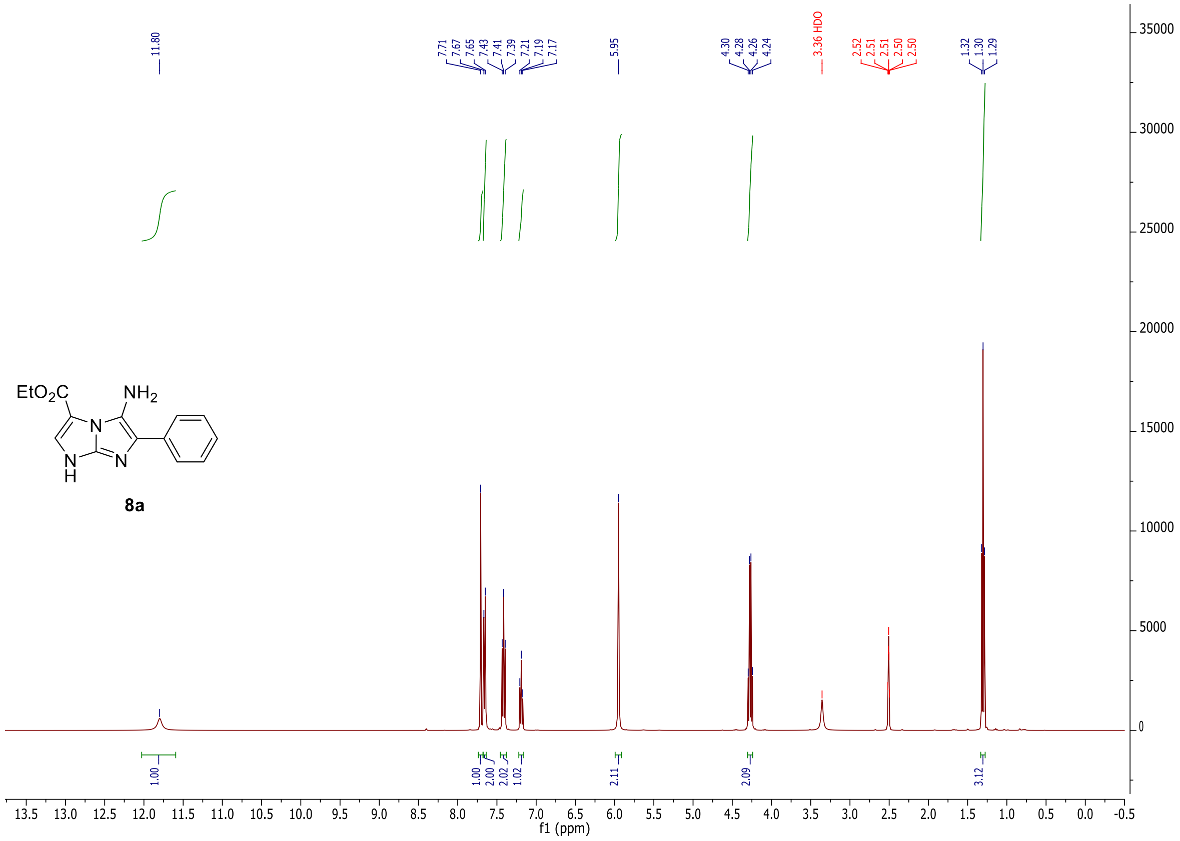
11.80
7.71
7.67
7.65
7.43
7.41
7.39
7.21
7.19
7.17
5.95
4.30
4.28
4.26
4.24
3.36 HDO
2.52
2.51
2.51
2.50
2.50
1.32
1.30
1.29
EtO2C
NH2
N
N
H
N
8a
1.00
1.00
2.00
2.02
1.02
2.11
2.09
3.12
f1 (ppm)

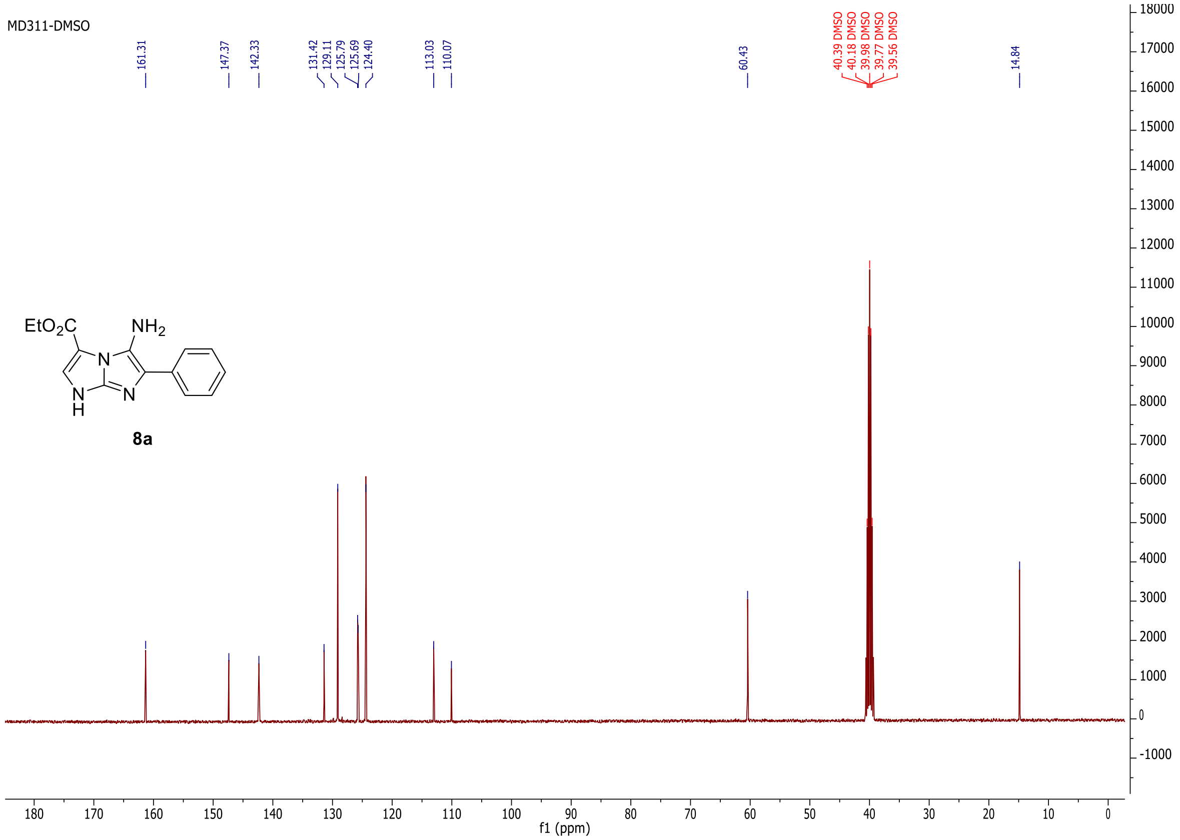
MD311-DMSO
161.31
147.37
142.33
131.42
129.11
125.79
125.69
124.40
113.03
110.07
60.43
40.39 DMSO
40.18 DMSO
39.98 DMSO
39.77 DMSO
39.56 DMSO
14.84
EtO2C
NH2
N
N
H
N
8a
f1 (ppm)

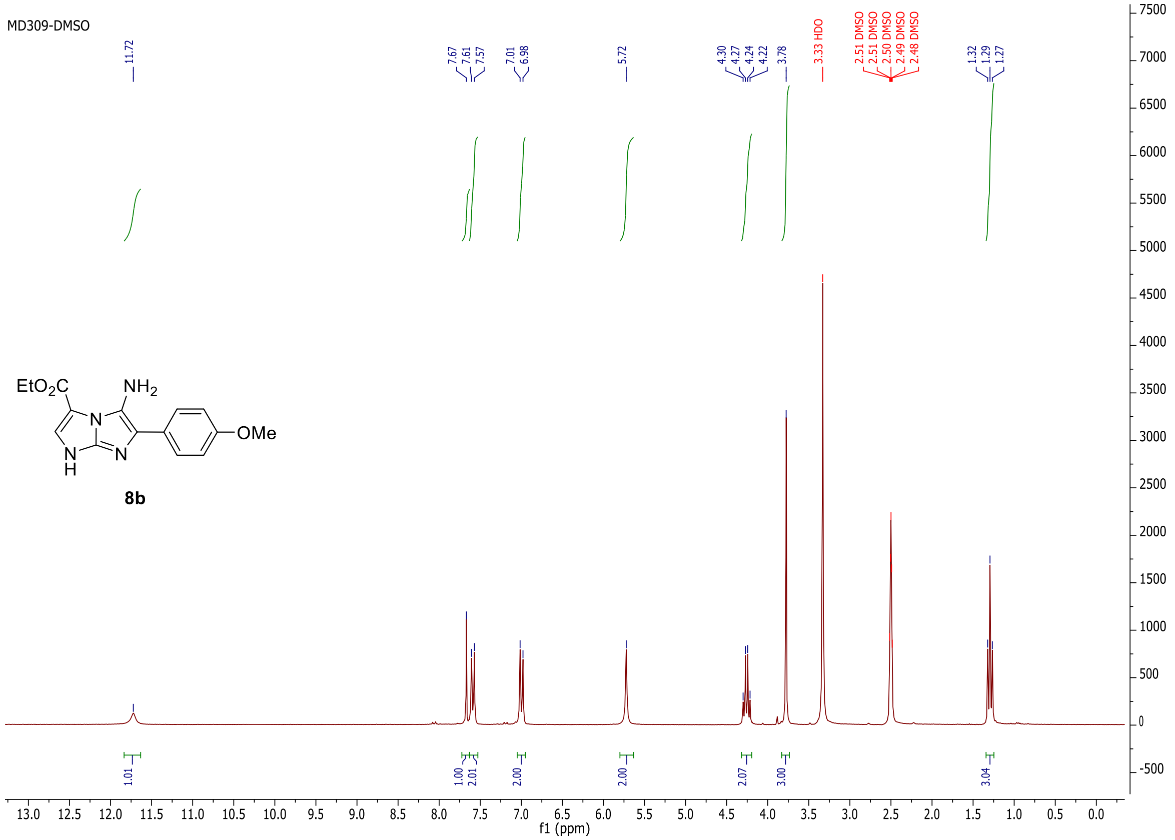
MD309-DMSO
11.72
7.67
7.61
7.57
7.01
6.98
5.72
4.30
4.27
4.24
4.22
3.78
3.33 HDO
2.51 DMSO
2.51 DMSO
2.50 DMSO
2.49 DMSO
2.48 DMSO
1.32
1.29
1.27
EtO2C
NH2
N
N
N
H
OMe
8b
1.01
1.00
2.01
2.00
2.00
2.07
3.00
3.04
f1 (ppm)

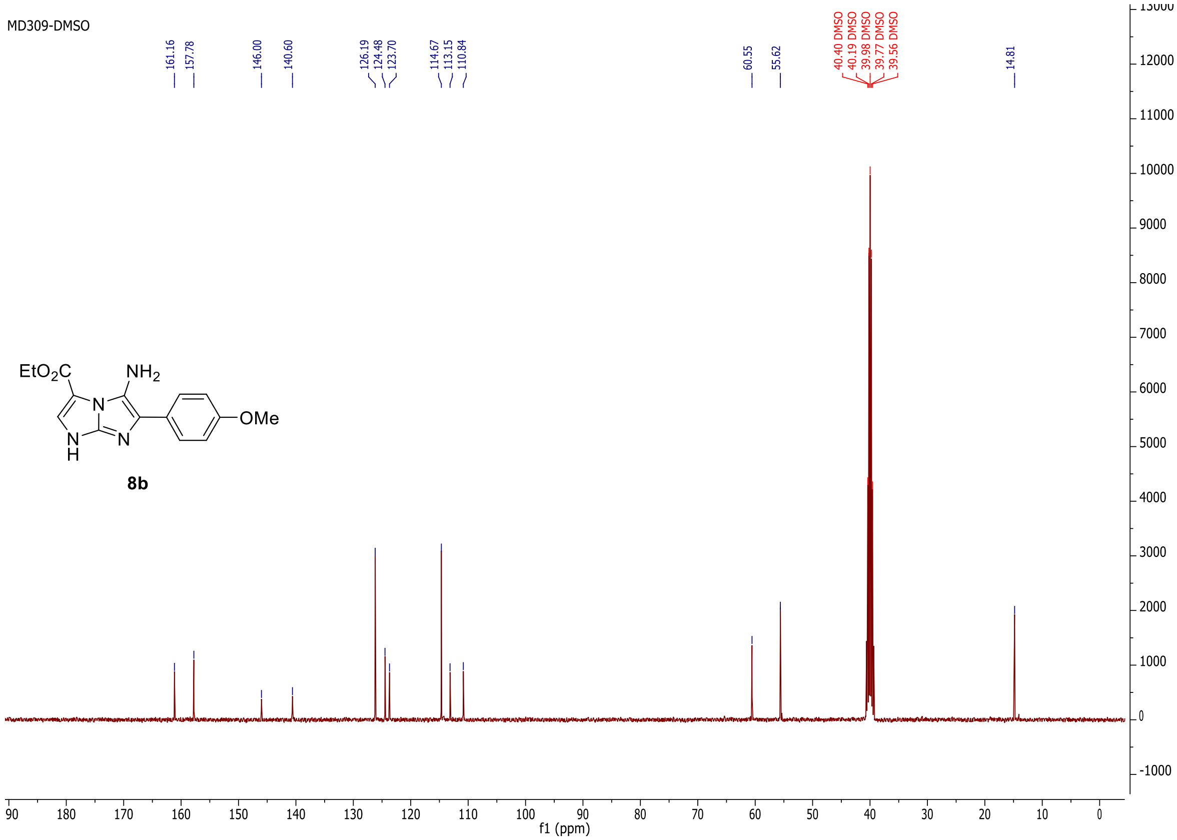
MD309-DMSO
161.16
157.78
146.00
140.60
126.19
124.48
123.70
114.67
113.15
110.84
60.55
55.62
40.40 DMSO
40.19 DMSO
39.98 DMSO
39.77 DMSO
39.56 DMSO
14.81
EtO2C
NH2
N
N
N
H
OMe
8b
f1 (ppm)

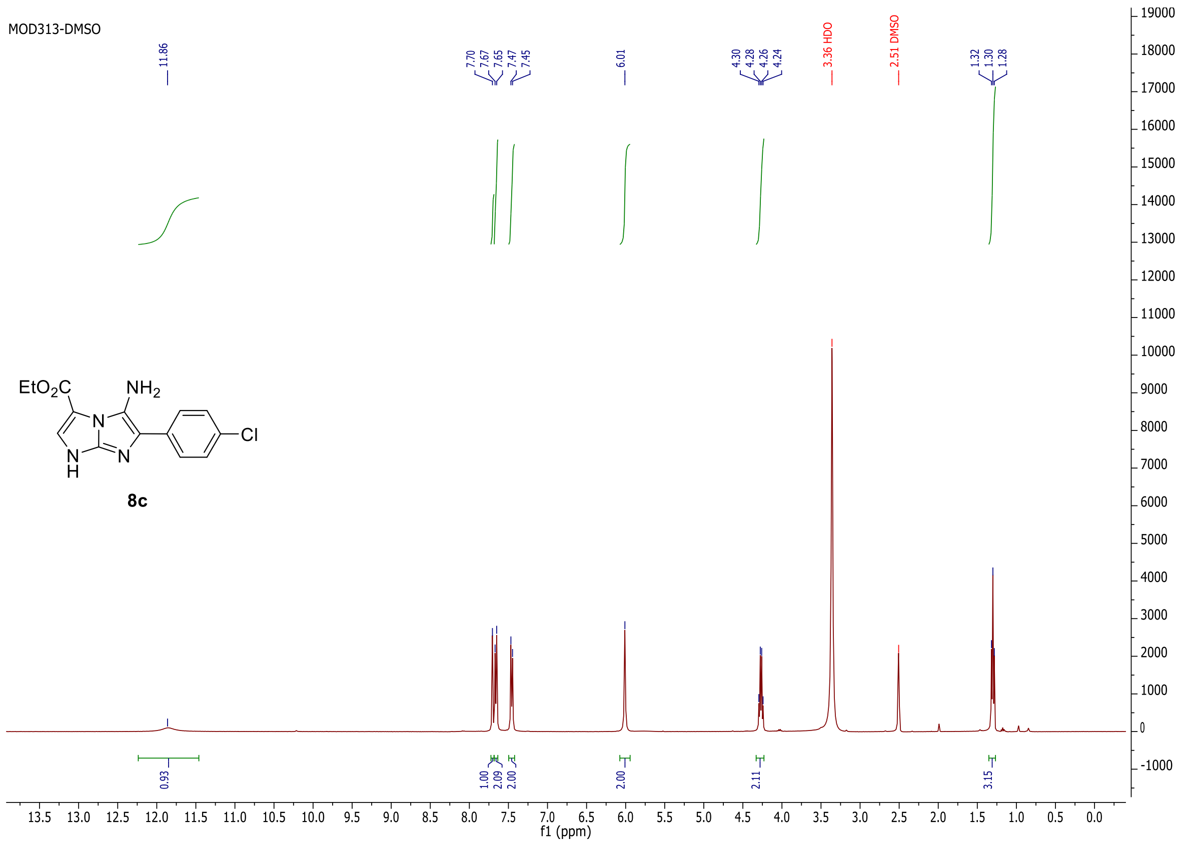
MOD313-DMSO
11.86
7.70
7.67
7.65
7.47
7.45
6.01
4.30
4.28
4.26
4.24
3.36 HDO
2.51 DMSO
1.32
1.30
1.28
0.93
1.00
2.09
2.00
2.00
2.11
3.15
f1 (ppm)
EtO2C
NH2
N
H
Cl
8c

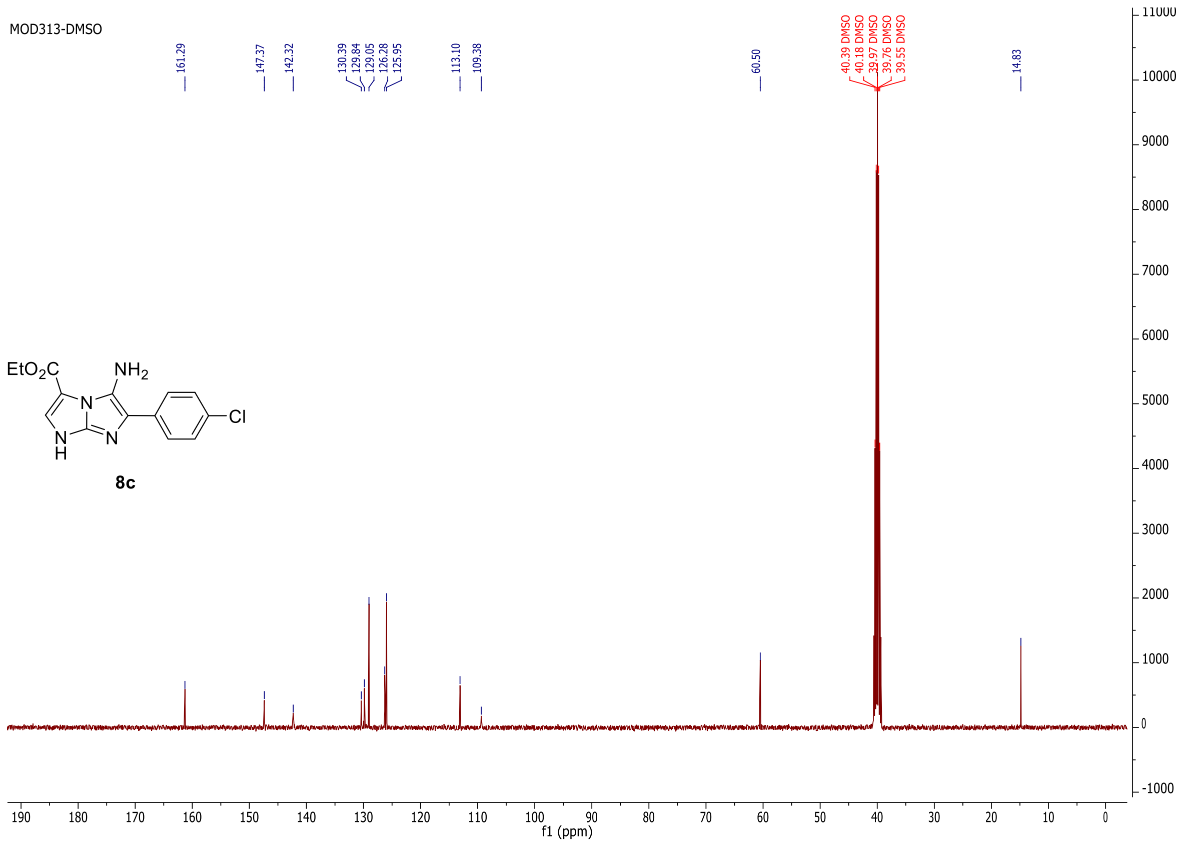
MOD313-DMSO
161.29
147.37
142.32
130.39
129.84
129.05
126.28
125.95
113.10
109.38
60.50
40.39 DMSO
40.18 DMSO
39.97 DMSO
39.76 DMSO
39.55 DMSO
14.83
f1 (ppm)
EtO2C
NH2
N
H
Cl
8c

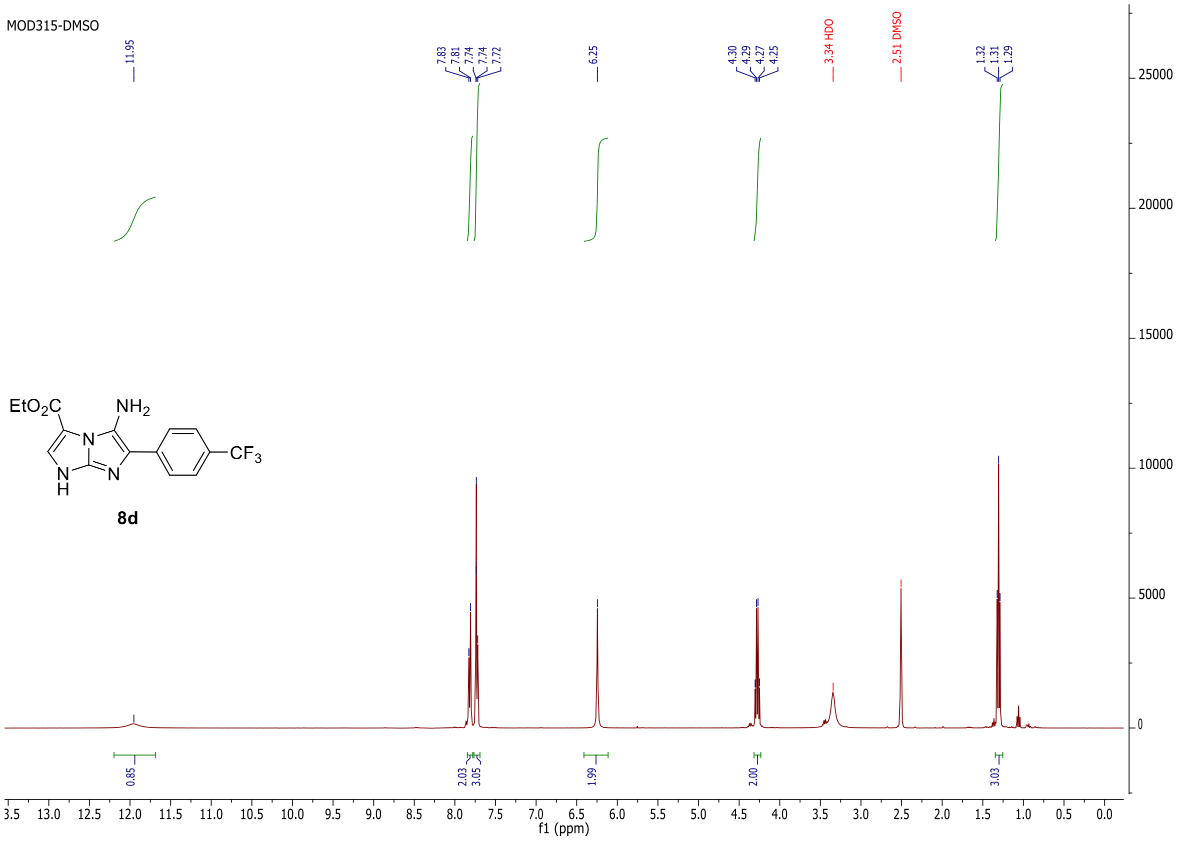
MOD315-DMSO
8d
f1 (ppm)

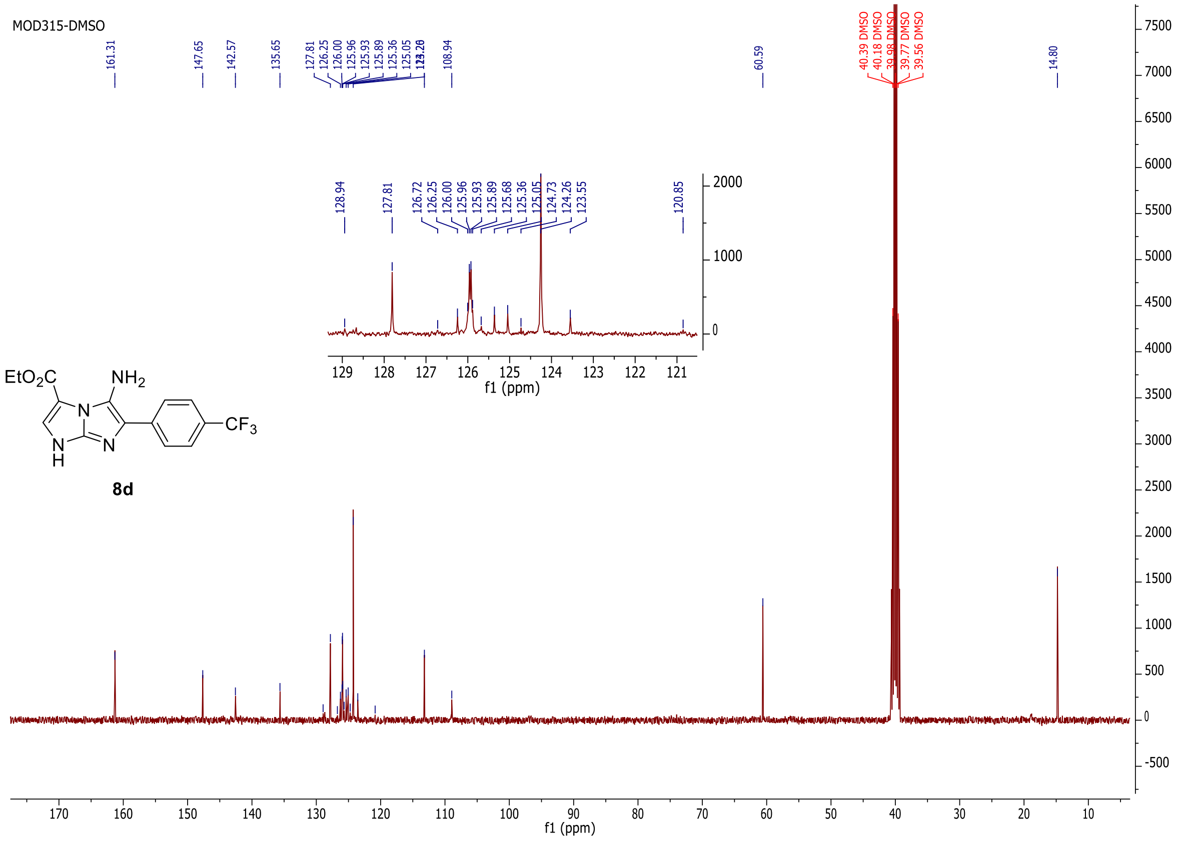
MOD315-DMSO
8d
f1 (ppm)

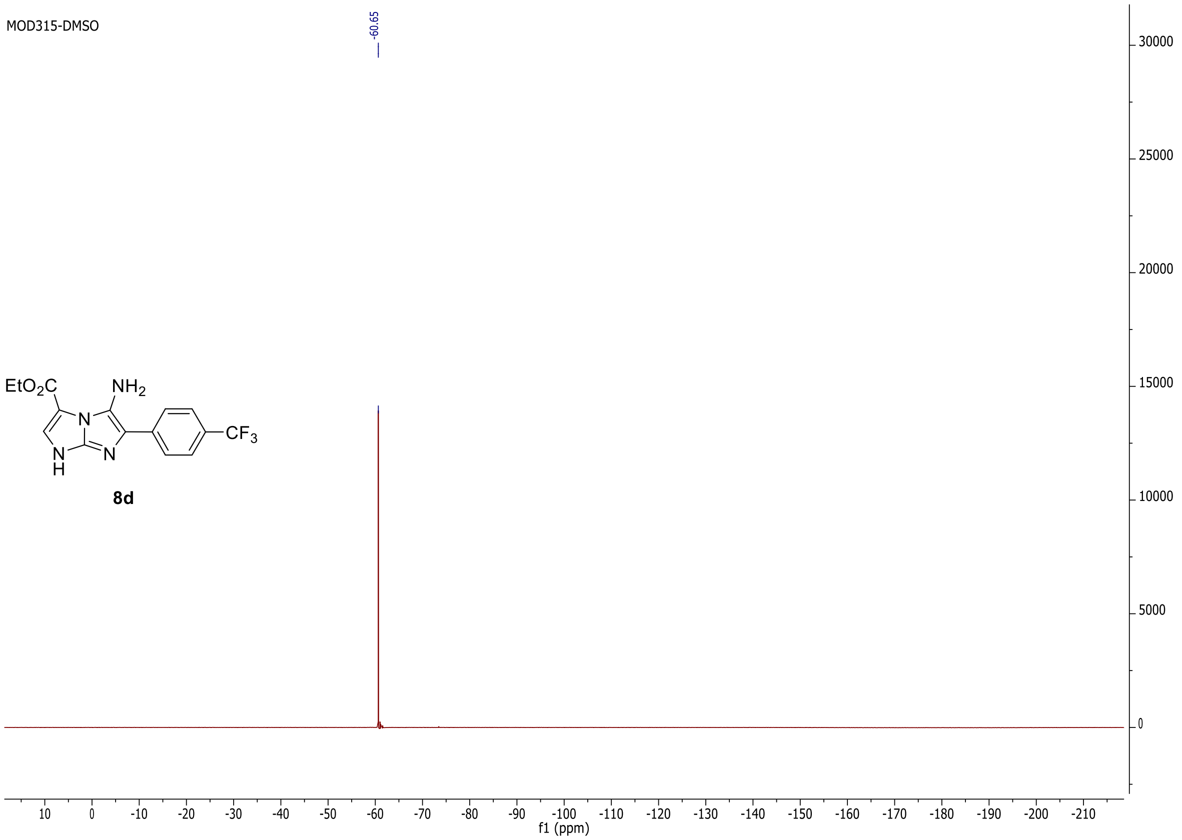
MOD315-DMSO
-60.65
EtO2C
NH2
N
N
N
H
CF3
30000
25000
20000
15000
10000
5000
0
10 0 -10 -20 -30 -40 -50 -60 -70 -80 -90 -100 -110 -120 -130 -140 -150 -160 -170 -180 -190 -200 -210
f1 (ppm)

8d

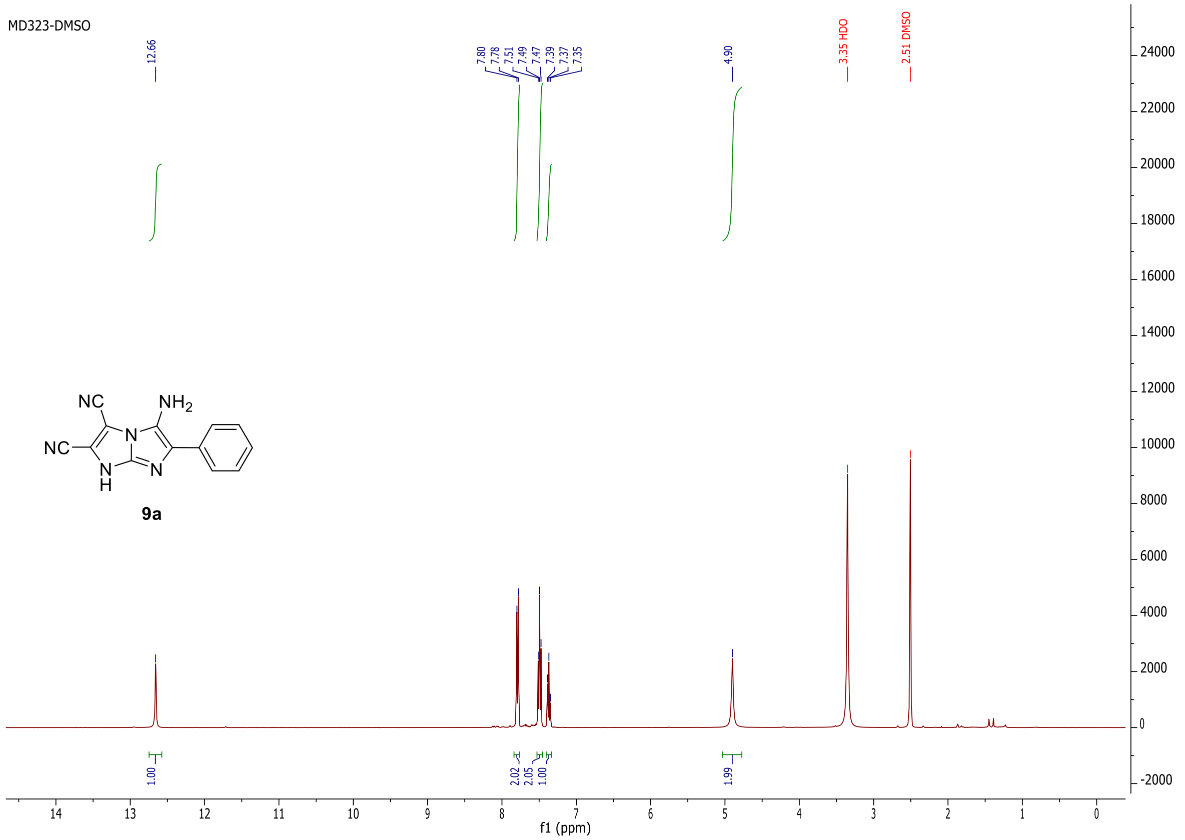
MD323-DMSO
12.66
7.80
7.78
7.51
7.49
7.47
7.39
7.37
7.35
4.90
3.35 HDO
2.51 DMSO
NC
NH2
NC
N
N
H
N
9a
1.00
2.02
2.05
1.00
1.99
f1 (ppm)

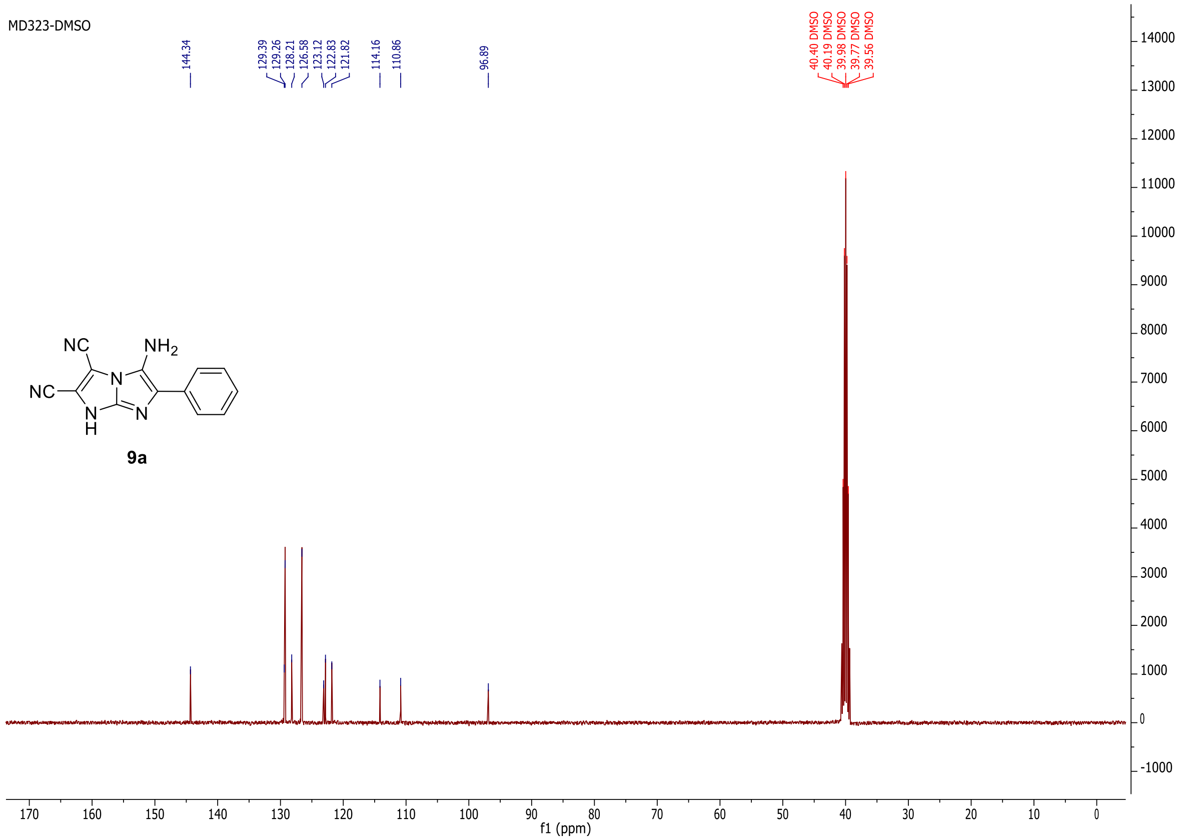
MD323-DMSO
144.34
129.39
129.26
128.21
126.58
123.12
122.83
121.82
114.16
110.86
96.89
40.40 DMSO
40.19 DMSO
39.98 DMSO
39.77 DMSO
39.56 DMSO
NC
NH2
NC
N
N
H
N
9a
f1 (ppm)

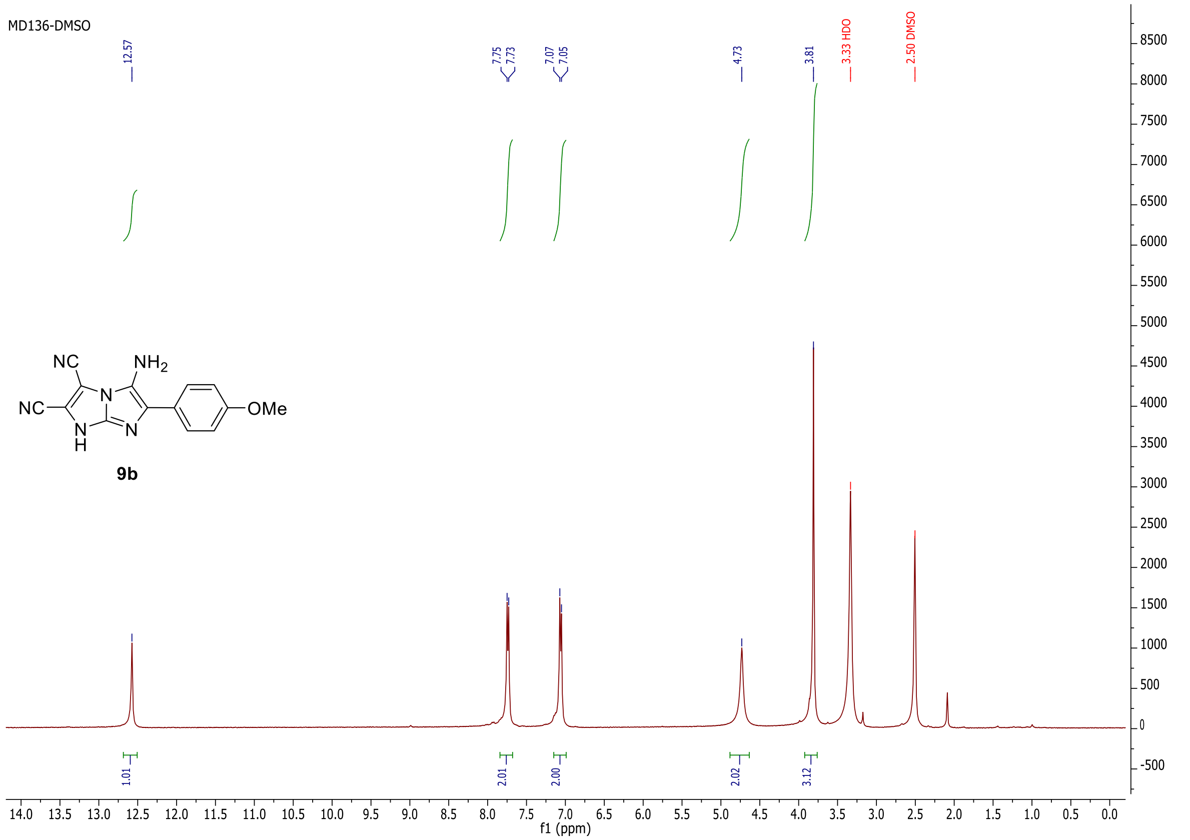
MD136-DMSO
12.57
7.75
7.73
7.07
7.05
4.73
3.81
3.33 HDO
2.50 DMSO
NC
NH2
NC
N
N
OMe
H
N
9b
1.01
2.01
2.00
2.02
3.12
f1 (ppm)

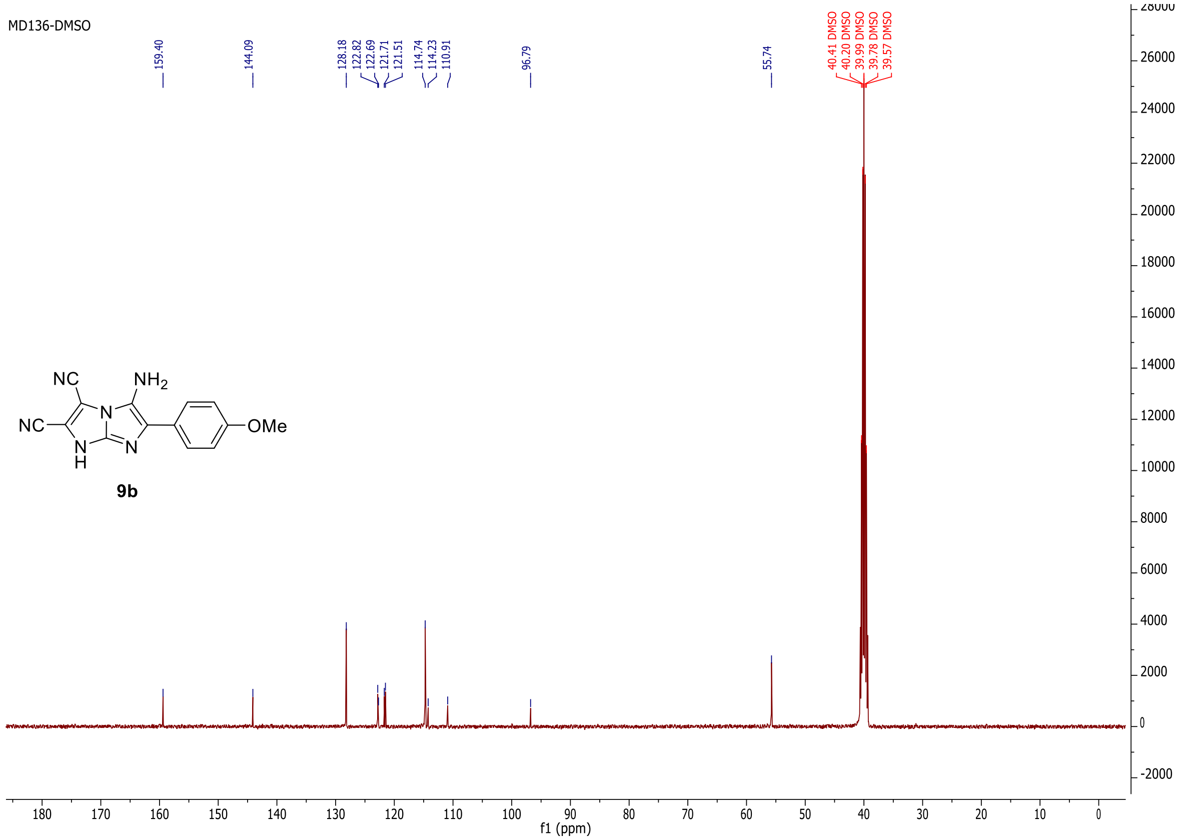
MD136-DMSO
159.40
144.09
128.18
122.82
122.69
121.71
121.51
114.74
114.23
110.91
96.79
55.74
40.41 DMSO
40.20 DMSO
39.99 DMSO
39.78 DMSO
39.57 DMSO
NC
NH2
NC
N
N
OMe
H
N
9b
f1 (ppm)

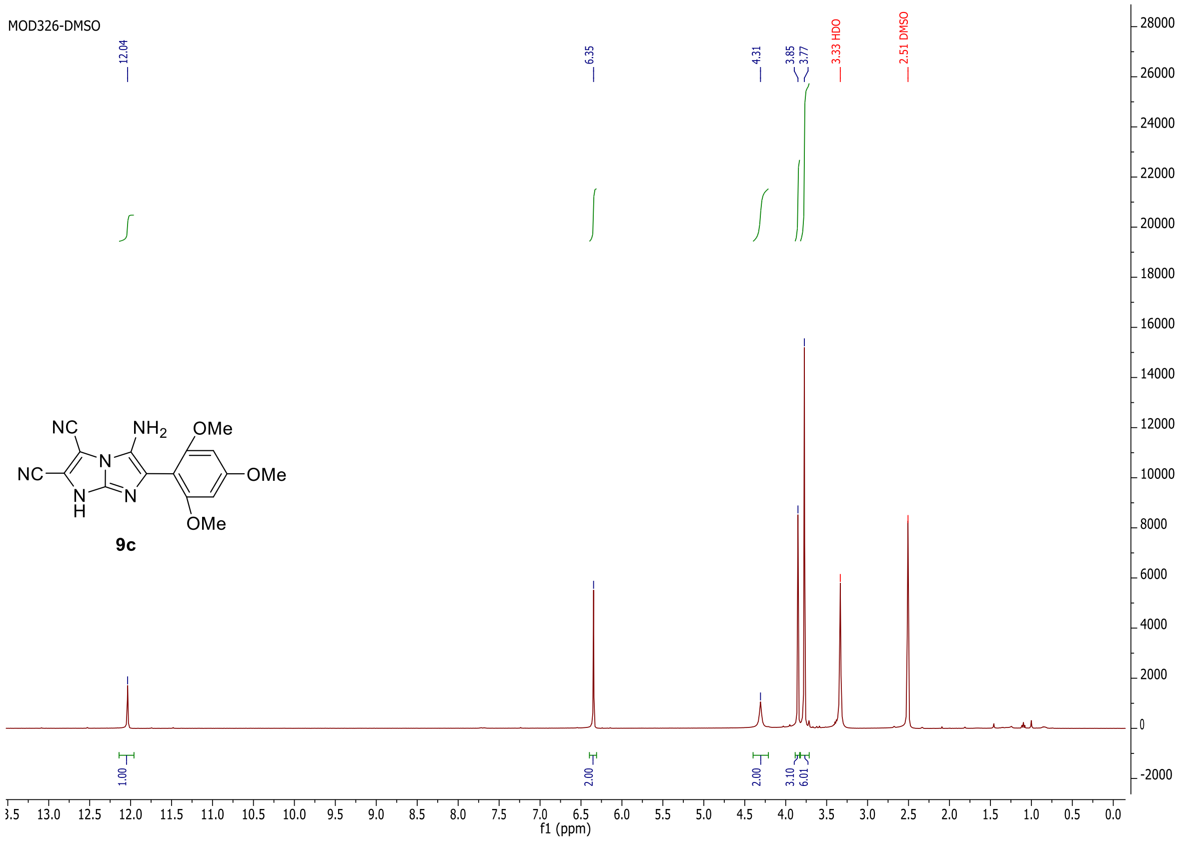
MOD326-DMSO
12.04
6.35
4.31
3.85
3.77
3.33 HDO
2.51 DMSO
NC
NH2
OMe
NC
OMe
N
H
N
N
OMe
9c
1.00
2.00
2.00
3.10
6.01
f1 (ppm)

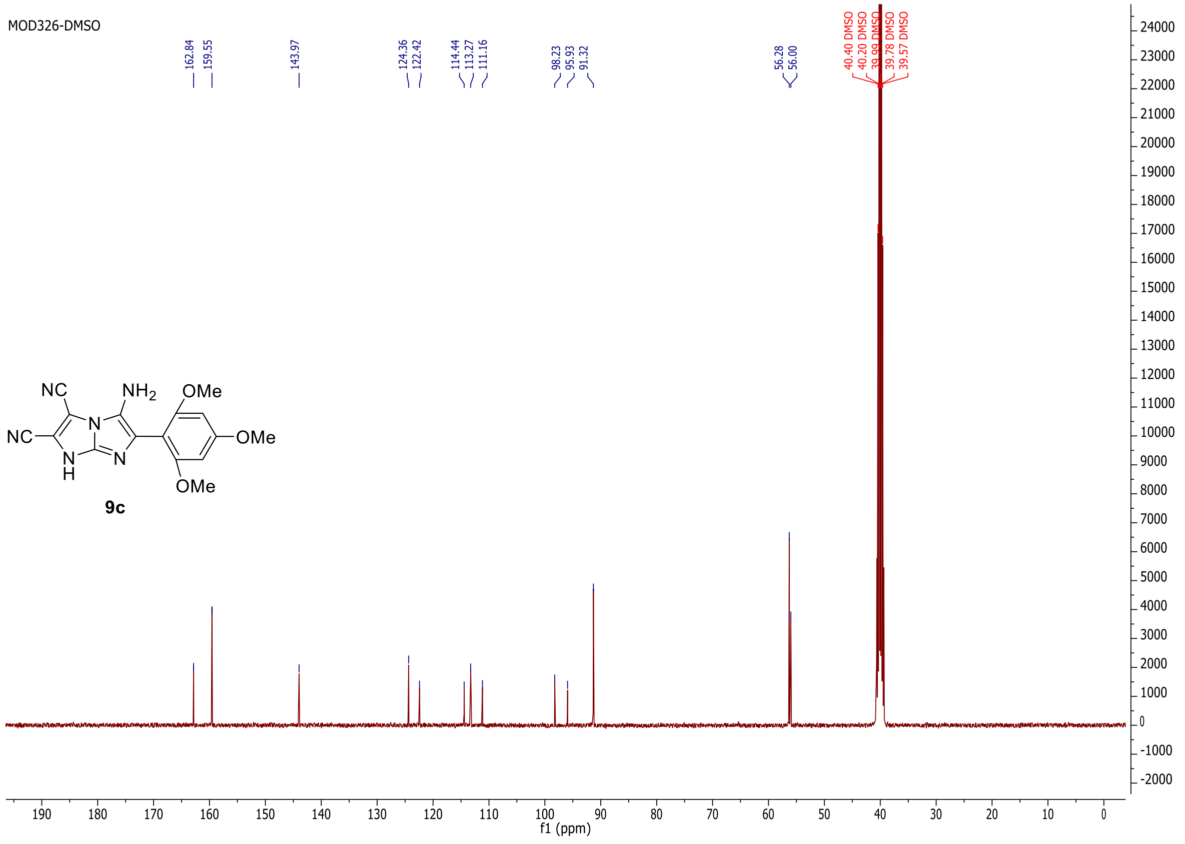
MOD326-DMSO
162.84
159.55
143.97
124.36
122.42
114.44
113.27
111.16
98.23
95.93
91.32
56.28
56.00
40.40 DMSO
40.20 DMSO
39.99 DMSO
39.78 DMSO
39.57 DMSO
NC
NH2
OMe
NC
OMe
N
H
N
N
OMe
9c
f1 (ppm)

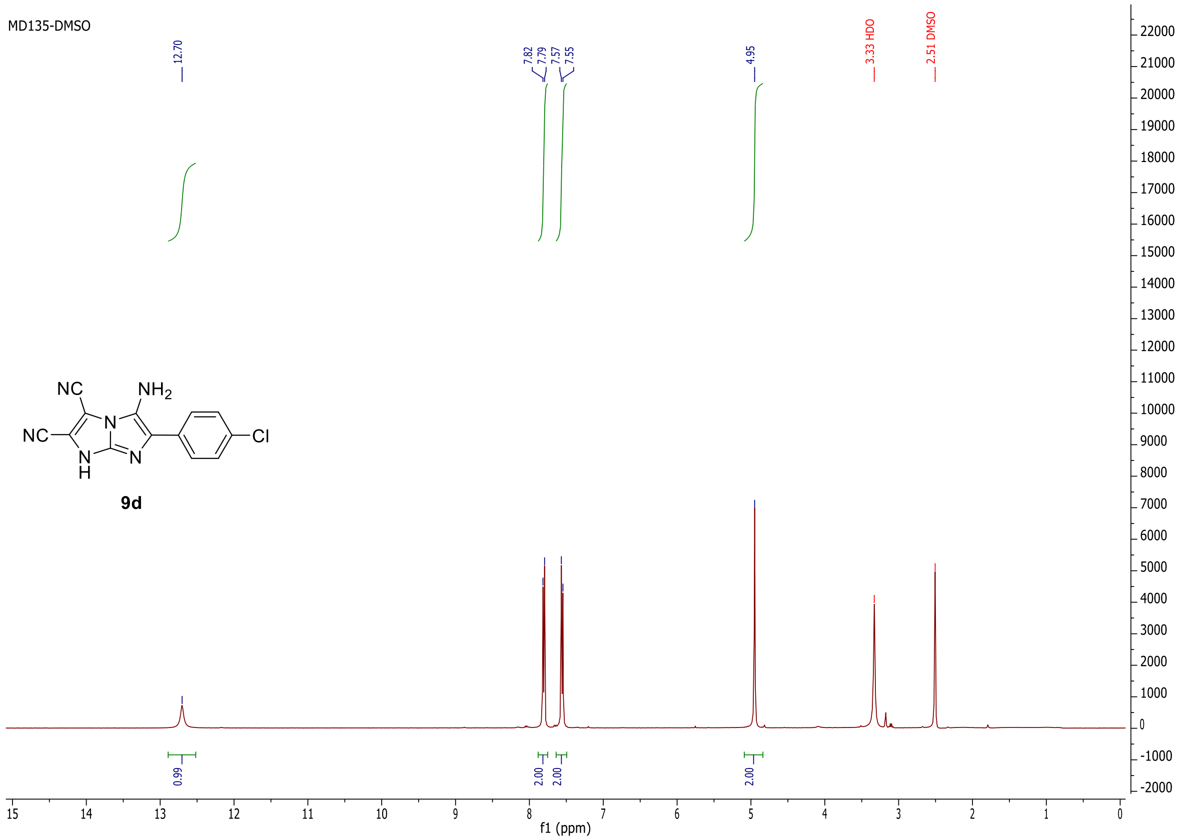
MD135-DMSO
12.70
7.82
7.79
7.57
7.55
4.95
3.33 HDO
2.51 DMSO
NC
NH2
NC
N
N
H
N
Cl
9d
0.99
2.00
2.00
2.00
f1 (ppm)

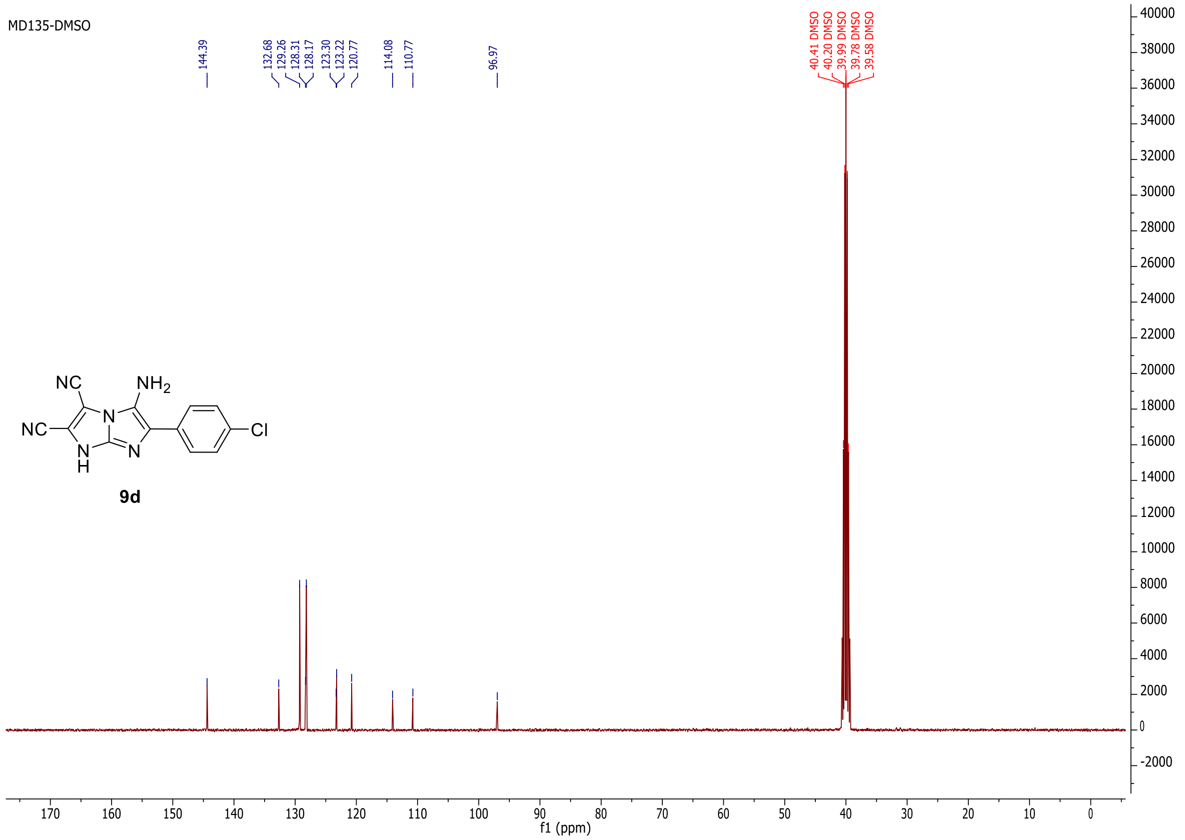
MD135-DMSO
144.39
132.68
129.26
128.31
128.17
123.30
123.22
120.77
114.08
110.77
96.97
40.41 DMSO
40.20 DMSO
39.99 DMSO
39.78 DMSO
39.58 DMSO
NC
NH2
NC
N
N
H
N
Cl
9d
f1 (ppm)

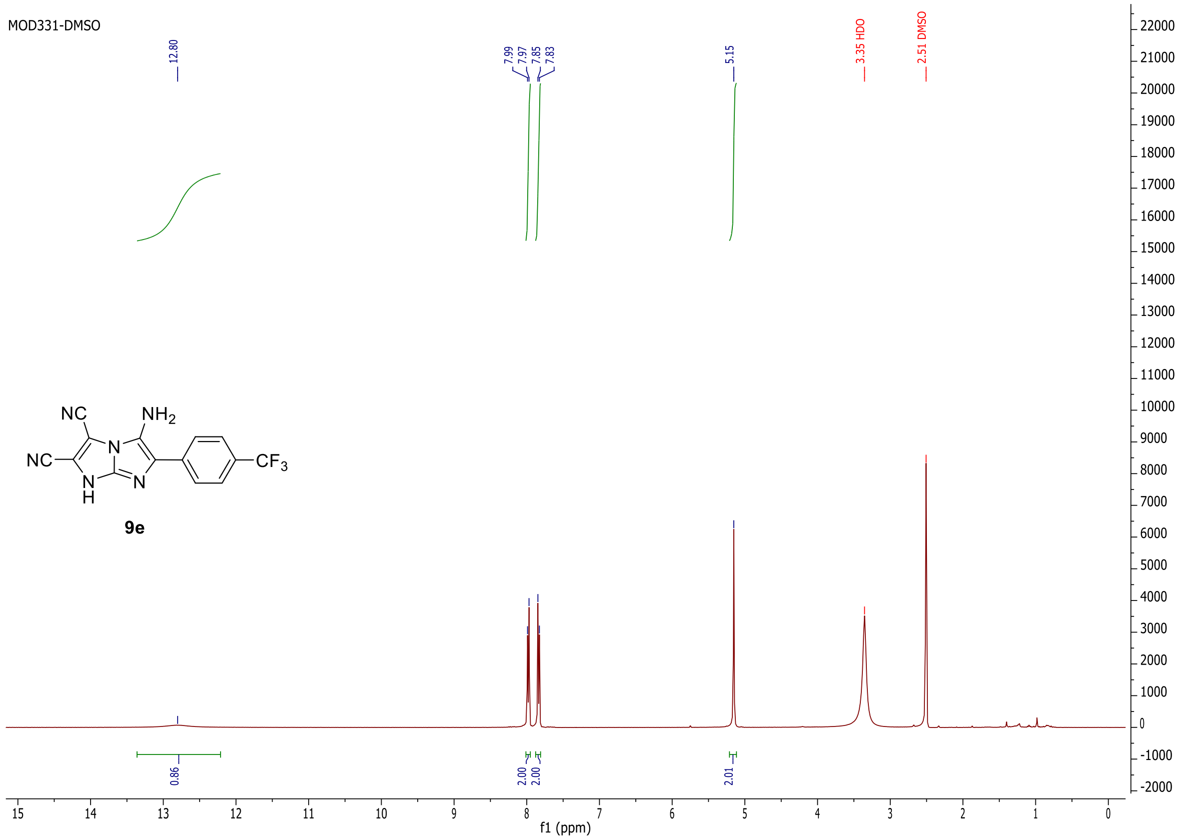
MOD331-DMSO
12.80
7.99
7.97
7.85
7.83
5.15
3.35 HDO
2.51 DMSO
NC
NH2
NC
CF3
N
H
N
N
9e
0.86
2.00
2.00
2.01
f1 (ppm)

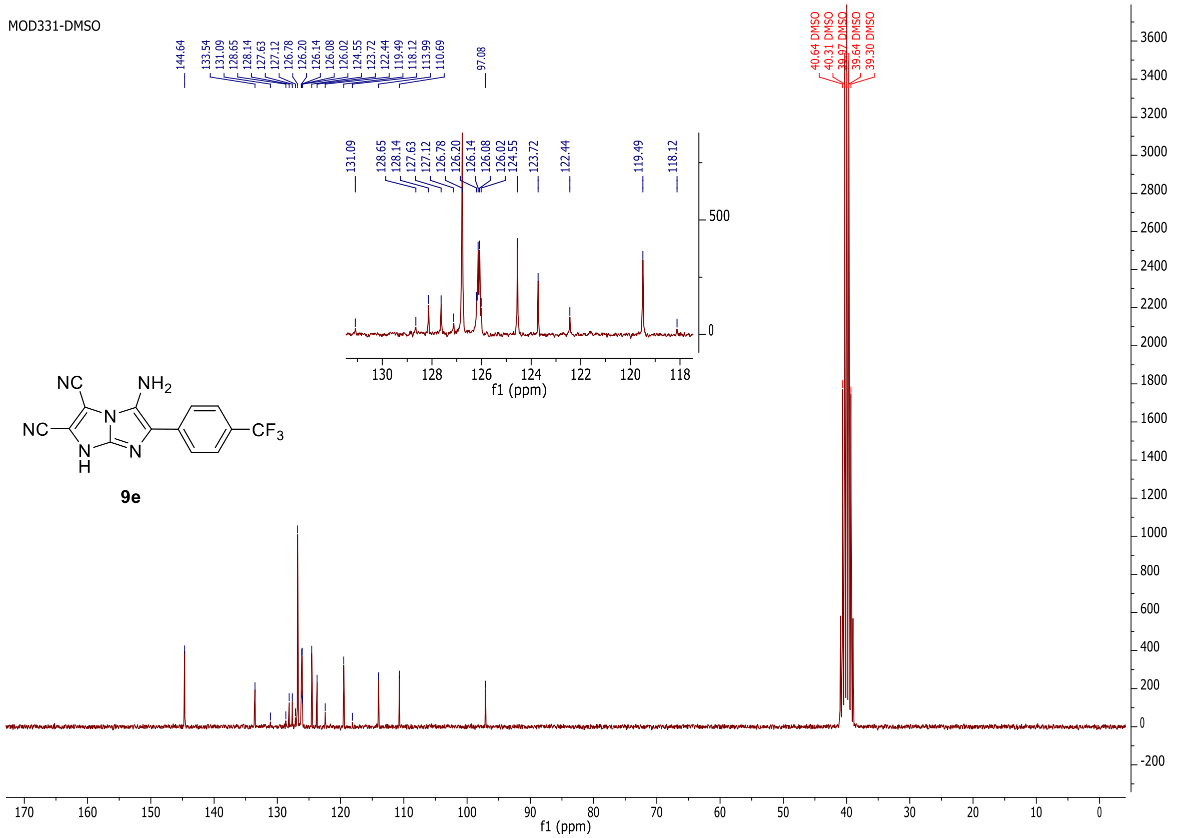
MOD331-DMSO
144.64
133.54
131.09
128.65
128.14
127.63
127.12
126.78
126.20
126.14
126.08
126.02
124.55
123.72
122.44
119.49
118.12
113.99
110.69
97.08
40.64 DMSO
40.31 DMSO
39.97 DMSO
39.64 DMSO
39.30 DMSO
NC
NH2
NC
CF3
N
H
N
N
9e
f1 (ppm)

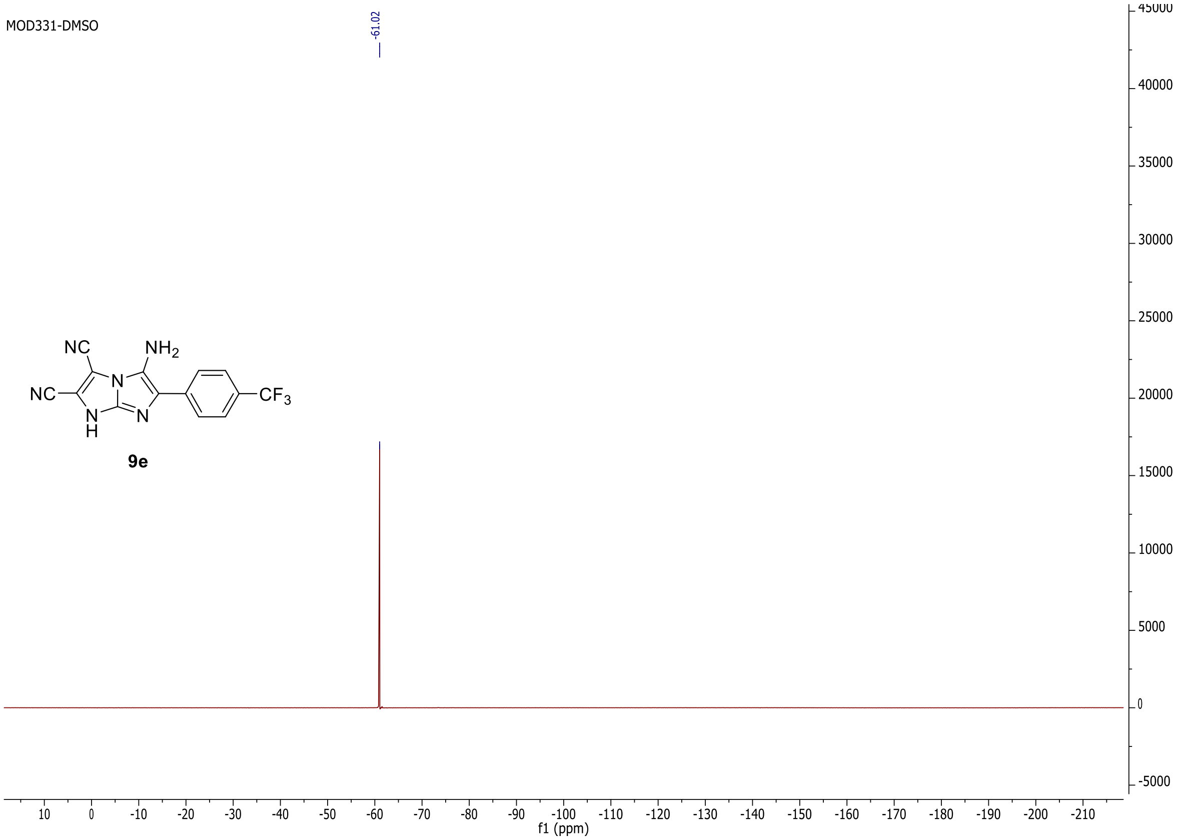
MOD331-DMSO
-61.02
NC
NH2
NC
CF3
N
H
N
9e
45000
40000
35000
30000
25000
20000
15000
10000
5000
0
-5000
10 0 -10 -20 -30 -40 -50 -60 -70 -80 -90 -100 -110 -120 -130 -140 -150 -160 -170 -180 -190 -200 -210
f1 (ppm)

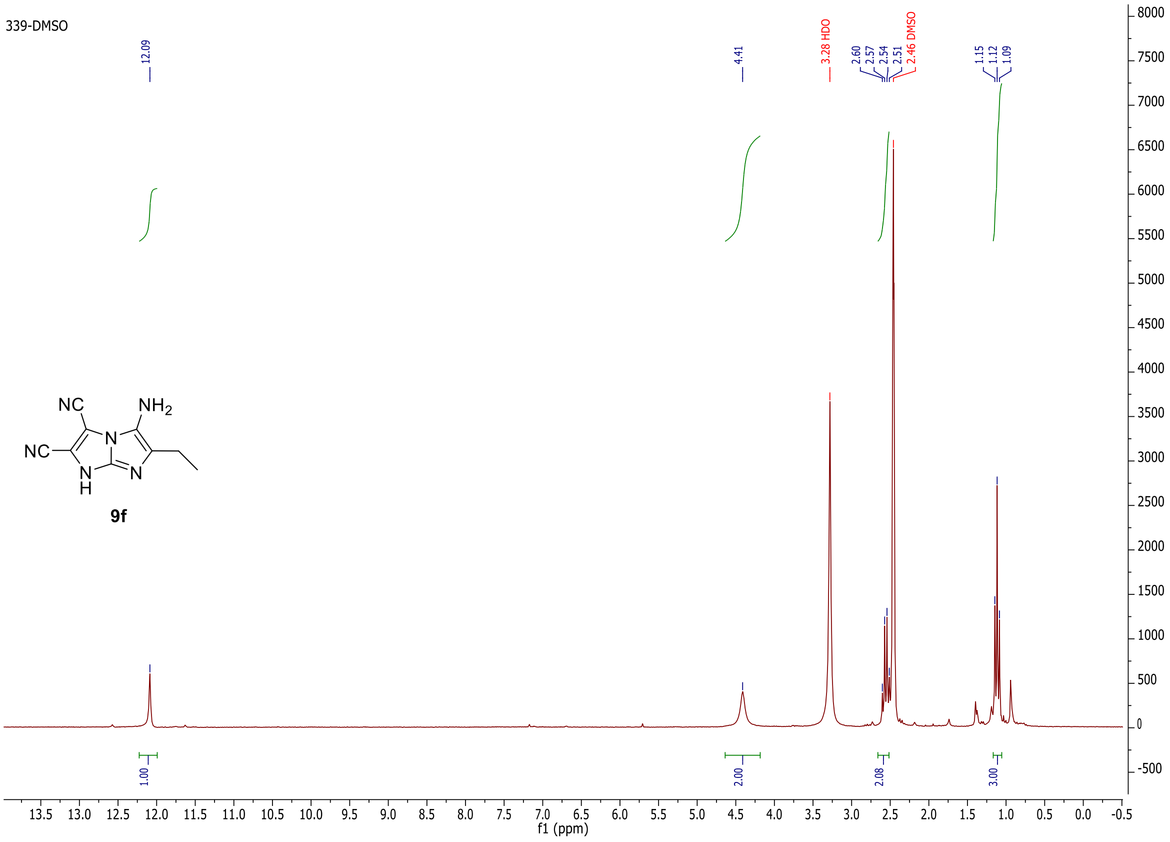
339-DMSO
12.09
4.41
3.28 HDO
2.60
2.57
2.54
2.51
2.46 DMSO
1.15
1.12
1.09
NC
NH2
NC
N
H
N
N
9f
1.00
2.00
2.08
3.00
f1 (ppm)

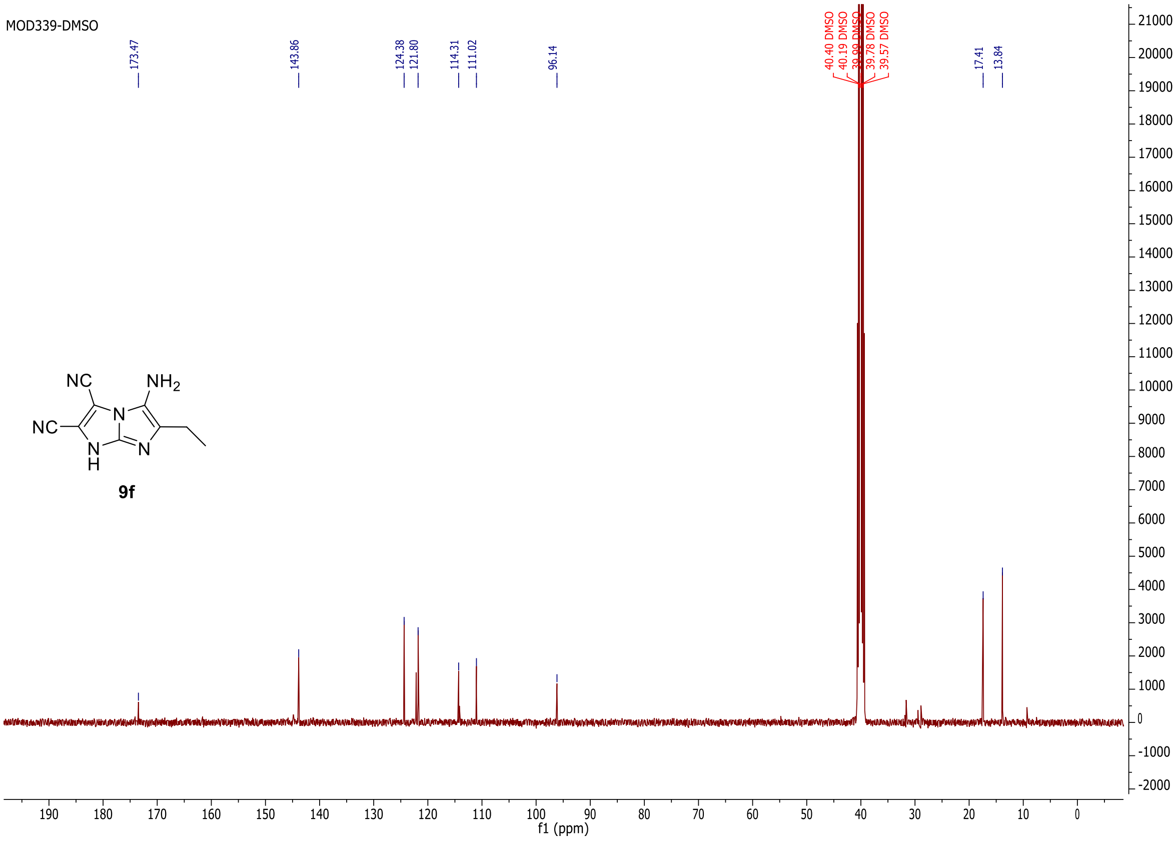
MOD339-DMSO
173.47
143.86
124.38
121.80
114.31
111.02
96.14
40.40 DMSO
40.19 DMSO
39.99 DMSO
39.78 DMSO
39.57 DMSO
17.41
13.84
NC
NH2
NC
N
H
N
N
9f
f1 (ppm)

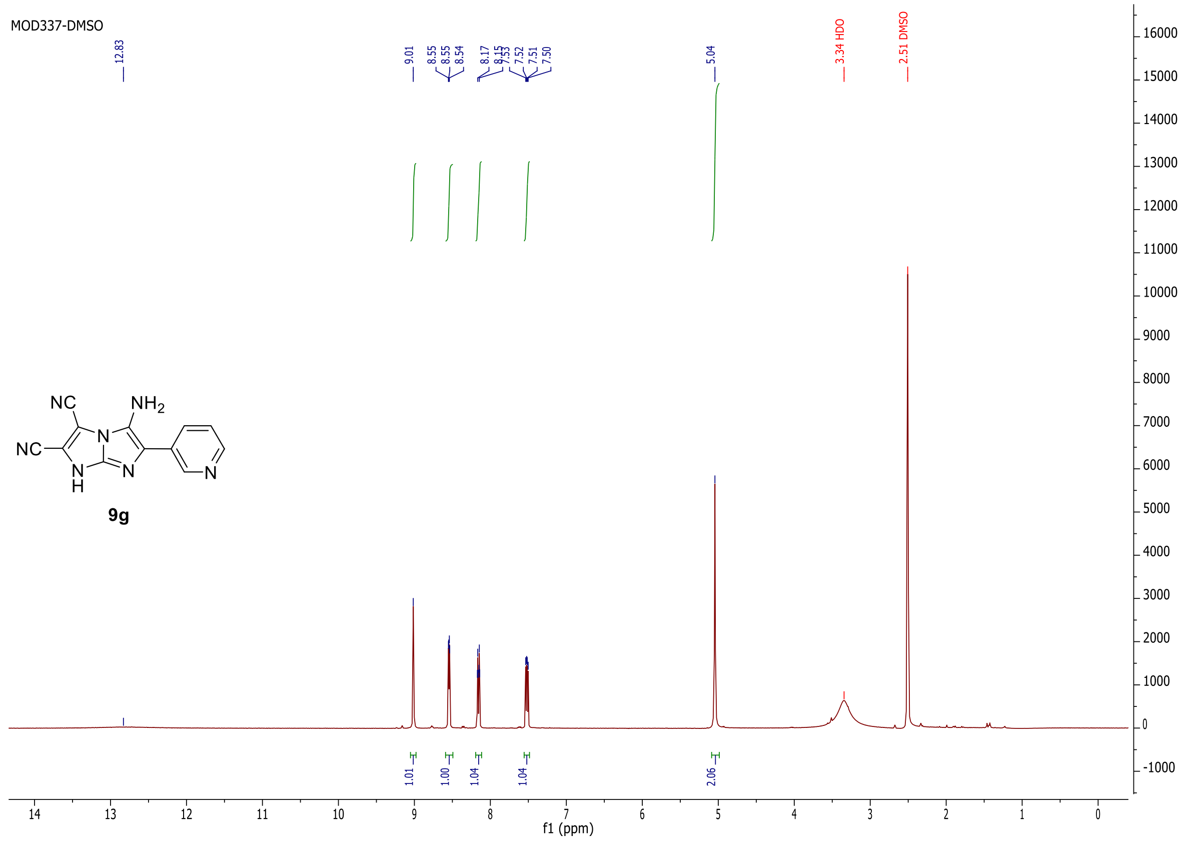
MOD337-DMSO
12.83
9.01
8.55
8.55
8.54
8.17
8.15
7.53
7.52
7.51
7.50
5.04
3.34 HDO
2.51 DMSO
1.01
1.00
1.04
1.04
2.06
NC
NH2
NC
H
N
N
N
N
9g
f1 (ppm)

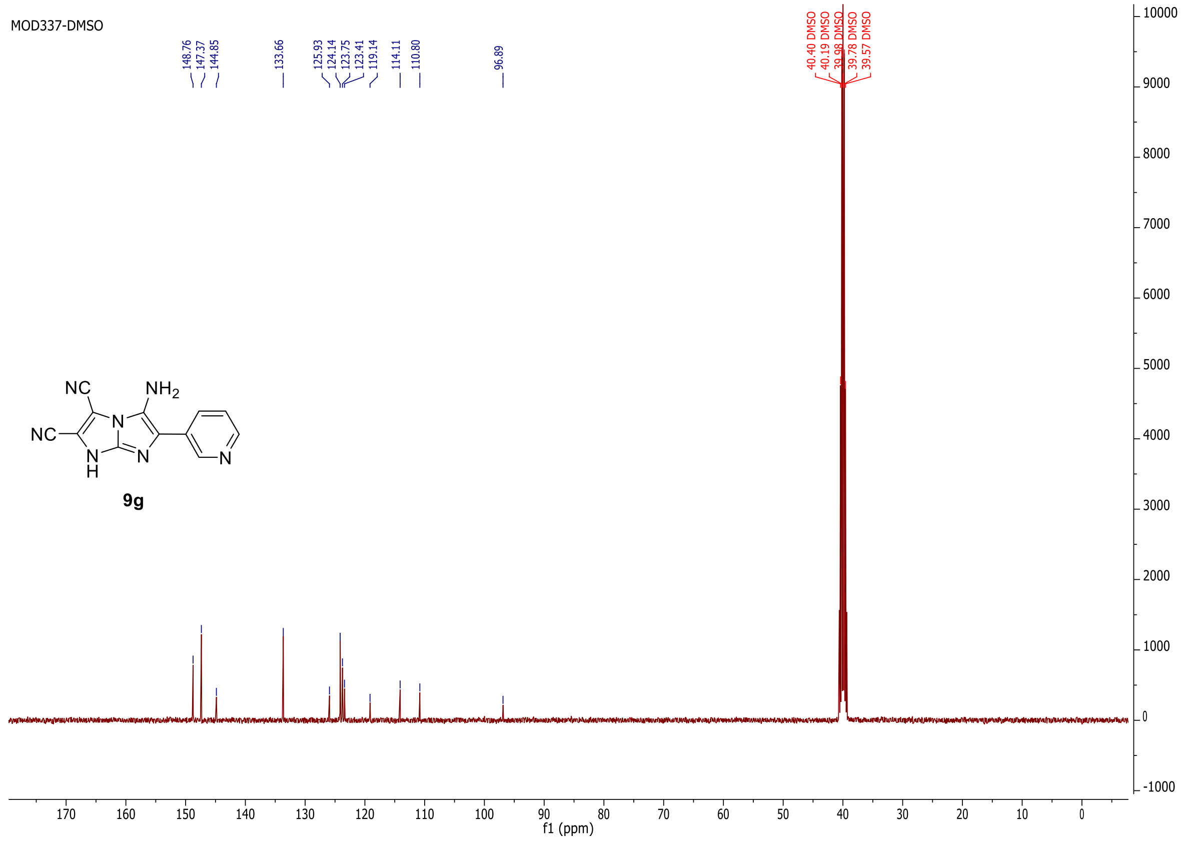
MOD337-DMSO
148.76
147.37
144.85
133.66
125.93
124.14
123.75
123.41
119.14
114.11
110.80
96.89
40.40 DMSO
40.19 DMSO
39.98 DMSO
39.78 DMSO
39.57 DMSO
NC
NH2
NC
H
N
N
N
N
9g
f1 (ppm)

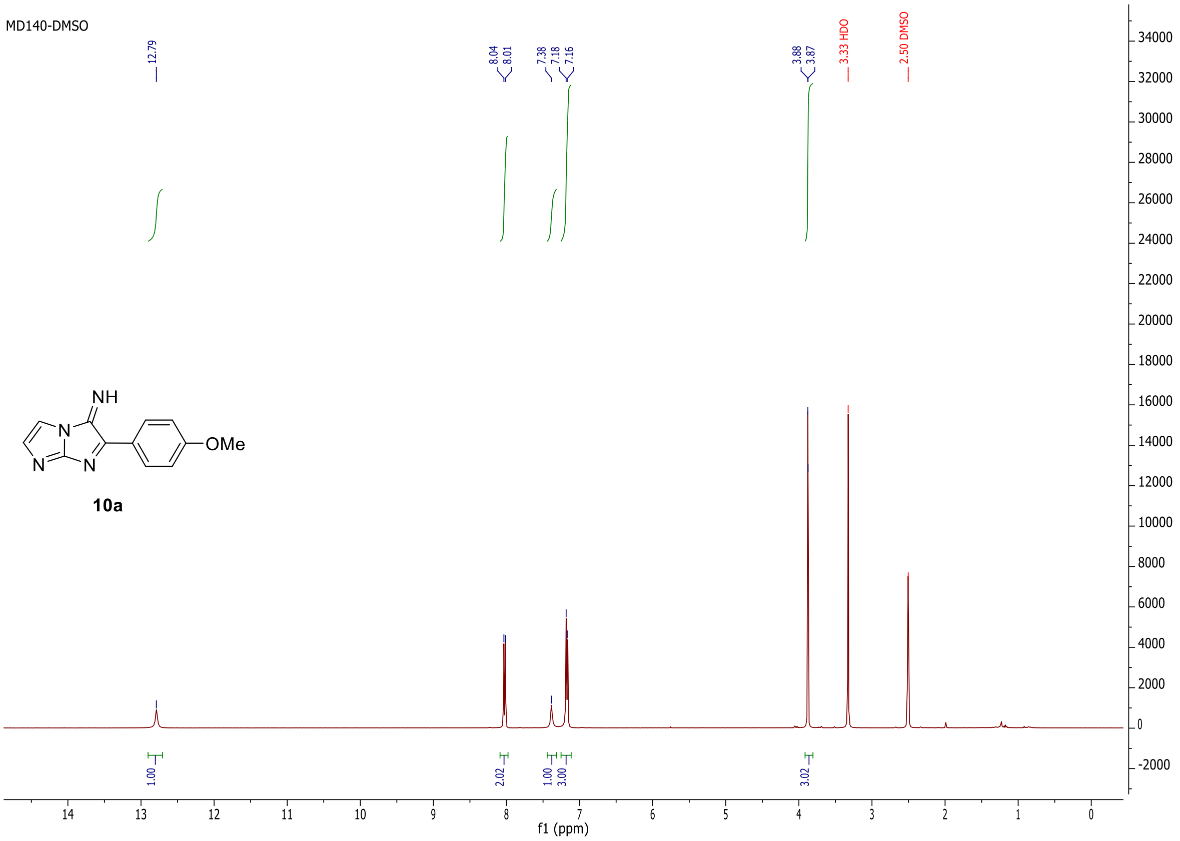
MD140-DMSO
12.79
8.04
8.01
7.38
7.18
7.16
3.88
3.87
3.33 HDO
2.50 DMSO
NH
N
N
N
OMe
10a
1.00
2.02
1.00
3.00
3.02
f1 (ppm)

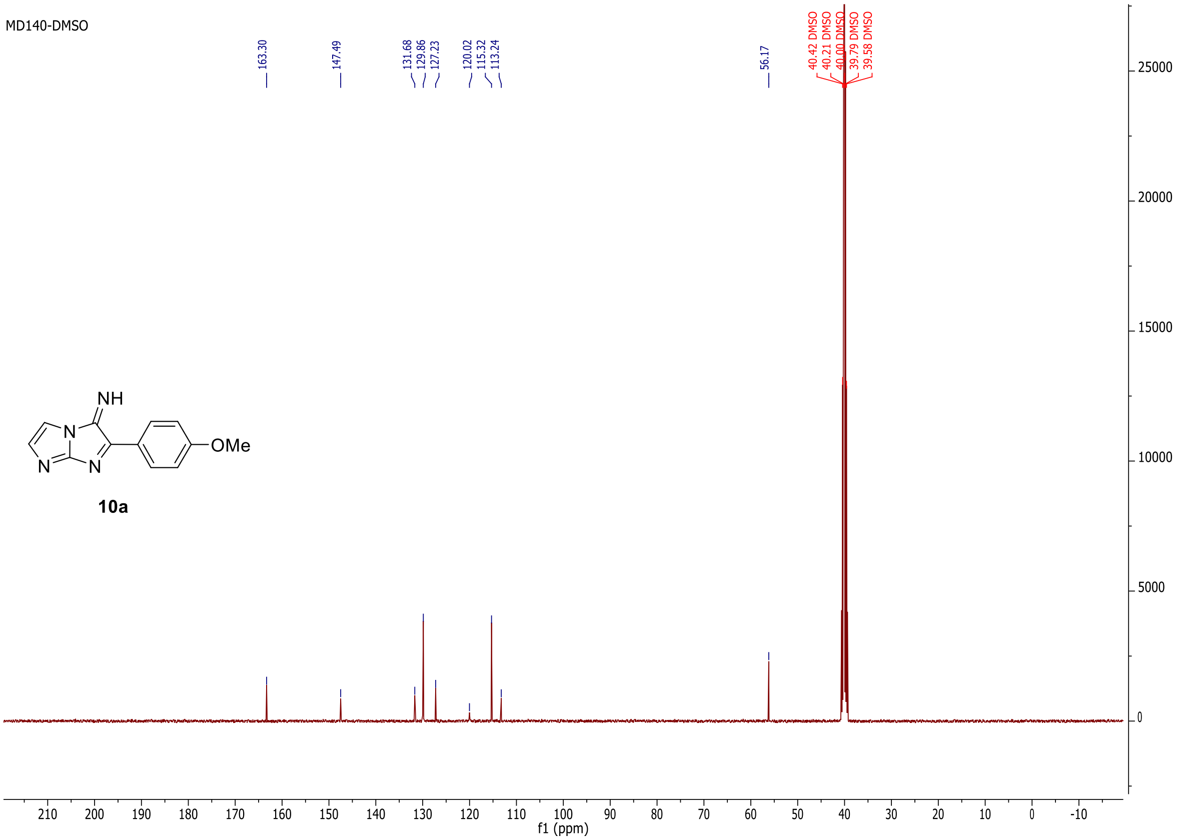
MD140-DMSO
163.30
147.49
131.68
129.86
127.23
120.02
115.32
113.24
56.17
40.42 DMSO
40.21 DMSO
40.00 DMSO
39.79 DMSO
39.58 DMSO
NH
N
N
N
OMe
10a
f1 (ppm)

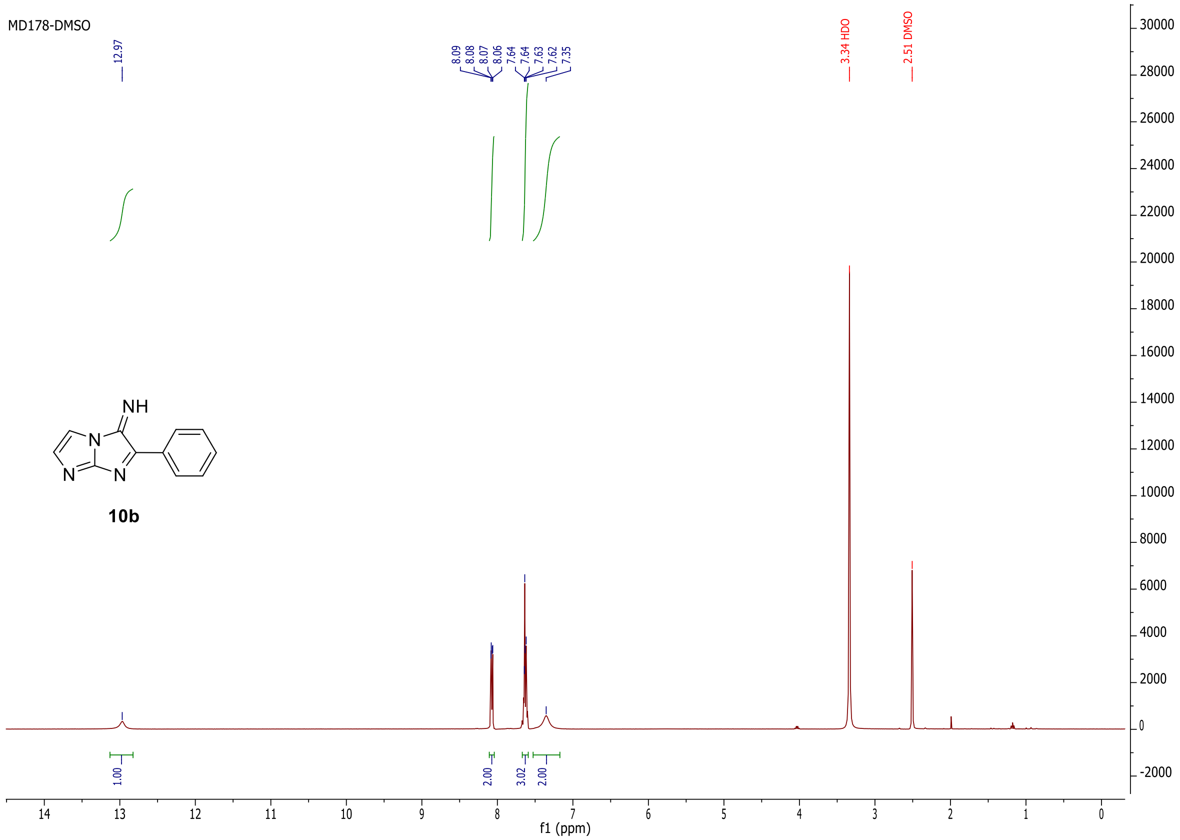

MD178-DMSO
12.97
8.09
8.08
8.07
8.06
7.64
7.64
7.63
7.62
7.35
3.34 HDO
2.51 DMSO
NH
N
N
N
10b
1.00
2.00
3.02
2.00
f1 (ppm)

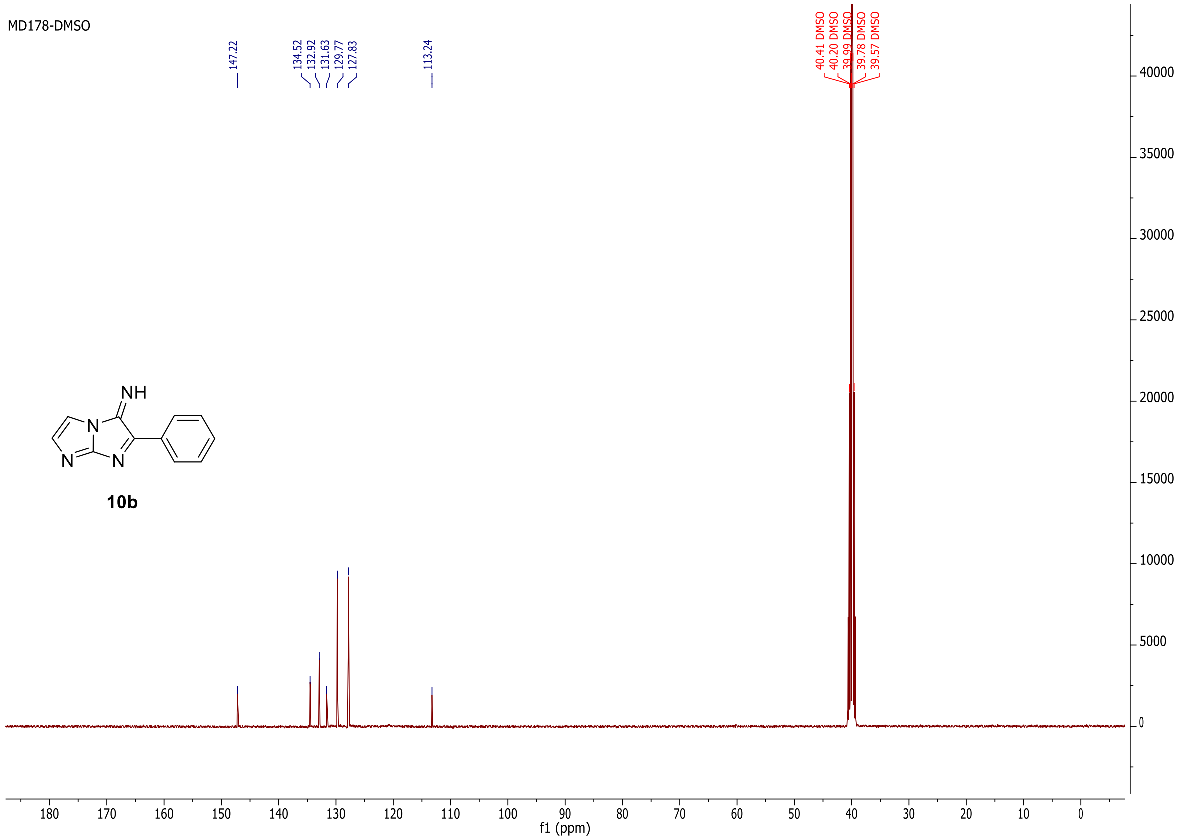

MD178-DMSO
147.22
134.52
132.92
131.63
129.77
127.83
113.24
40.41 DMSO
40.20 DMSO
39.99 DMSO
39.78 DMSO
39.57 DMSO
NH
N
N
N
10b
f1 (ppm)

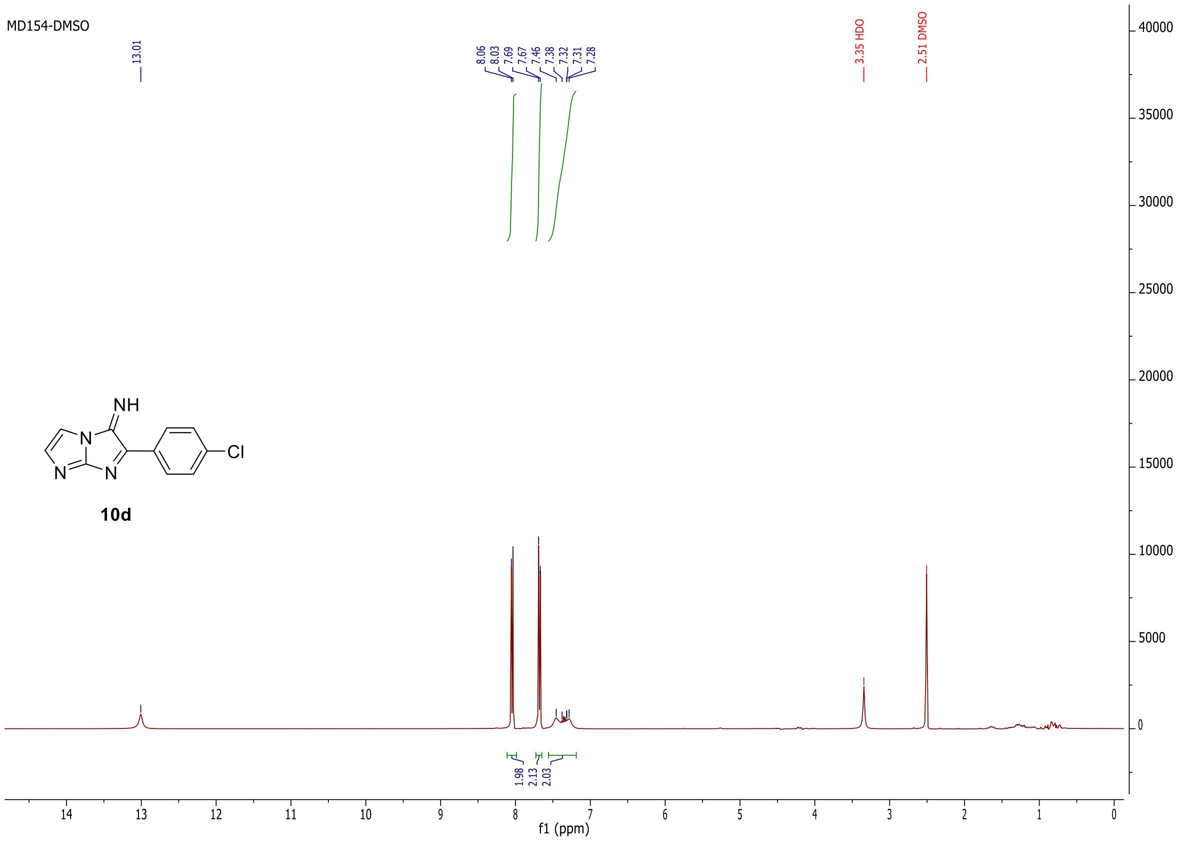
MD154-DMSO
13.01
8.06
8.03
7.69
7.67
7.46
7.38
7.32
7.31
7.28
3.35 HDO
2.51 DMSO
NH
N
N
N
Cl
10d
1.98
2.13
2.03
f1 (ppm)

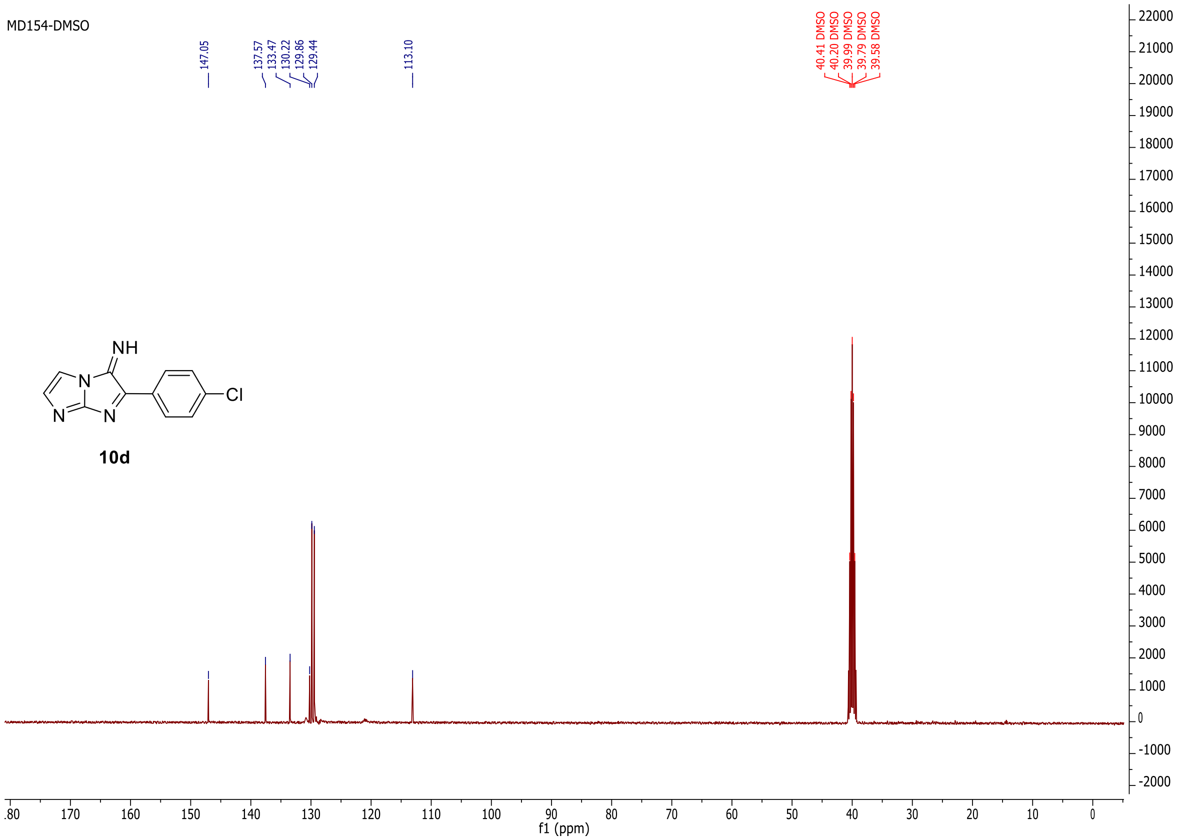
MD154-DMSO
147.05
137.57
133.47
130.22
129.86
129.44
113.10
40.41 DMSO
40.20 DMSO
39.99 DMSO
39.79 DMSO
39.58 DMSO
NH
N
N
N
Cl
10d
f1 (ppm)

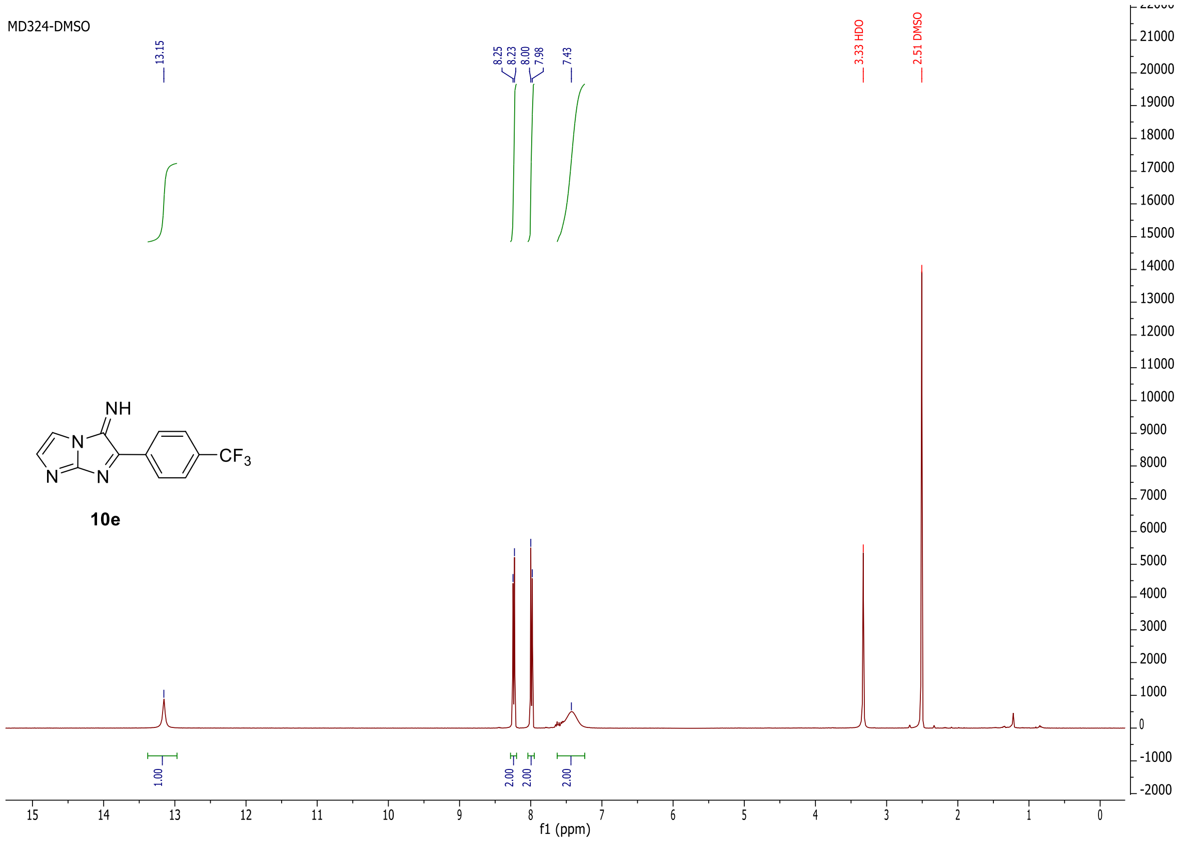
MD324-DMSO
13.15
8.25
8.23
8.00
7.98
7.43
3.33 HDO
2.51 DMSO
NH
N
N
N
CF3
10e
1.00
2.00
2.00
2.00
f1 (ppm)

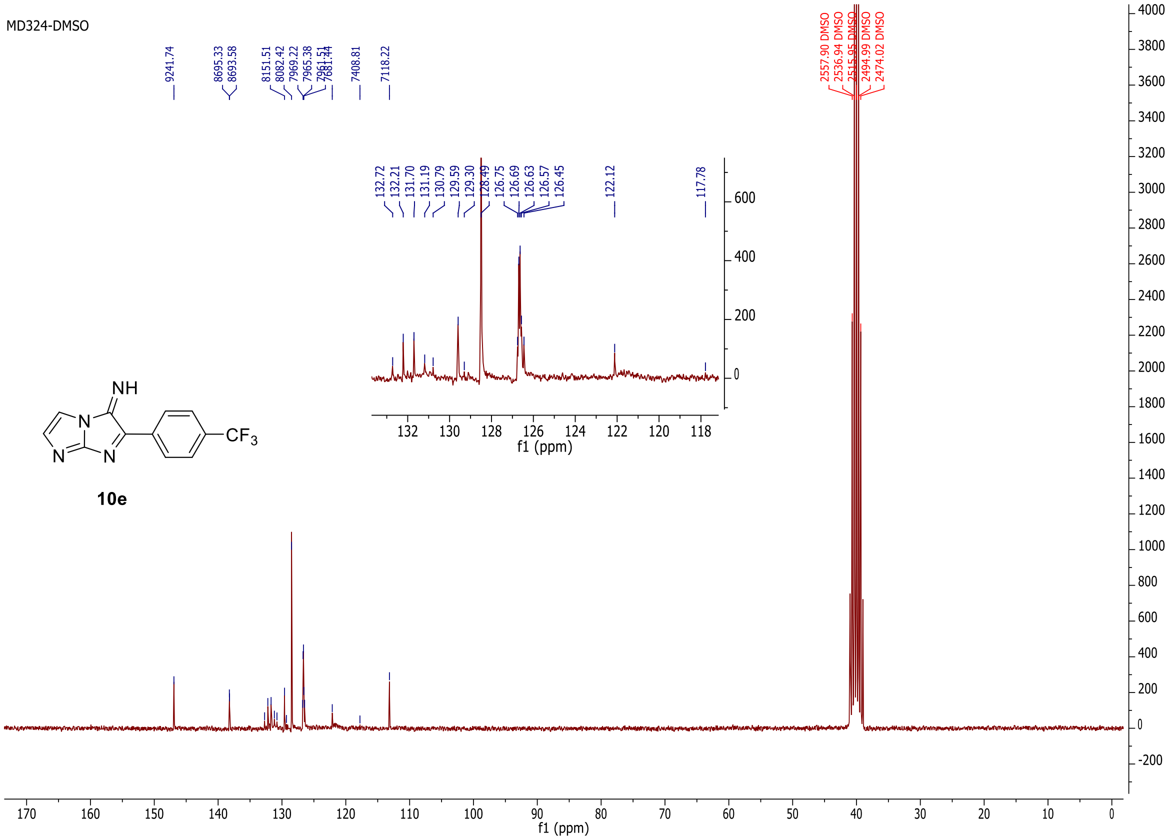
MD324-DMSO
9241.74
8695.33
8693.58
8151.51
8082.42
7969.22
7965.38
7408.81
7118.22
2557.90 DMSO
2536.94 DMSO
2494.99 DMSO
2474.02 DMSO
132.72
132.21
131.70
131.19
130.79
129.59
129.30
126.75
126.69
126.63
126.57
126.45
122.12
117.78
f1 (ppm)
NH
N
N
N
CF3
10e
f1 (ppm)

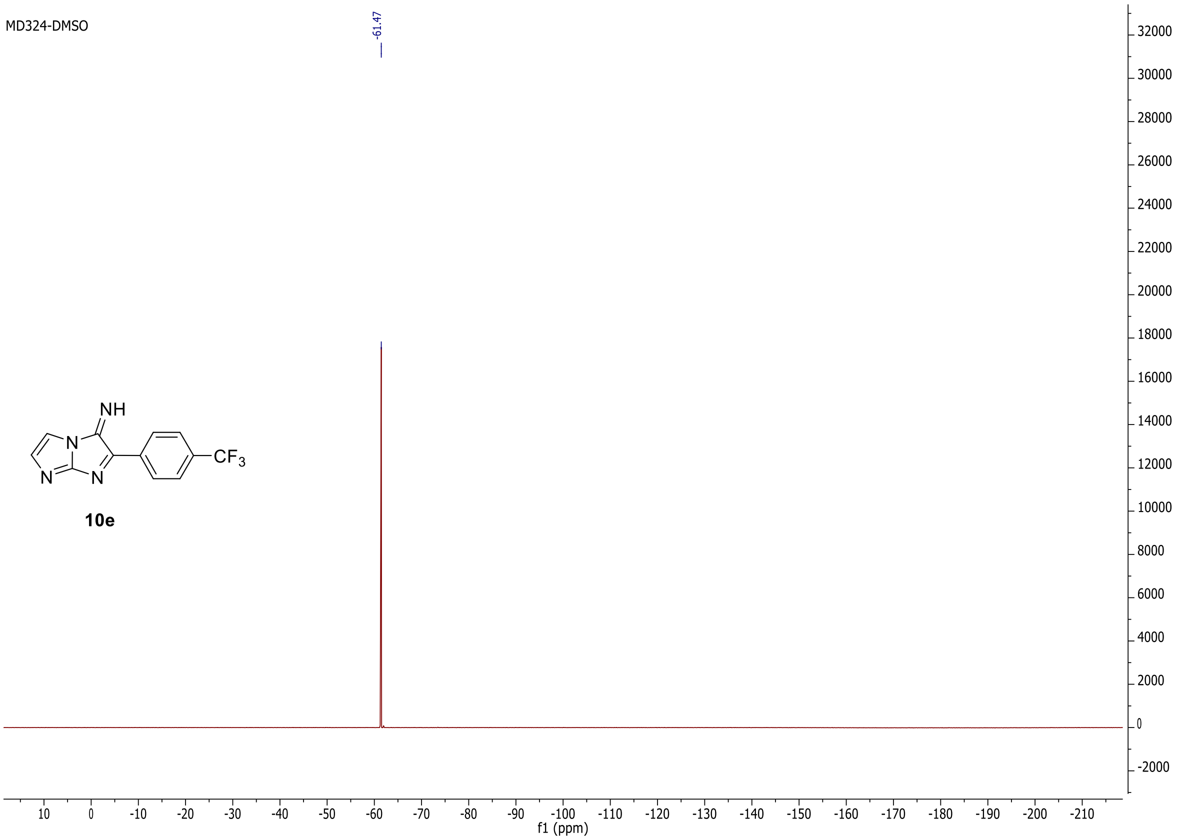
MD324-DMSO
-61.47
NH
N
N
N
CF3
10e
32000
30000
28000
26000
24000
22000
20000
18000
16000
14000
12000
10000
8000
6000
4000
2000
0
-2000
10
0
-10
-20
-30
-40
-50
-60
-70
-80
-90
-100
-110
-120
-130
-140
-150
-160
-170
-180
-190
-200
-210
f1 (ppm)

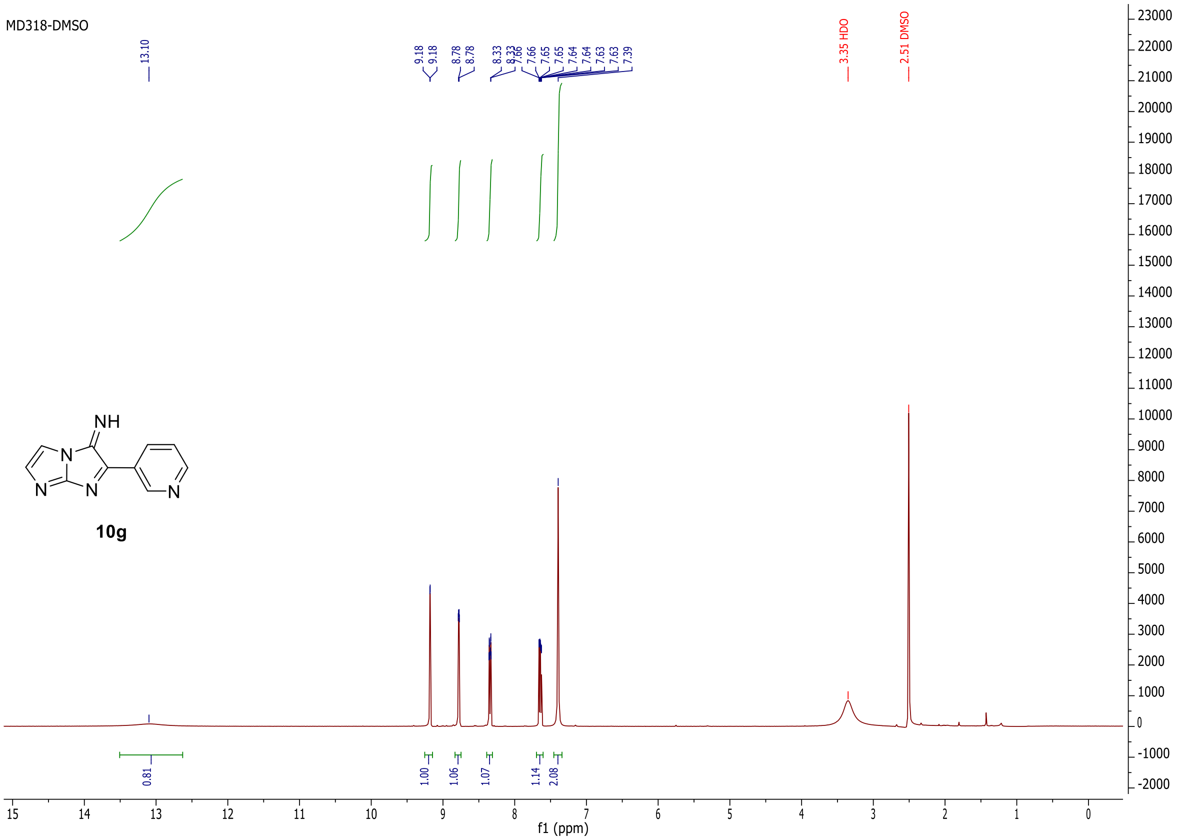
MD318-DMSO
13.10
9.18
9.18
8.78
8.78
8.33
8.33
7.66
7.66
7.65
7.65
7.64
7.64
7.63
7.63
7.39
3.35 HDO
2.51 DMSO
NH
N
N
N
N
10g
0.81
1.00
1.06
1.07
1.14
2.08
f1 (ppm)

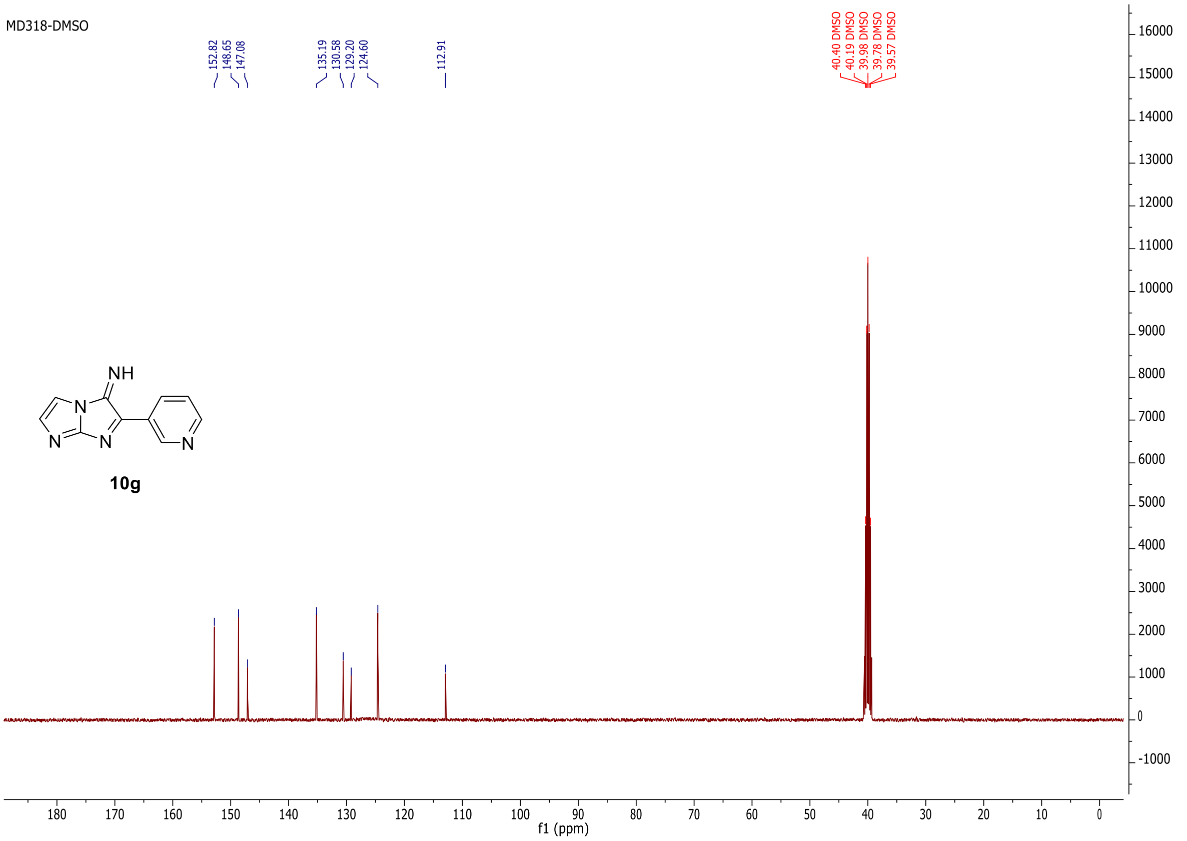
MD318-DMSO
152.82
148.65
147.08
135.19
130.58
129.20
124.60
112.91
40.40 DMSO
40.19 DMSO
39.98 DMSO
39.78 DMSO
39.57 DMSO
NH
N
N
N
N
10g
f1 (ppm)

X-ray analysis of compound 4a

Slow evaporation of an ethanol solution of **4a** yielded single crystals suitable for X-ray crystallography.

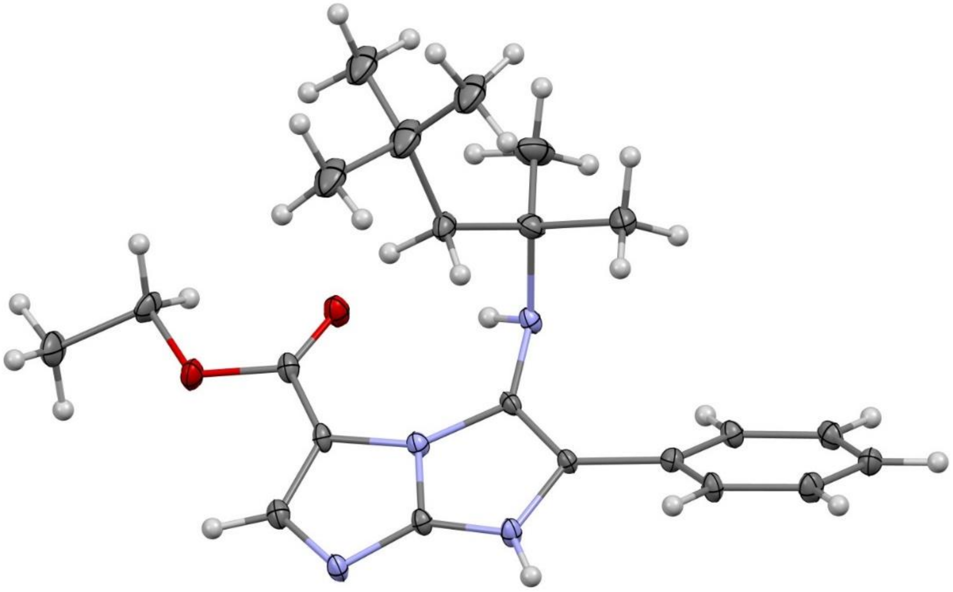

Fig. S01. An ORTEP drawing of compound **4a**. Thermal ellipsoids are shown at the 30% level. Disorder in *t*-octyl is omitted for clarity.

X-ray diffraction data for compound **4a** was collected by using a VENTURE PHOTON100 CMOS Bruker diffractometer with Micro-focus IuS source Cu Kα radiation. Crystals were mounted on a CryoLoop (Hampton Research) with Paratone-N (Hampton Research) as cryoprotectant and then flashfrozen in a nitrogen-gas stream at 100 K. For compounds, the temperature of the crystal was maintained at the selected value by means of a 700 series Cryostream cooling device to within an accuracy of ±1K. The data were corrected for Lorentz polarization, and absorption effects. The structures were solved by direct methods using SHELXS-97[1] and refined against F^2 by full-matrix least-squares techniques using SHELXL-2018[2] with anisotropic displacement parameters for all non-hydrogen atoms. Hydrogen atoms were located on a difference Fourier map and introduced into the calculations as a riding model with isotropic thermal parameters. All calculations were performed by using the Crystal Structure crystallographic software package WINGX.[3]

The *t*-octyl is disordered over two orientations; the final refined occupancy factors of the two components of disorder are 0.694(4) and 0.306(4).

The crystal data collection and refinement parameters are given in Table S1.

---

[1] Sheldrick, G. M. SHELXS-97, Program for Crystal Structure Solution, University of Göttingen, Göttingen, Germany, **1997**.

[2] Sheldrick, G. M. *Acta Crystallogr., Sect. A: Found. Crystallogr.,* **2008**, 64, 112-122.

[3] Farrugia, L. J. *J. Appl. Cryst*. **1999**, *32*, 837.

CCDC 1916259 contains the supplementary crystallographic data for this paper. These data can be obtained free of charge from the Cambridge Crystallographic Data Centre via http://www.ccdc.cam.ac.uk/Community/Requestastructure.

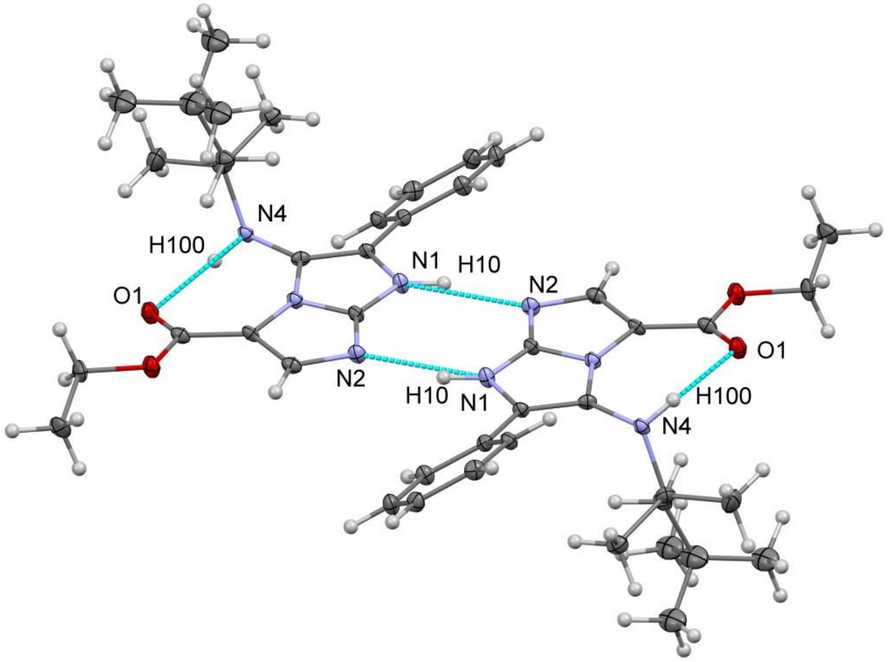

Fig. S02. Part of the crystalline structure of **4a** showing the formation of hydrogen bonds. The intramolecular and intermolecular hydrogen bonds are drawn in blue dashed lines.

The crystal structure of compound **4a** is further stabilized by two N-H…X interactions (table S2).

Table S1. Crystallographic data and structure refinement details.

**Compound**	**4a**
Empirical Formula	$C_{22} H_{30} N_4 O_2$
M_r	382.50
Crystal size, mm^3	0.35 x 0.02 x 0.02
Crystal system	monoclinic
Space group	*C* 2/*c*
a, Å	29.4080(12)
b, Å	6.4911(2)
c, Å	26.8178(9)
α, °	90
β, °	122.192(2)

γ, °	90
Cell volume, Å^3	4332.3(3)
Z ; Z’	8 ; 1
T, K	100 (1)
Radiation type ; wavelength Å	CuKα; 1.54178
F_{000}	1648
μ, mm^{-1}	0.609
θ range, °	3.552 - 59.053
Reflection collected	27 162
Reflections unique	3 114
R_{int}	0.0906
GOF	1.031
Refl. obs. (I>2σ(I))	1 961
Parameters / restraints	257 / 11
wR_2 (all data)	0.1715
R value (I>2σ(I))	0.0657
Largest diff. peak and hole (e-.Å^{-3})	0.442 ; -0.673

Table S2. Geometric parameters (Å, degree) for intra- and intermolecular interactions

Donor-H....Acceptor	*D - H*	*H...A*	*D...A*	*D - H...A*
N1-H10...N2$^{(i)}$	0.88	1.93	2.7837(1)	162
N4-H100..O1	0.85	2.21	2.9606(1)	149

Symmetry codes: *i* = 1-x,-y,-z
